# Supplementary material for: Neoadjuvant palbociclib and endocrine therapy versus chemotherapy in ER + /HER2- breast cancer: a randomized phase II trial
Source: Nat Commun. 2026 Apr 8;17:3403. doi: 10.1038/s41467-026-71452-6 (PMC13069018; doi:10.1038/s41467-026-71452-6)
Supplement: Supplementary file 1 — Supplementary Information [file 41467_2026_71452_MOESM1_ESM.pdf]

Data Supplement to

Neoadjuvant palbociclib and endocrine therapy versus chemotherapy in ER+/HER2- breast cancer: a randomized phase II trial

Alexios Matikas, Evangelos Tzoras, Michail Sarafidis, Emmanouil G. Sifakis, Judith Björle, Elin Barnekow, Sara Margolin, Erika Isaksson-Friman, Luisa Edman Kessler, Athanasios Zouzos, Hemming Johansson, Mats Hellström, Per Grybäck, Dimitrios Salgkamis, Ioannis Zerdes, Kang Wang, Johan Hartman, Balazs Acs, Wenwen Sun, Ceren Boyaci, Guillermo Villacampa, Tomas Pascual, Joaquin Gavila, Aleix Prat, Charles Perou, Yvonne Brandberg, Jonas Bergh, Thomas Hatschek, Theodoros Foukakis

Table of Contents

Supp. Fig. 1: KM curves of EFS, RFS and OS 2
Supp. Fig. 2: Forest plots for ORR_{12} according to CDK4/6-Rb pathway alterations 3
Supp. Fig. 3: DEGs and GSEA analysis in responders vs. non-responders for ORR_{12} 4
Supp. Fig. 4: Forest plots for ORR_{12} according to ER activity. 5
Supp. Fig. 5: Forest plots for ORR_{12} according to antigen presentation 6
Supp. Fig. 6: Forest plots for ORR_{12} according to T cell activity 7
Supp. Fig. 7: Forest plots for ORR_{12} according to humoral immune activity 8
Supp. Fig. 8: Forest plots for ORR_{12} according to BC360 modules 9
Supp. Fig. 9: GSEA in patients with RCB 0/1 vs. RCB 2/3 10
Supp. Fig. 10: Correlations between clinicopathologic features and CDKPredX groups 11
Supp. Fig. 11: Correlations between RB1 status, RBsig, E2Fsig, and CDKPredX groups 12
Supp. Fig. 12: Comparison of 21-gene Recurrence Score distributions by CDKPredX groups 13
Supp. Fig. 13: Forest plots for ORR_{12} according to CDKPredX groups per menopausal status 14
Supp. Fig. 14: ORR24, RCB and pCR rates between the CDKPredX groups 15
Supp. Fig. 15: KM curves of EFS, RFS and OS for CDKPredX groups 16
Supp. Fig. 16: Association of CDKPredX and PET/CT SUVmax with outcomes in lobular tumors 17
Supp. Fig. 17: Forest plot for pCR and CDKPredX subgroups within the ER+/HER2- patients of ISPY-2 18
Supp. Fig. 18: KM curves of DRFi according to CDKPredX groups in SCAN-B 19
Supp. Fig. 19: Differences at baseline according to EORTC QLQ-C30 and EORTC QLQ-BR23 20
Supp. Fig. 20: Differences at 12 weeks according to EORTC QLQ-C30 and EORTC QLQ-BR23 21
Supp. Fig. 21: Differences at EOT according to EORTC QLQ-C30 and EORTC QLQ-BR23 22
Supp. Fig. 22: HRQoL at 18 months according to EORTC QLQ-C30 23
Supp. Fig. 23: Histomorphologic assessment of immune cell aggregates 24
Supp. Table 1: Characteristics of the CDKPredX subgroups 25
Supp. Data: CONSORT Checklist 26
Supp. Data: Trial Protocol 28

Supplementary Figure 1: Kaplan-Meier curves of event-free survival (EFS) (A), relapse-free survival (RFS) (B) and overall survival (OS) (C), after a median follow-up of 4.52 years. EFS and OS are calculated in the intention-to-treat population. For the RFS analysis, 3 patients are removed since they did not receive surgery due to drop-out or disease progression.

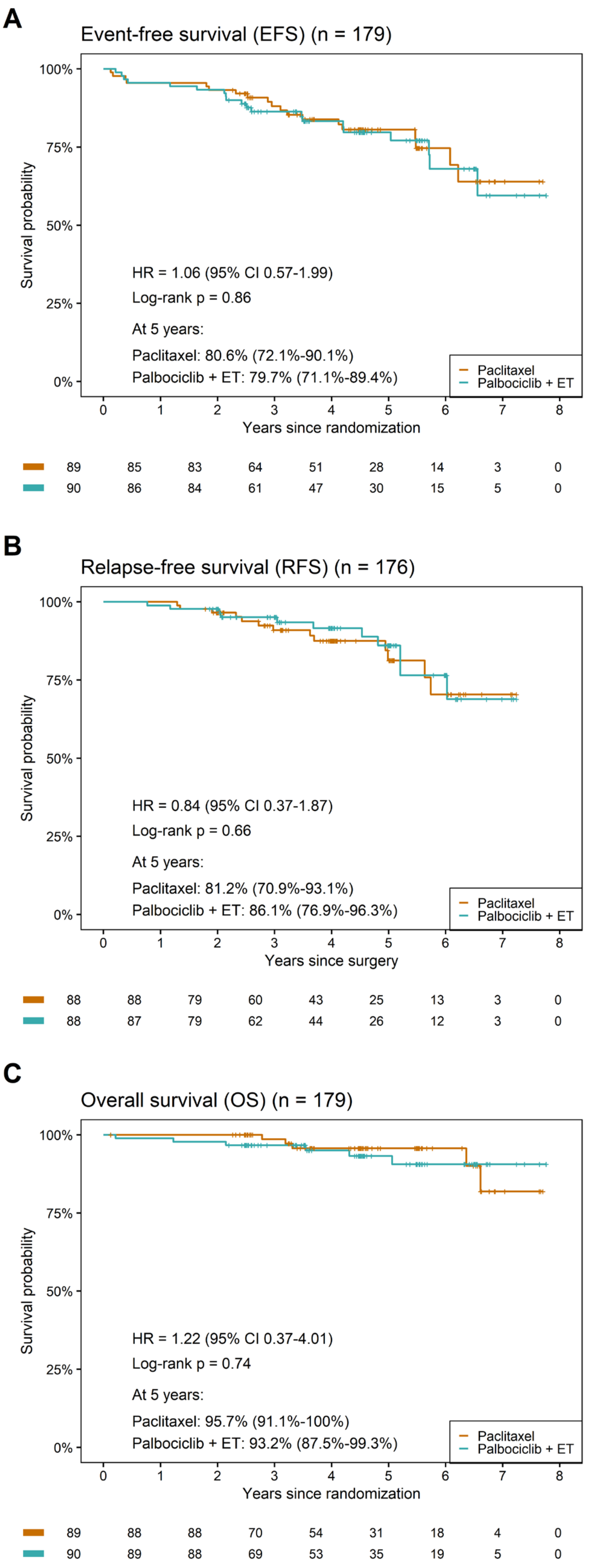

Supplementary Figure 2: Forest plots for the primary endpoint ORR_{12} in PREDIX LumB according to detected somatic mutations with VAF ≥ 5% (A), gains or amplifications (B), and shallow or deep deletions (C) in genes of the CDK4/6-Rb pathway, including *CCNA2*, *CCNB1*, *CCNB2*, *CCNB3*, *CCND1*, *CCND2*, *CCND3*, *CCNE1*, *CCNE2*, *CDK1*, *CDK2*, *CDK3*, *CDK4*, *CDK6*, *CDKN1A*, *CDKN1B*, *CDKN1C*, *CDKN2A*, *CDKN2B*, *CDKN2C*, *CDKN2D*, *E2F1*, *E2F2*, *E2F3*, *RB1*, *RBL1*, and *RBL2*. No point mutations were detected in the *RB1* gene. Copy number alterations were identified using GISTIC2.0 and classified as deep deletion, shallow deletion, neutral, gain, or amplification. Odds ratios were estimated using logistic regression with two-sided Wald tests, interaction effects were evaluated using two-sided likelihood ratio tests, and no adjustment for multiple comparisons was applied. Source data are provided as a Source Data file.

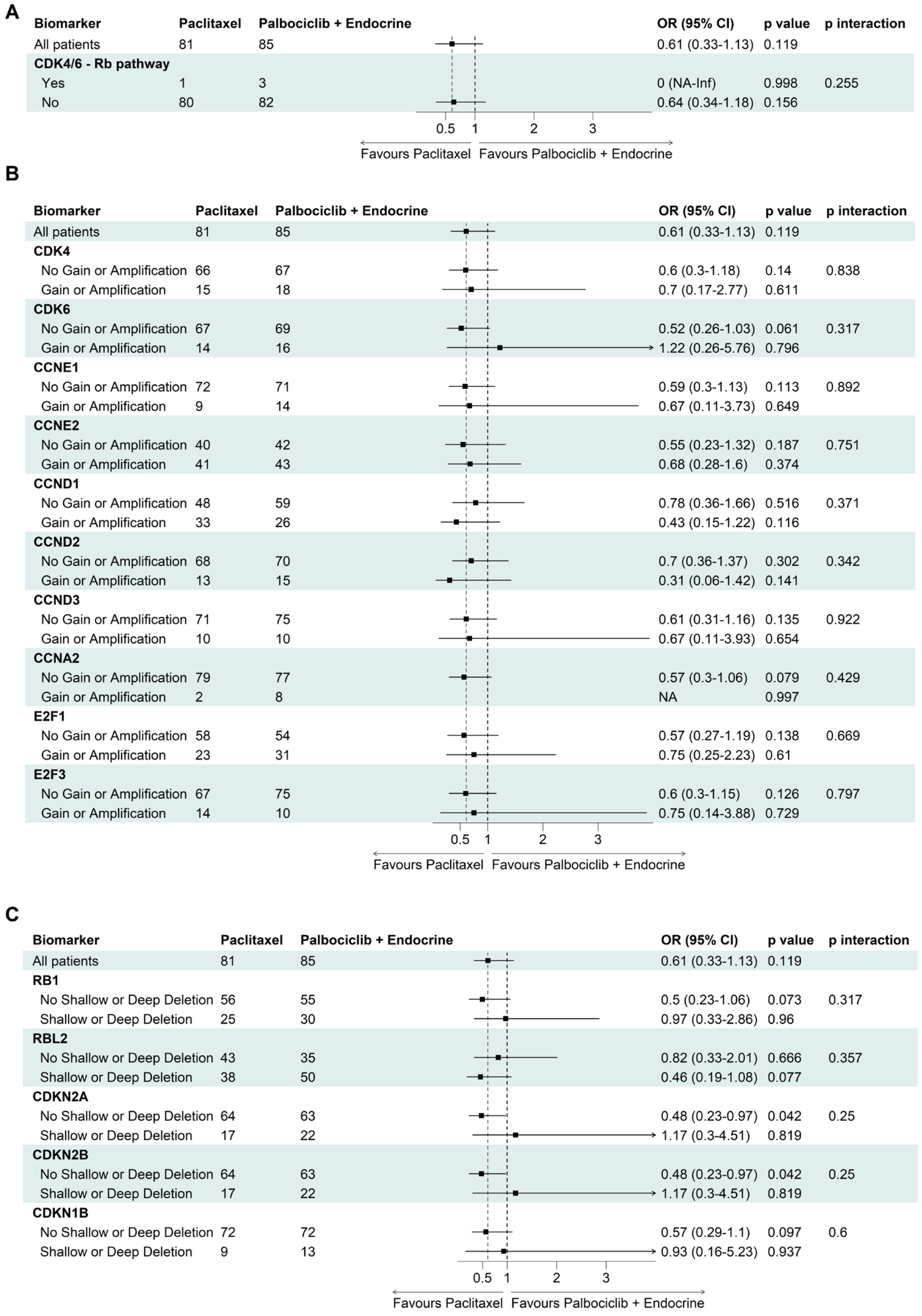

Supplementary Figure 3: Differential gene expression and pathway analysis between responders and non-responders for the primary endpoint ORR_{12} in PREDIX LumB, shown separately for each treatment arm. Volcano plots of differentially expressed genes ($|log_2FC| > 0.5$, p-adj < 0.05) are shown in red (A, D). Hallmark MSigDB gene set enrichment analysis (GSEA) barplots display normalized enrichment scores (NES) for significantly enriched pathways (p-adj < 0.05) (B, E); upregulated pathways are shown in green and downregulated in dark orange. Reactome enrichment maps (top 25 terms) visualize pairwise term similarity and cluster labels (C, F); node size represents the number of genes per pathway and node color indicates the adjusted p-value. Source data are provided as a Source Data file.

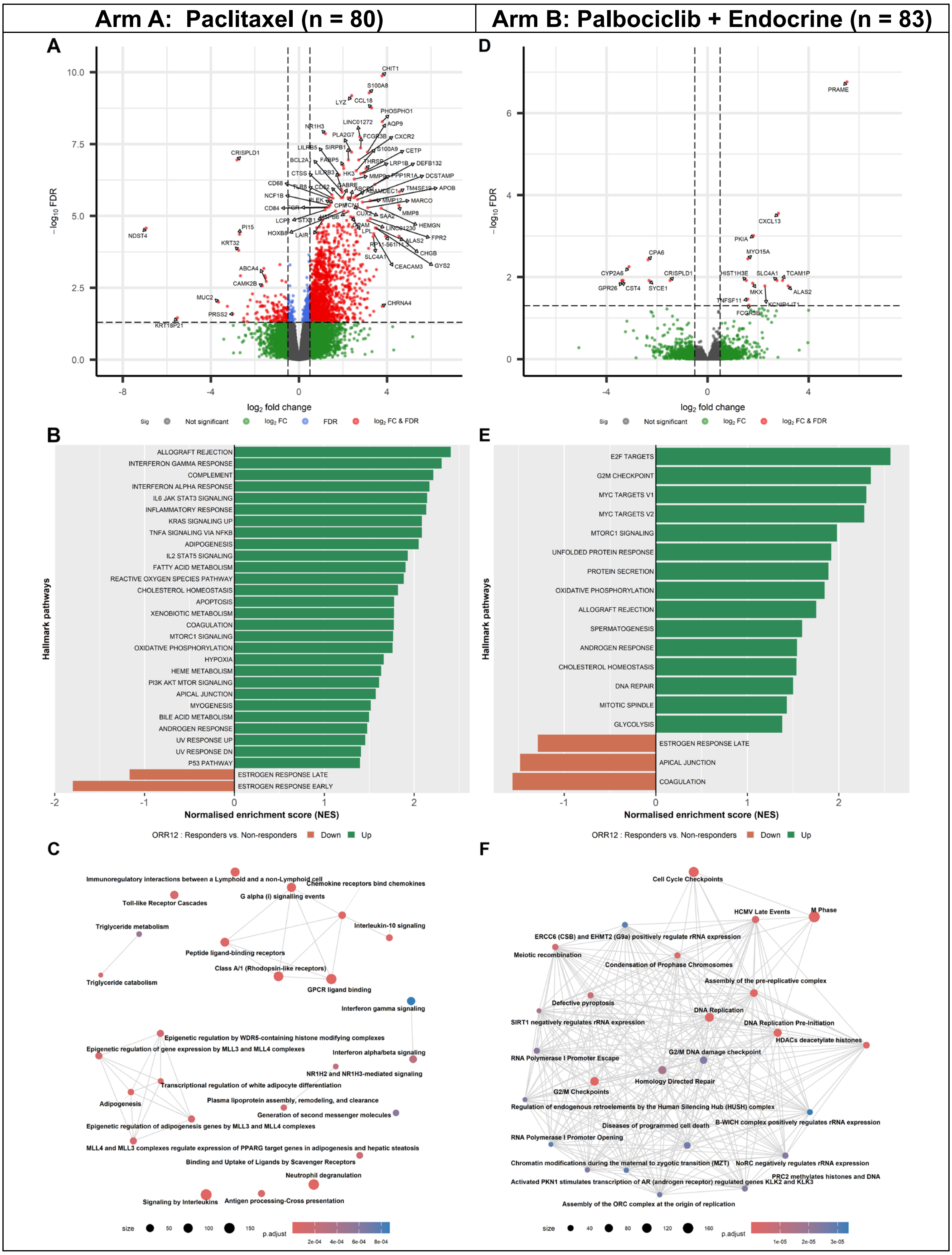

Supplementary Figure 4: Forest plot for the primary endpoint ORR_{12} in PREDIX LumB according to established ER-related gene signatures. For each signature, subgroups were dichotomized at the median into low- and high-expression groups. Odds ratios were estimated using logistic regression with two-sided Wald tests, interaction effects were evaluated using two-sided likelihood ratio tests, and no adjustment for multiple comparisons was applied. Source data are provided as a Source Data file.

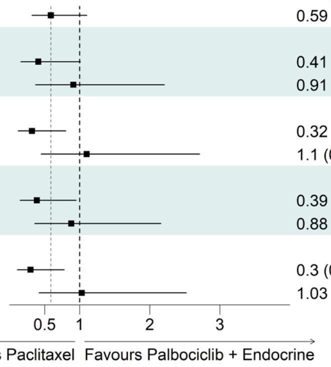

Biomarker	Paclitaxel	Palbociclib + Endocrine	OR (95% CI)	p value	p interaction
All patients (ITT population)	46/80 (57.5%)	37/83 (44.6%)	0.59 (0.32-1.1)	0.1	
**ESR1 module**					
Low	30/43 (69.8%)	19/39 (48.7%)	0.41 (0.16-1.01)	0.054	0.219
High	16/37 (43.2%)	18/44 (40.9%)	0.91 (0.37-2.21)	0.832	
**21-gene RS ER module**					
Low	31/42 (73.8%)	19/40 (47.5%)	0.32 (0.12-0.8)	0.016	0.057
High	15/38 (39.5%)	18/43 (41.9%)	1.1 (0.45-2.71)	0.827	
**SET ER,PR**					
Low	30/41 (73.2%)	21/41 (51.2%)	0.39 (0.15-0.95)	0.043	0.202
High	16/39 (41%)	16/42 (38.1%)	0.88 (0.36-2.16)	0.788	
**Breast Cancer 360 : ER signaling**					
Low	31/40 (77.5%)	21/41 (51.2%)	0.3 (0.11-0.78)	0.016	0.068
High	15/40 (37.5%)	16/42 (38.1%)	1.03 (0.42-2.52)	0.956	

Supplementary Figure 5: Forest plot for the primary endpoint ORR_{12} in PREDIX LumB according to genes and deconvoluted immune cell populations related to antigen presentation. Subgroups were dichotomized at the median into low- and high-expression groups. The deconvolution method used is indicated before each cell type. Odds ratios were estimated using logistic regression with two-sided Wald tests, interaction effects were evaluated using two-sided likelihood ratio tests, and no adjustment for multiple comparisons was applied. Source data are provided as a Source Data file.

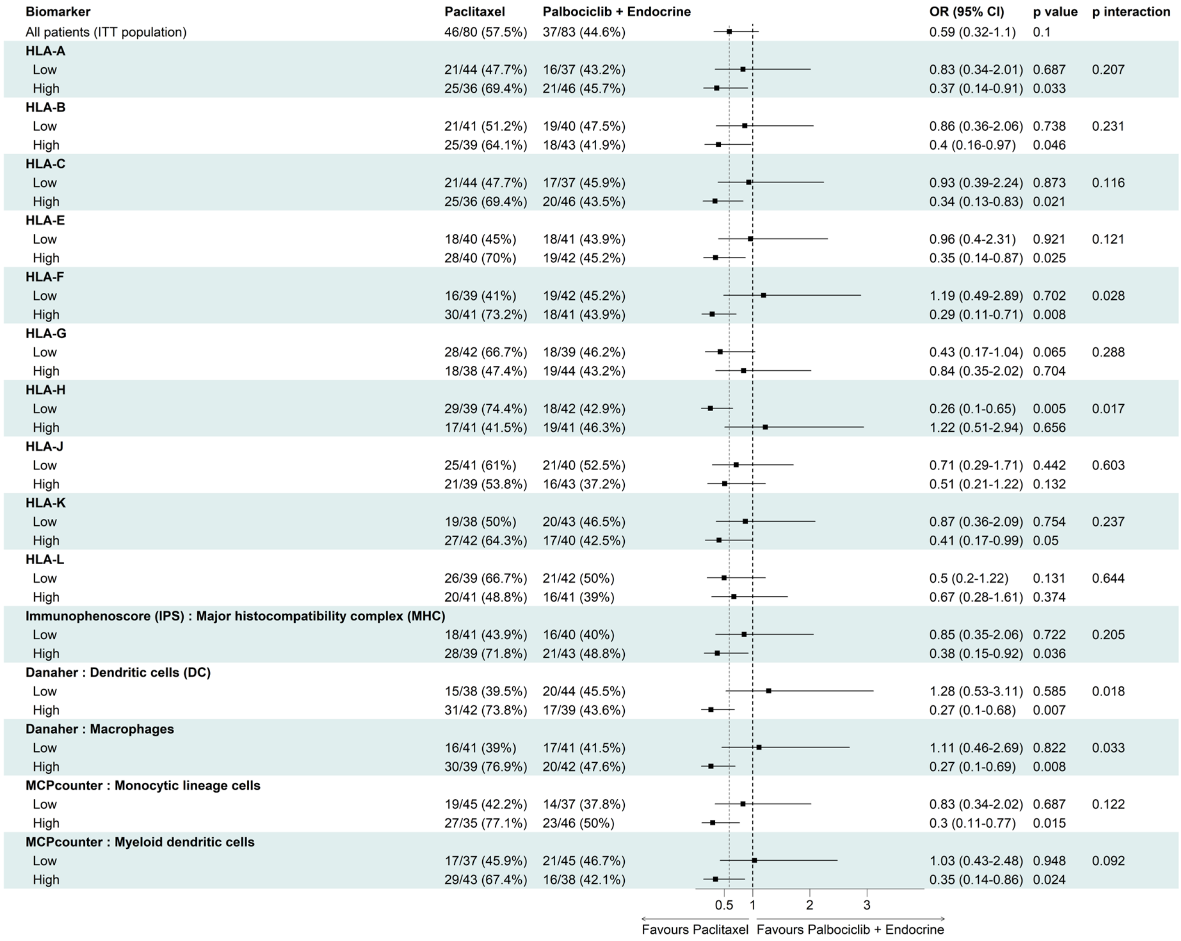

Supplementary Figure 6: Forest plot for the primary endpoint ORR_{12} in PREDIX LumB according to genes and deconvoluted immune cell populations related to T cell activity. Subgroups were dichotomized at the median into low- and high-expression groups. The deconvolution method used is indicated before each cell type. Odds ratios were estimated using logistic regression with two-sided Wald tests, interaction effects were evaluated using two-sided likelihood ratio tests, and no adjustment for multiple comparisons was applied. Source data are provided as a Source Data file.

Biomarker	Paclitaxel	Palbociclib + Endocrine	OR (95% CI)	p value	p interaction
All patients (ITT population)	46/80 (57.5%)	37/83 (44.6%)	0.59 (0.32-1.1)	0.1	
**CD274**					
Low	18/38 (47.4%)	18/43 (41.9%)	0.8 (0.33-1.93)	0.619	0.372
High	28/42 (66.7%)	19/40 (47.5%)	0.45 (0.18-1.09)	0.082	
**Immunophenoscore (IPS) : Effector cells**					
Low	24/37 (64.9%)	21/44 (47.7%)	0.49 (0.2-1.2)	0.124	0.645
High	22/43 (51.2%)	16/39 (41%)	0.66 (0.27-1.59)	0.359	
**Danaher : CD8 T cells**					
Low	20/37 (54.1%)	20/45 (44.4%)	0.68 (0.28-1.63)	0.387	0.693
High	26/43 (60.5%)	17/38 (44.7%)	0.53 (0.22-1.27)	0.159	
**Danaher : Cytotoxic T cells**					
Low	14/38 (36.8%)	18/44 (40.9%)	1.19 (0.49-2.92)	0.707	0.035
High	32/42 (76.2%)	19/39 (48.7%)	0.3 (0.11-0.75)	0.012	
**Danaher : Exhausted CD8+ T cells**					
Low	18/38 (47.4%)	18/44 (40.9%)	0.77 (0.32-1.85)	0.557	0.451
High	28/42 (66.7%)	19/39 (48.7%)	0.48 (0.19-1.16)	0.104	
**Danaher : T cells**					
Low	18/38 (47.4%)	19/44 (43.2%)	0.84 (0.35-2.02)	0.704	0.288
High	28/42 (66.7%)	18/39 (46.2%)	0.43 (0.17-1.04)	0.065	
**Danaher : T helper cells**					
Low	16/40 (40%)	20/42 (47.6%)	1.36 (0.57-3.3)	0.488	0.007
High	30/40 (75%)	17/41 (41.5%)	0.24 (0.09-0.59)	0.003	
**MCPcounter : CD8 T cells**					
Low	21/40 (52.5%)	18/42 (42.9%)	0.68 (0.28-1.62)	0.383	0.671
High	25/40 (62.5%)	19/41 (46.3%)	0.52 (0.21-1.25)	0.146	
**MCPcounter : Cytotoxic T cells**					
Low	14/36 (38.9%)	19/46 (41.3%)	1.11 (0.45-2.72)	0.825	0.081
High	32/44 (72.7%)	18/37 (48.6%)	0.36 (0.14-0.88)	0.028	
**MCPcounter : T cells**					
Low	18/38 (47.4%)	18/44 (40.9%)	0.77 (0.32-1.85)	0.557	0.451
High	28/42 (66.7%)	19/39 (48.7%)	0.48 (0.19-1.16)	0.104	

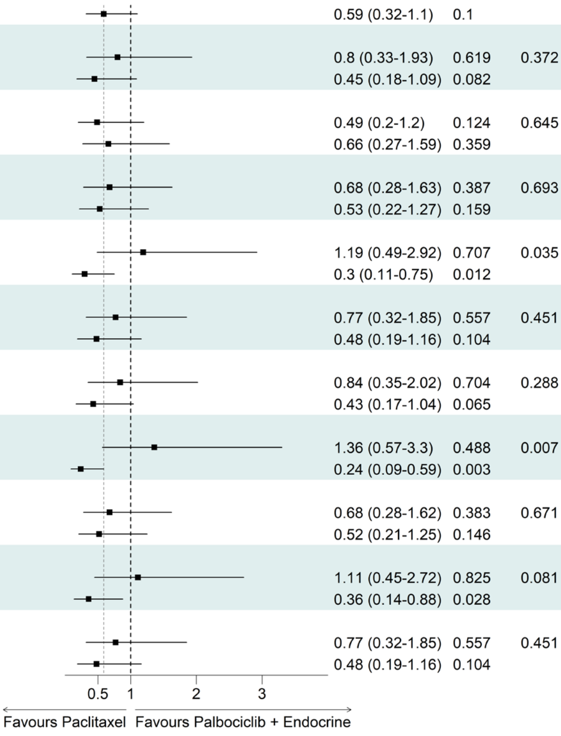

Supplementary Figure 7: Forest plot for the primary endpoint ORR_{12} in PREDIX LumB according to genes and deconvoluted immune cell populations related to humoral immune activity. Subgroups were dichotomized at the median into low- and high-expression groups. The deconvolution method used is indicated before each cell type. Immune aggregates were assessed histomorphologically in H&E slides. The immunoglobulin B-cell signature (IGG) comprises 14 genes involved in multiple immune processes, including T- and B-lymphocyte progenitor maturation (*IL2RG*), activation and survival of $CD4^{+}$ and B lymphocytes (*CD27*, *TNFRSF17*, *PIM2*), B-cell differentiation within germinal centers (*POU2AF1*), immunoglobulin synthesis (*CD79a*, *JCHAIN*, *IGKC*, *IGL*, *IGLV3-25*), chemotaxis (*CXCL8*, *NTN3*), and regulation of B-, T-, and NK-cell activity (*LAX1*, *HLA-C*). The 9-gene TLS signature is composed of genes associated with B cells and other immune cells activity (*CD79B*, *CD1D*, *CCR6*, *LAT*, *SKAP1*, *CETP*, *EIFIAY*, *RBP5*, and *PIGDS*) and was developed to reflect the presence of tertiary lymphoid structures (TLS) in the tumor microenvironment. The 12-chemokine signature is composed of chemokine genes (*CCL2, CCL3, CCL4, CCL5, CCL8, CCL18, CCL19, CCL21, CXCL9, CXCL10, CXCL11*, and *CXCL13*) that mediate immune-cell recruitment and TLS formation. Odds ratios were estimated using logistic regression with two-sided Wald tests, interaction effects were evaluated using two-sided likelihood ratio tests, and no adjustment for multiple comparisons was applied. Source data are provided as a Source Data file.

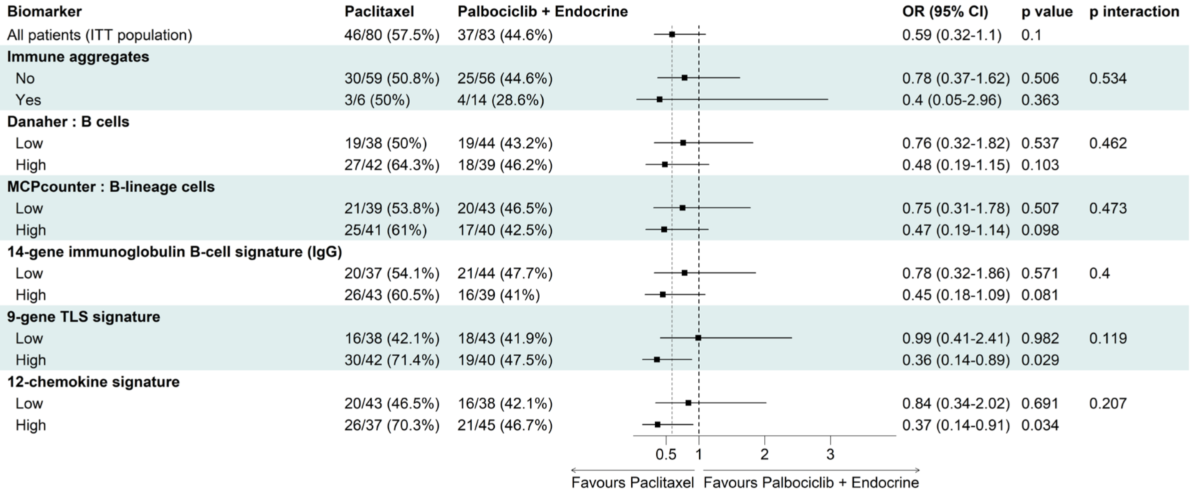

Supplementary Figure 8: Forest plot for the primary endpoint ORR_{12} in PREDIX LumB according to the various immune components derived in silico from the publicly available gene sets of the Breast Cancer 360™ (BC360) gene panel. Subgroups were dichotomized at the median into low- and high-expression groups. Odds ratios were estimated using logistic regression with two-sided Wald tests, interaction effects were evaluated using two-sided likelihood ratio tests, and no adjustment for multiple comparisons was applied. Source data are provided as a Source Data file.

Biomarker	Paclitaxel	Palbociclib + Endocrine	OR (95% CI)	p value	p interaction
All patients (ITT population)	46/80 (57.5%)	37/83 (44.6%)	0.59 (0.32-1.1)	0.1	
**Breast Cancer 360 : Antigen presentation**					
Low	22/44 (50%)	15/37 (40.5%)	0.68 (0.28-1.64)	0.395	0.537
High	24/36 (66.7%)	22/46 (47.8%)	0.46 (0.18-1.12)	0.09	
**Breast Cancer 360 : Cytokine and chemokine signaling**					
Low	18/41 (43.9%)	17/40 (42.5%)	0.94 (0.39-2.28)	0.899	0.115
High	28/39 (71.8%)	20/43 (46.5%)	0.34 (0.13-0.84)	0.022	
**Breast Cancer 360 : JAK-STAT**					
Low	18/38 (47.4%)	18/43 (41.9%)	0.8 (0.33-1.93)	0.619	0.372
High	28/42 (66.7%)	19/40 (47.5%)	0.45 (0.18-1.09)	0.082	
**Breast Cancer 360 : Immune infiltration**					
Low	20/42 (47.6%)	17/39 (43.6%)	0.85 (0.35-2.04)	0.716	0.216
High	26/38 (68.4%)	20/44 (45.5%)	0.38 (0.15-0.94)	0.039	

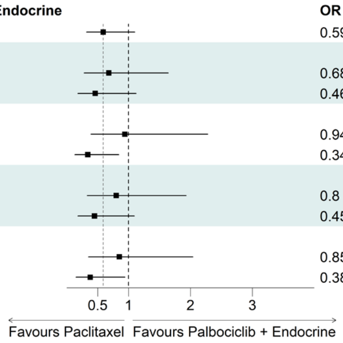

Supplementary Figure 9: Hallmark MSigDB gene set enrichment analysis (GSEA) barplots comparing patients with residual cancer burden (RCB) 0/1 vs. 2/3 in PREDIX LumB. The RCB index classifies patients into four categories according to the extent of residual disease after neoadjuvant therapy, as determined by the pathologist. Normalized enrichment scores (NES) are shown for significantly enriched pathways (p-adj < 0.05); upregulated pathways are depicted in green and downregulated in dark orange. Source data are provided as a Source Data file.

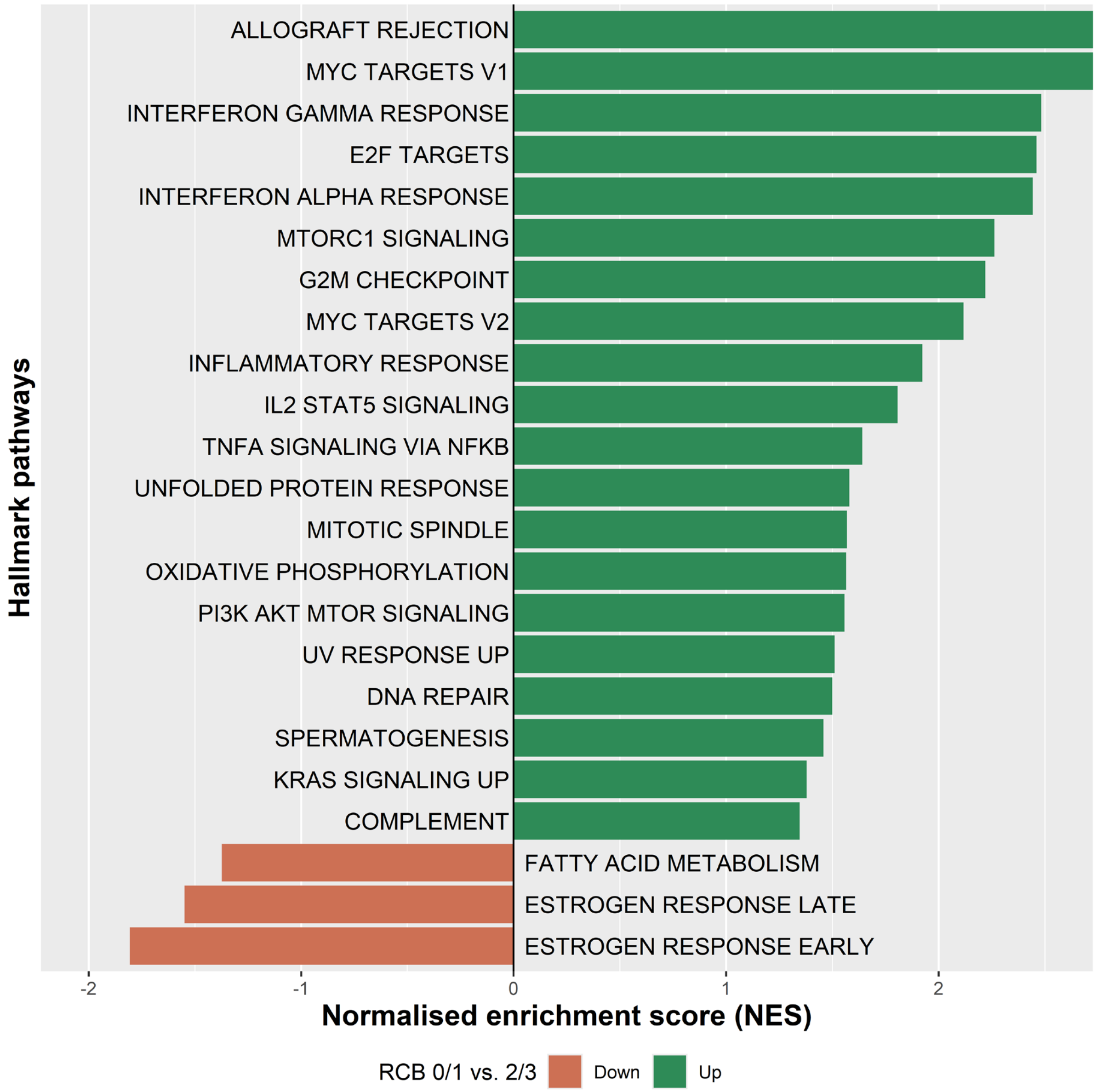

Supplementary Figure 10: Statistically significant correlations between clinicopathologic features and CDKPredX groups in PREDIX LumB (A) and SCAN-B (B) trials, assessed using Pearson's chi-squared test. Source data are provided as a Source Data file.

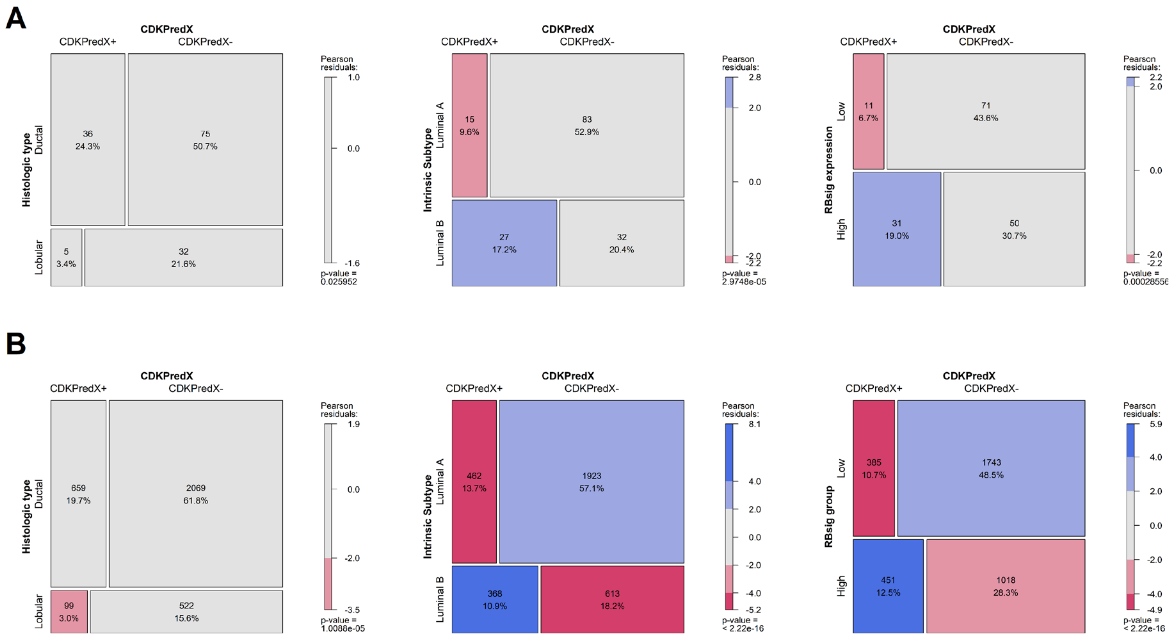

Supplementary Figure 11: Associations of *RB1* copy number alteration (CNA) status in PREDIX LumB with CDKPredX groups and separately with RBsig (Rb loss-of-function) high-/low-expression groups (A), assessed using Pearson's chi-squared test. *RB1* exhibited shallow deletions in 50 patients (30.1%) and deep deletions in 5 patients (3.0%). Copy number alterations were identified using GISTIC2.0 and classified as deep deletion, shallow deletion, neutral, gain, or amplification. Scatter plot and Spearman correlation between RBsig and E2F signature expression levels (B). For the E2F signature, the mean expression of 200 genes encoding cell cycle–related targets of E2F transcription factors, as defined in the MSigDB, was calculated for each sample. Source data are provided as a Source Data file.

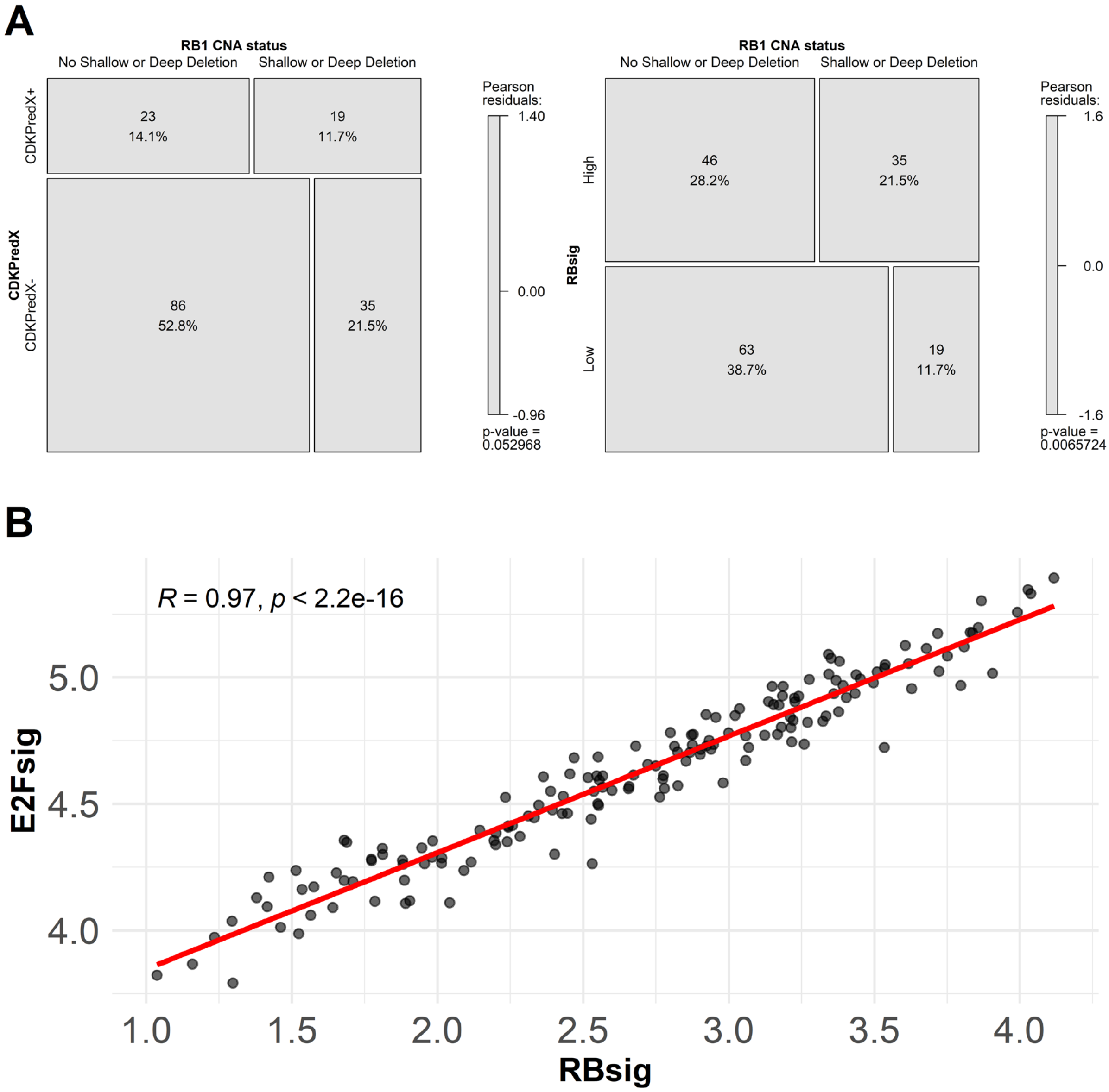

Supplementary Figure 12: Comparison of unscaled continuous 21-gene recurrence score distributions, computed using a research-based implementation, between CDKPredX-defined subgroups in PREDIX LumB using the Wilcoxon rank-sum test. Source data are provided as a Source Data file.

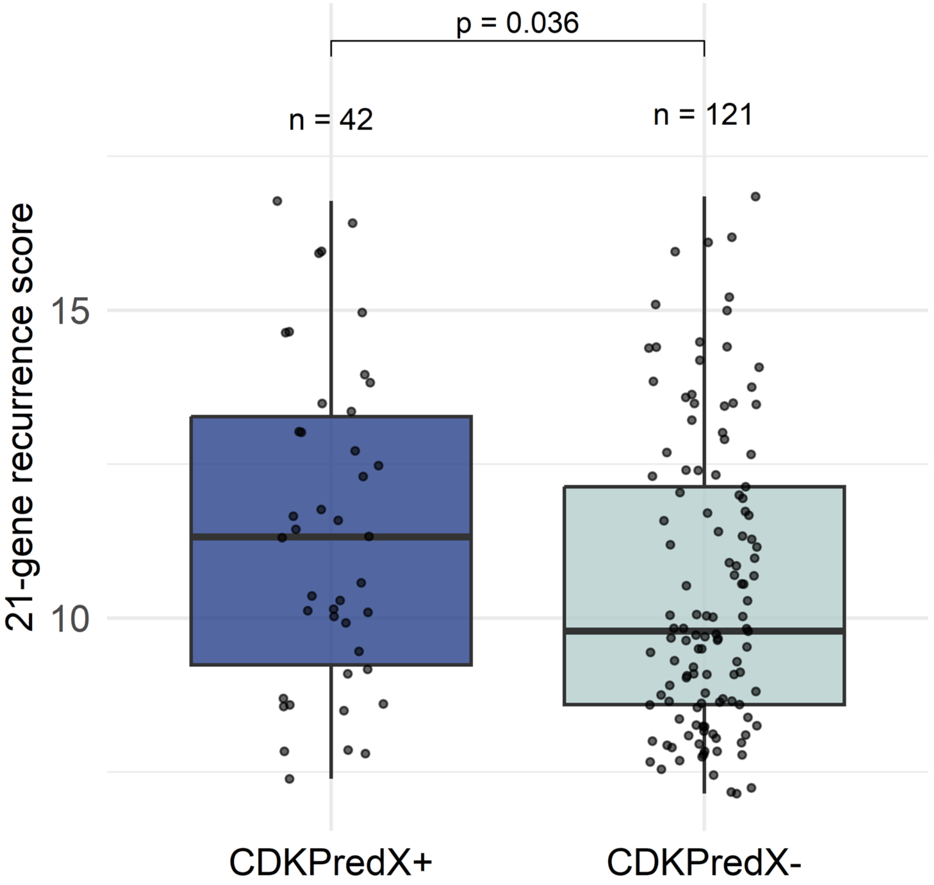

Supplementary Figure 13: Forest plots for the primary endpoint of ORR_{12} in PREDIX LumB according to the CDKPredX predictor, in the entire population and separately for premenopausal and postmenopausal patients. Odds ratios were estimated using logistic regression with two-sided Wald tests, interaction effects were evaluated using two-sided likelihood ratio tests, and no adjustment for multiple comparisons was applied. Source data are provided as a Source Data file.

Biomarker	Chemotherapy	CDK4/6i + Endocrine	OR (95% CI)	p value	p interaction
**All patients**	**46/80 (57.5%)**	**37/83 (44.6%)**	**0.59 (0.32-1.1)**	**0.1**	
**CDKPredX**					
CDKPredX+	6/19 (31.6%)	11/23 (47.8%)	1.99 (0.57-7.38)	0.288	0.03
CDKPredX-	40/61 (65.6%)	26/60 (43.3%)	0.4 (0.19-0.83)	0.015	
**Premenopausal patients**	**24/34 (70.6%)**	**17/33 (51.5%)**	**0.44 (0.16-1.19)**	**0.112**	
**CDKPredX**					
CDKPredX+	4/6 (66.7%)	6/7 (85.7%)	3 (0.21-78.76)	0.427	0.11
CDKPredX-	20/28 (71.4%)	11/26 (42.3%)	0.29 (0.09-0.89)	0.033	
**Postmenopausal patients**	**22/44 (50%)**	**18/45 (40%)**	**0.67 (0.29-1.54)**	**0.344**	
**CDKPredX**					
CDKPredX+	2/12 (16.7%)	5/16 (31.2%)	2.27 (0.39-18.47)	0.384	0.141
CDKPredX-	20/32 (62.5%)	13/29 (44.8%)	0.49 (0.17-1.34)	0.169	

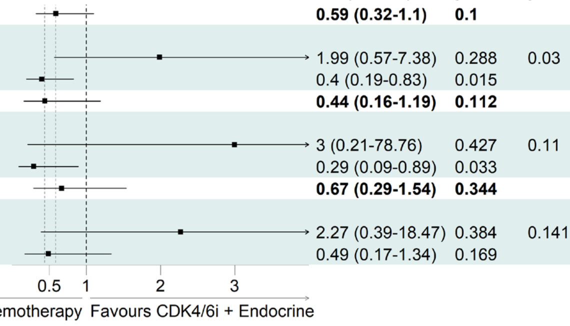

Supplementary Figure 14: Barplots showing ORR_{24} (A), RCB (B) and pCR (C) rates in PREDIX LumB trial according to CDKPredX groups. Group differences were assessed using two-sided Fisher's exact tests. Source data are provided as a Source Data file.

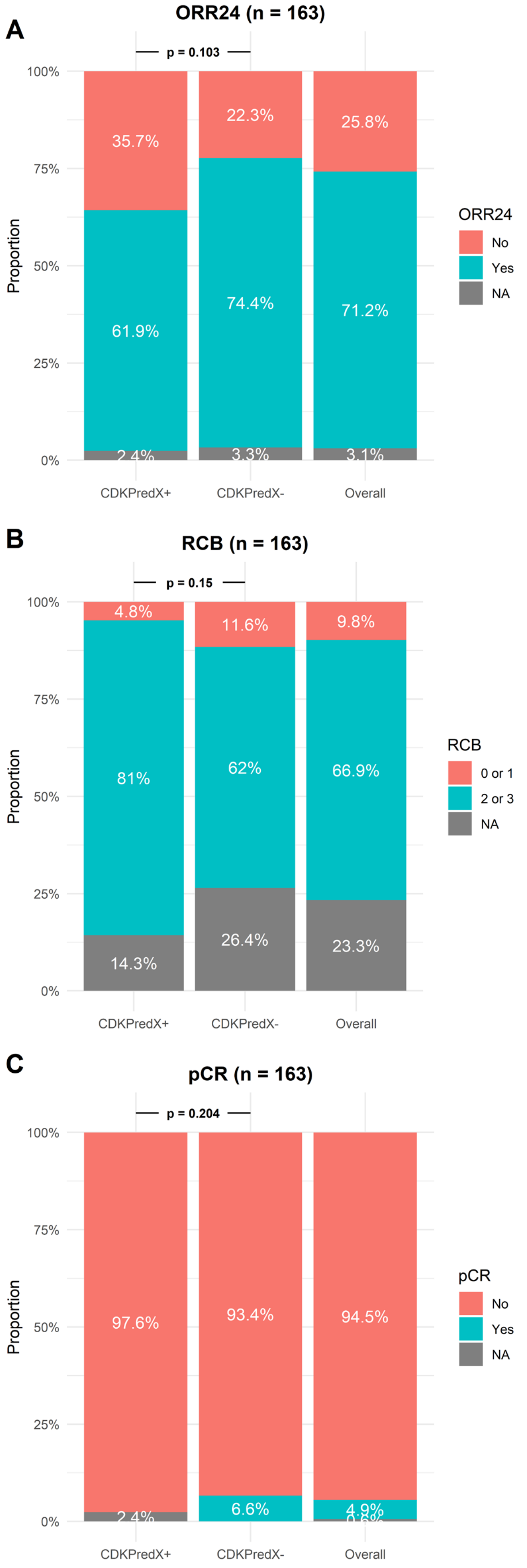

Supplementary Figure 15: Kaplan-Meier curves of event-free survival (EFS) (A), relapse-free survival (RFS) (B) and overall survival (OS) (C), for CDKPredX groups, after a median follow-up of 4.52 years.

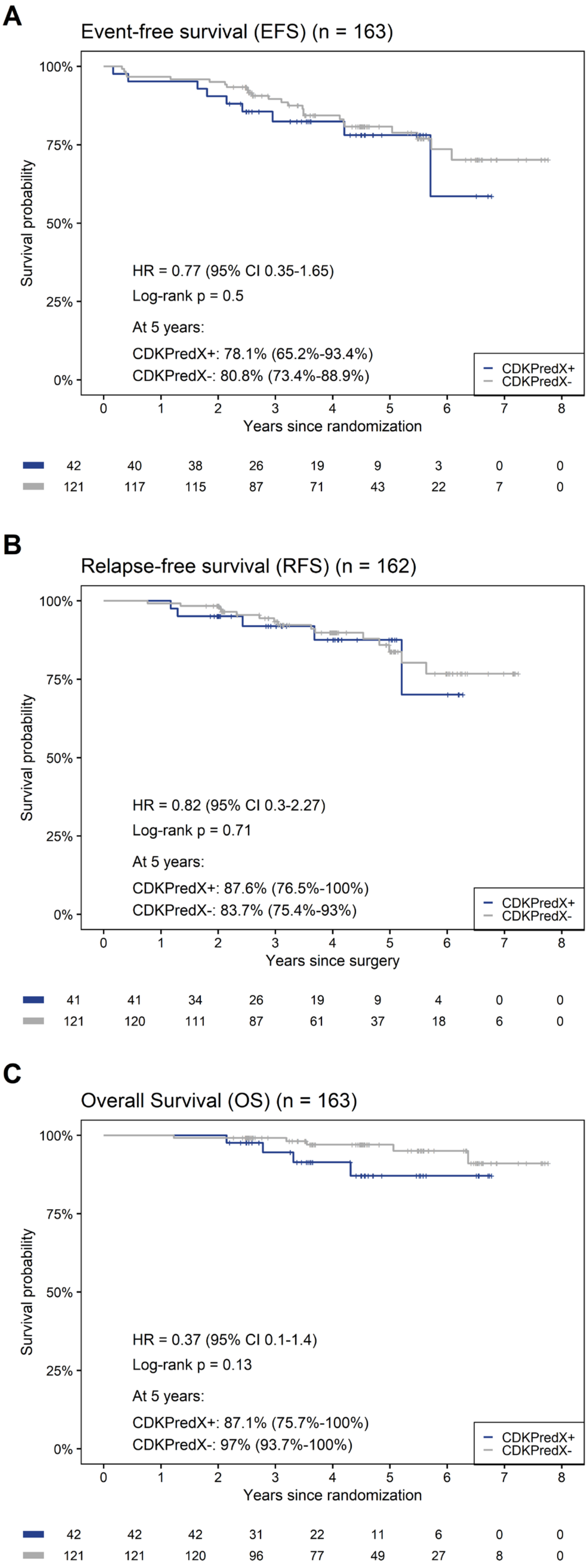

Supplementary Figure 16: Barplots showing ORR_{24}, RCB and pCR rates, according to CDKPredX groups (A), and forest plot for the primary endpoint ORR_{12} according to PET/CT SUVmax (continuous variable) (B), in patients with tumors of lobular histology from PREDIX LumB trial. Group differences were assessed using two-sided Fisher's exact tests. Odds ratios were estimated using logistic regression with two-sided Wald tests, interaction effect was evaluated using two-sided likelihood ratio test. Source data are provided as a Source Data file.

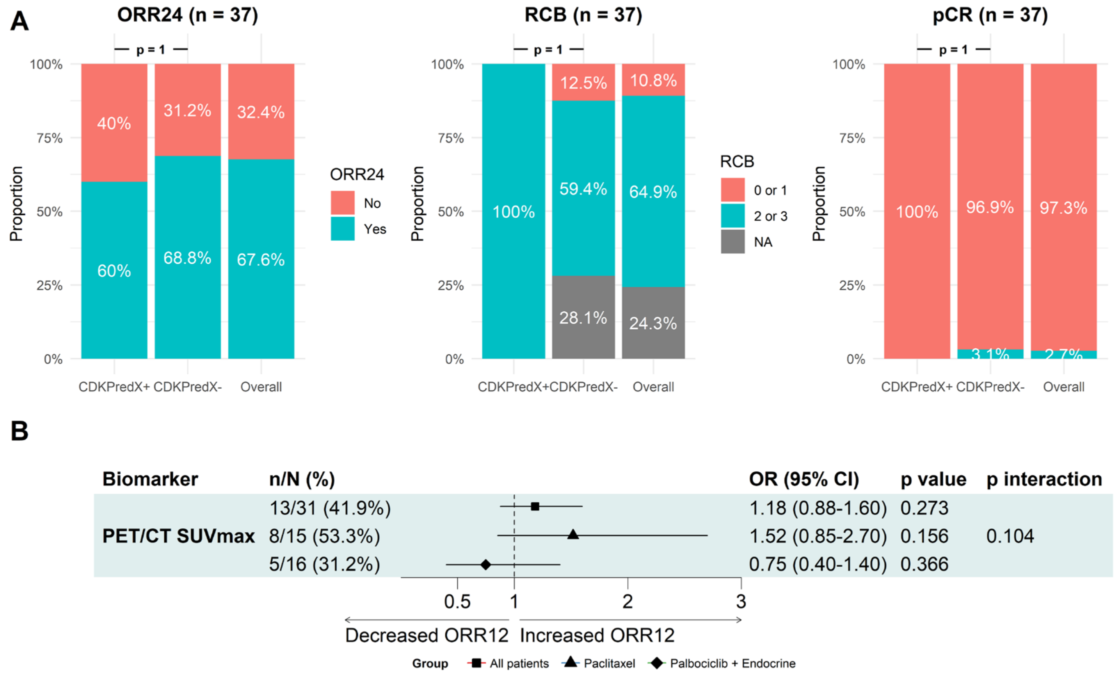

Supplementary Figure 17: Association of pCR with CDKPredX groups in patients with ER+ /HER2- tumors from the I-SPY 2 trial: forest plot for patients who received neoadjuvant chemotherapy with or without immunotherapy (checkpoint inhibition) (A), and contingency table for patients who received chemotherapy plus checkpoint inhibition (B). Odds ratio was estimated using logistic regression with two-sided Wald tests, interaction effect was evaluated using two-sided likelihood ratio test. Source data are provided as a Source Data file.

A

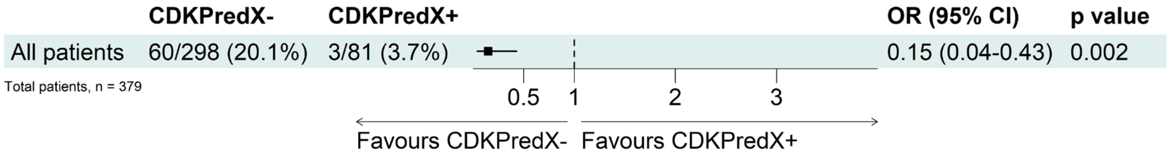

B

	pCR = No	pCR = Yes
**CDKPredX-**	18	12
**CDKPredX+**	10	0

Supplementary Figure 18: Association of CDKPredX groups with distant recurrence-free interval (DRFi) in patients with ER+/HER2- tumors from the SCAN-B cohort: Kaplan–Meier curves comparing CDKPredX groups (A), and Kaplan–Meier curves for the CDKPredX+ group comparing chemotherapy treatment allocation, adjusted for age, size, lymph node status, and grade, using inverse probability treatment weighting (IPTW) (B).

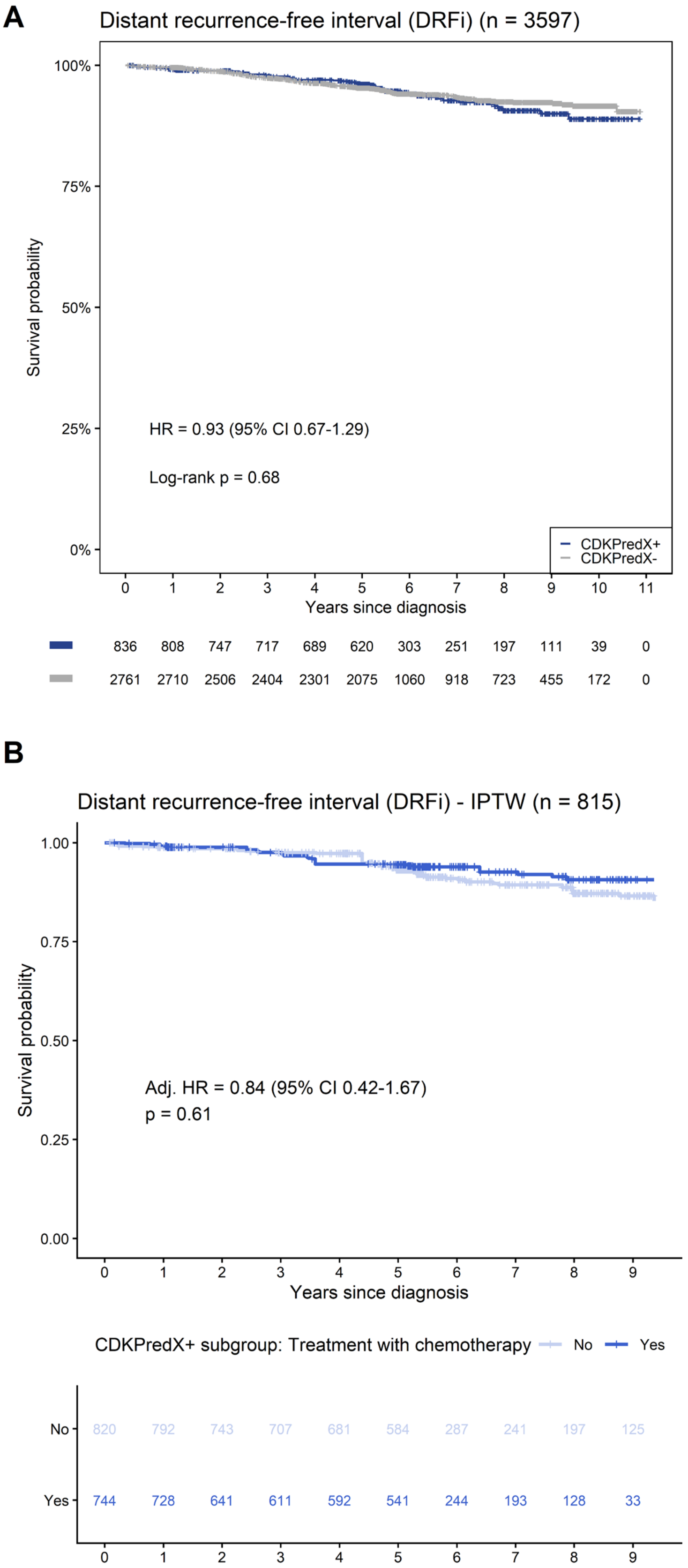

Supplementary Figure 19: Mean differences (MD) and 95% confidence intervals (CI) at baseline between treatment groups according to EORTC QLQ-C30 (A) and EORTC QLQ-BR23 questionnaires (B). Source data are provided as a Source Data file.

A) EORTC QLQ-C30 at Baseline

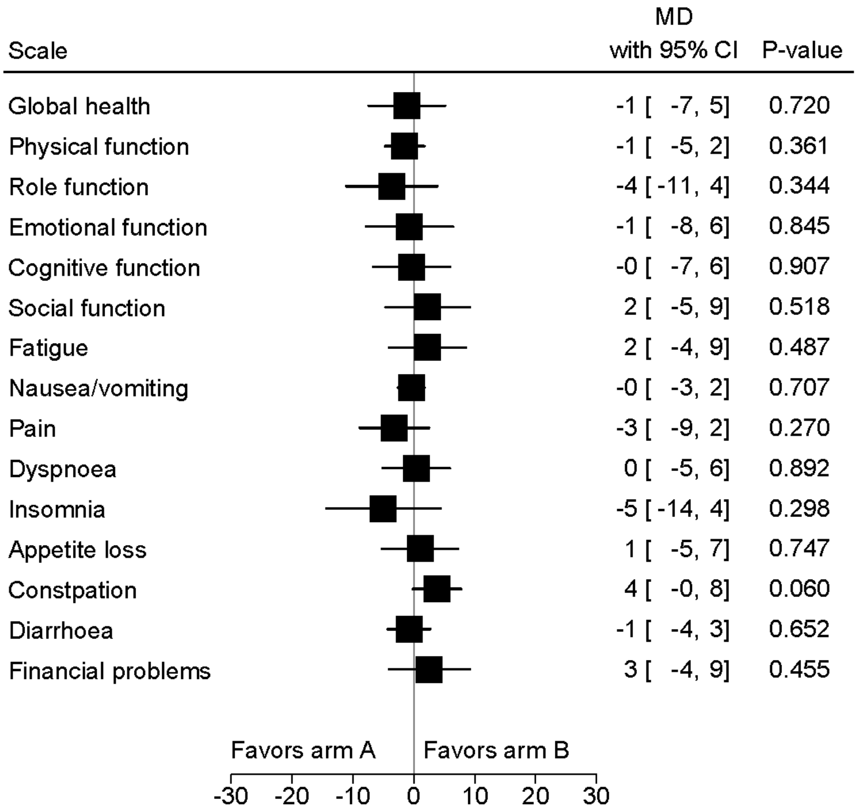

B) EORTC QLQ-BR23 at Baseline

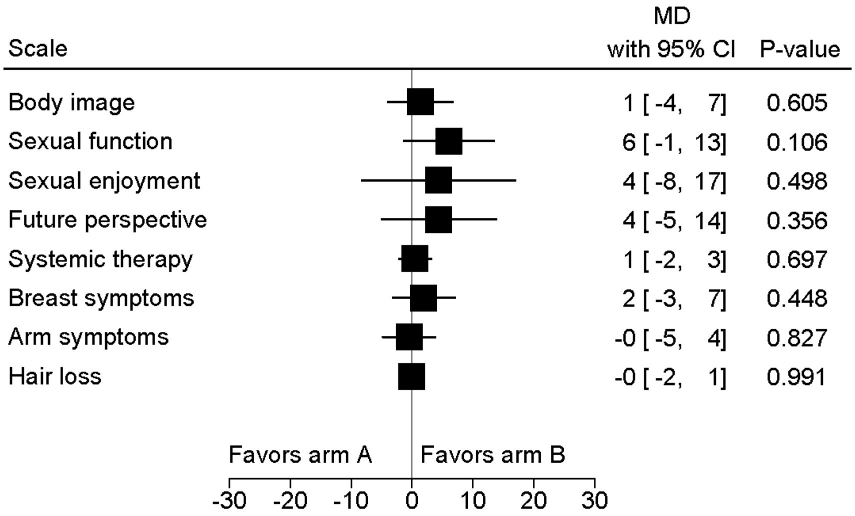

Supplementary Figure 20: Mean differences (MD) and 95% confidence intervals (CI) at 12 weeks prior to treatment switch between treatment groups according to EORTC QLQ-C30 (A) and EORTC QLQ-BR23 questionnaires (B). Source data are provided as a Source Data file.

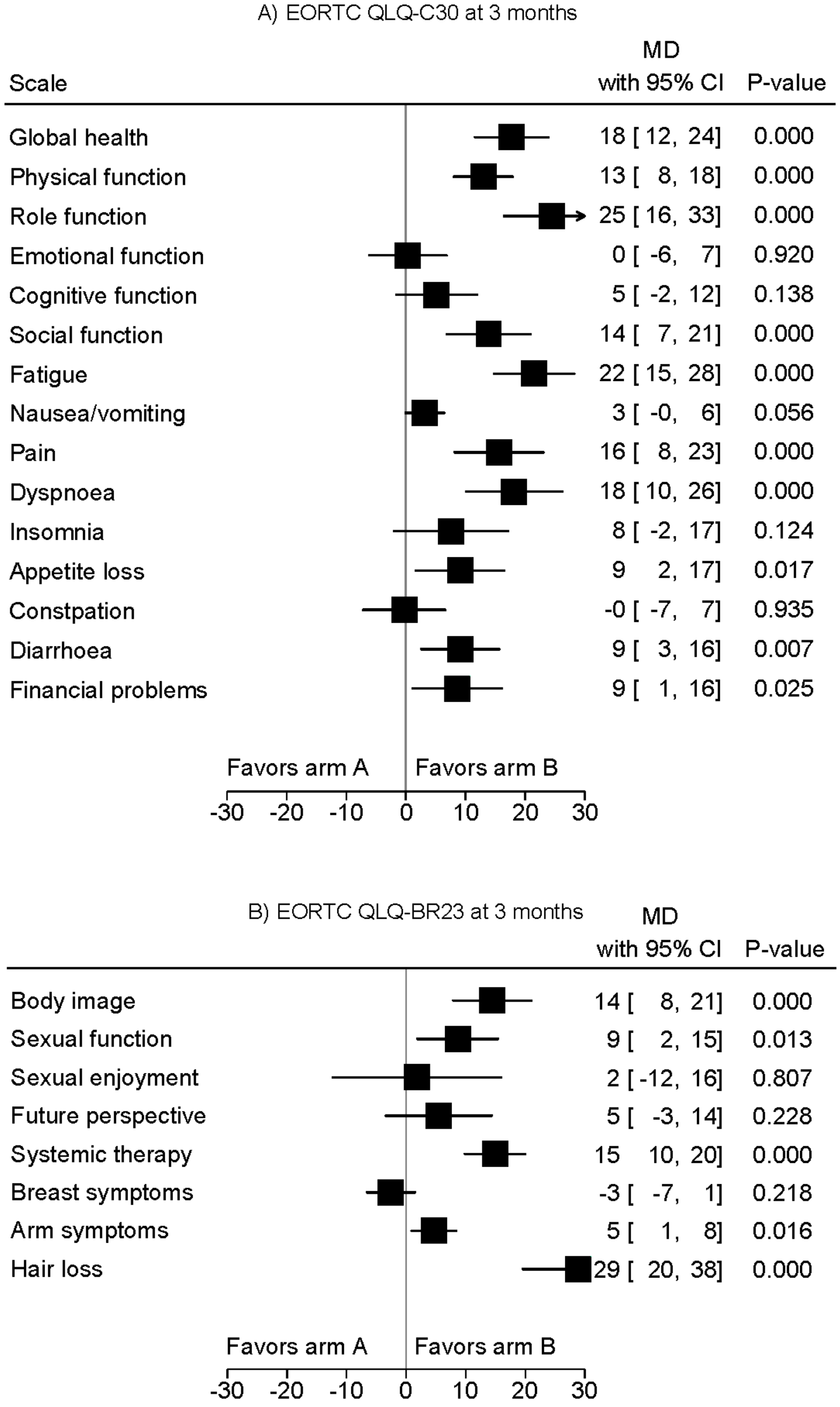

Supplementary Figure 21: Mean differences (MD) and 95% confidence intervals (CI) at end of neoadjuvant treatment switch between treatment groups according to EORTC QLQ-C30 (A) and EORTC QLQ-BR23 questionnaires (B). Source data are provided as a Source Data file.

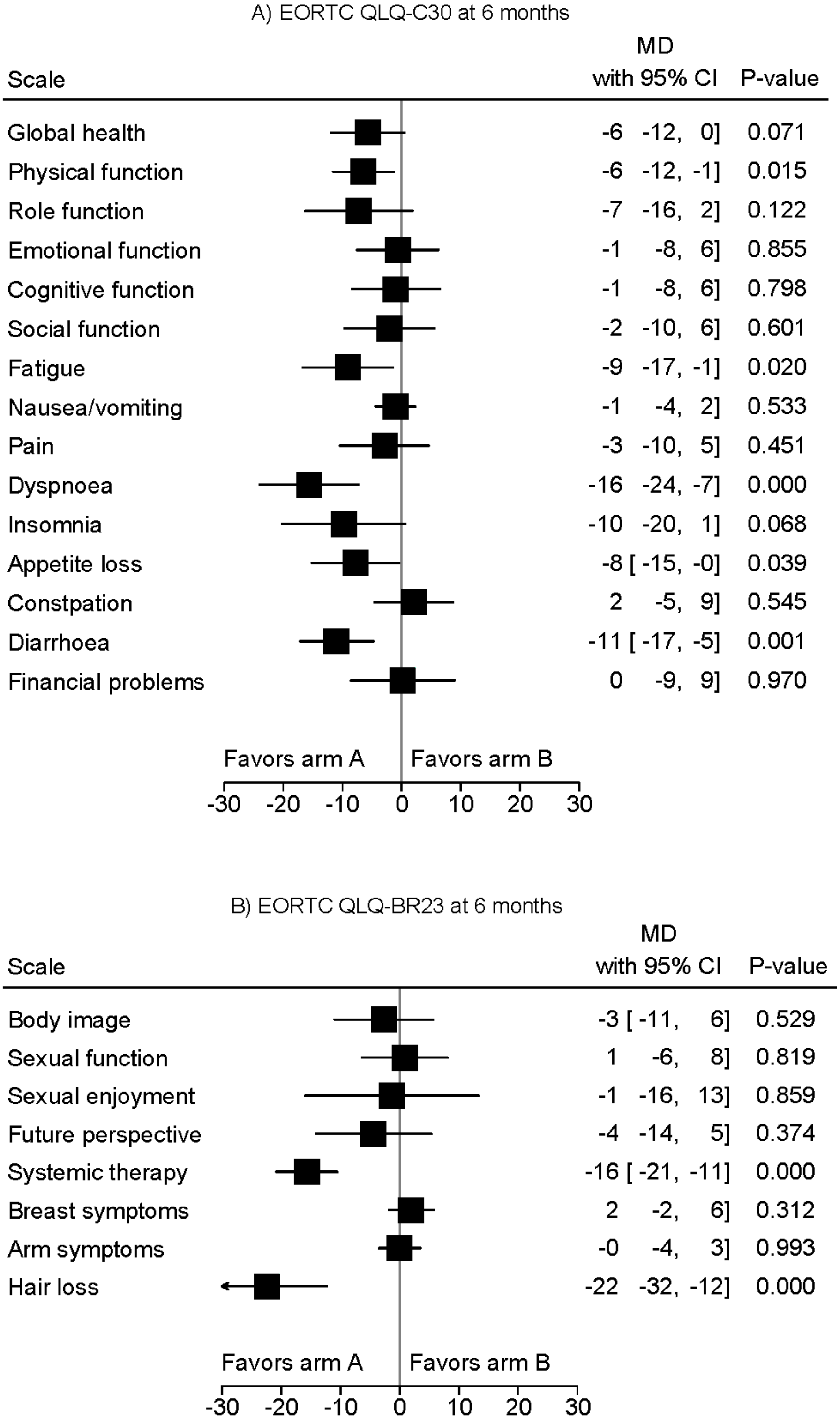

Supplementary Figure 22: HRQoL at 18 months according to EORTC QLQ-C30 questionnaire of the entire study population (observed) compared to population-based normative data (expected). Source data are provided as a Source Data file.

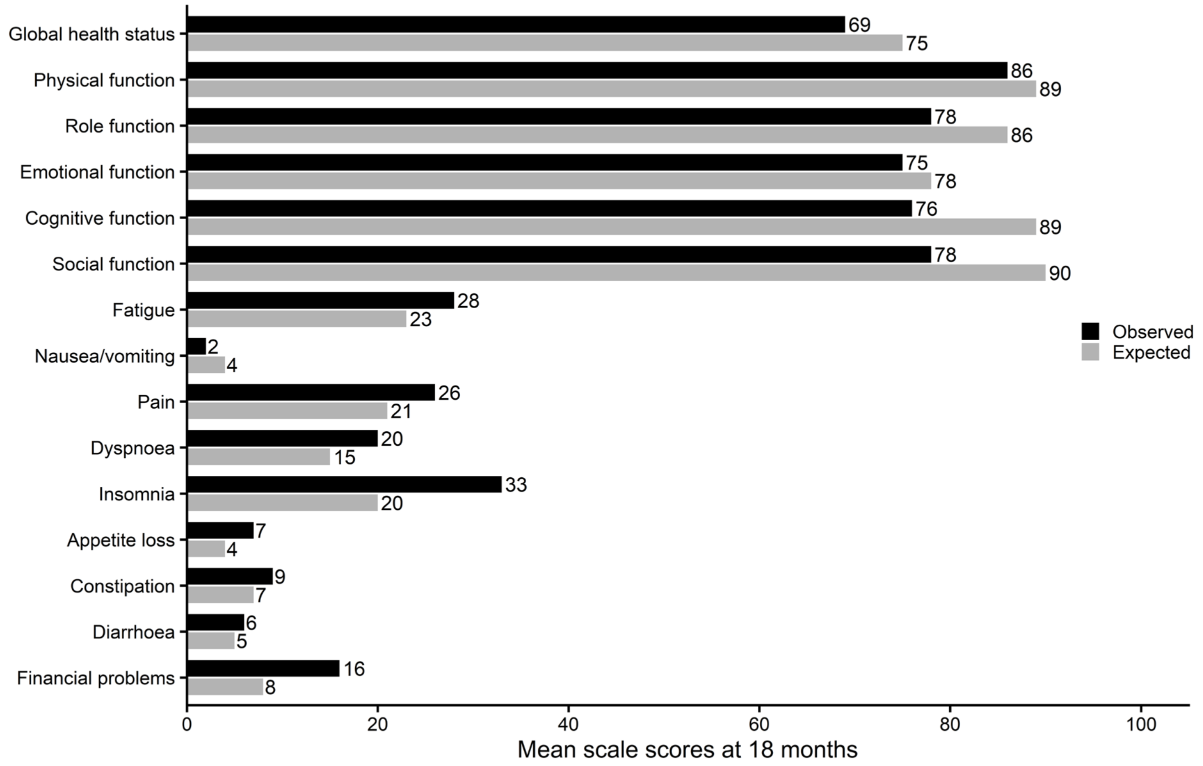

Supplementary Figure 23: Representative histomorphological H&E digital images of immune cell aggregation derived from baseline biopsies of PREDIX LumB study. For all images scale bar is 50 um. Immune aggregation inside the invasive cancer area (A), (B). Immune aggregation outside the invasive cancer area (near a ductal carcinoma *in situ*) (C). A tumor without any immune aggregation (D).

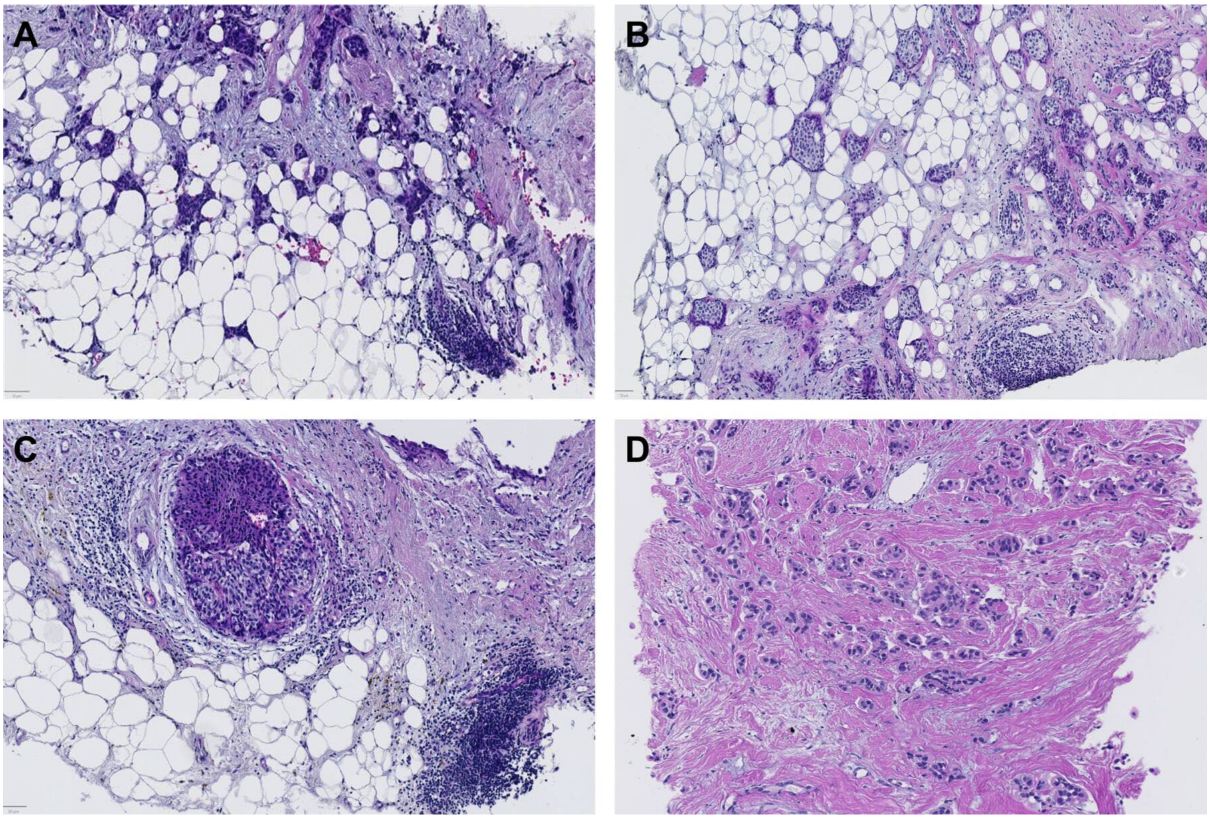

Supplementary Table 1: Clinicopathologic and molecular characteristics of the CDKPredX subgroups

	CDKPredX+		CDKPredX-	
	**Arm A n = 19**	**Arm B n = 23**	**Arm A n = 61**	**Arm B n = 60**
**ORR12**				
1	6 (31.6%)	11 (47.8%)	40 (65.6%)	26 (43.3%)
0	13 (68.4%)	12 (52.2%)	21 (34.4%)	34 (56.7%)
**Tumor size**				
< 2 cm	3 (15.8%)	7 (30.4%)	10 (16.4%)	8 (13.3%)
2 – 5cm	14 (73.7%)	12 (52.2%)	36 (59%)	34 (56.7%)
> 5 cm	2 (10.5%)	4 (17.4%)	15 (24.6%)	18 (30%)
**Lymph nodes**				
Positive	10 (52.6%)	13 (56.5%)	40 (65.6%)	44 (73.3%)
Negative	9 (47.4%)	10 (43.5%)	21 (34.4%)	16 (26.7%)
**Histologic type**				
Ductal	18 (94.7%)	18 (78.3%)	38 (62.3%)	37 (61.7%)
Lobular	1 (5.3%)	4 (17.4%)	16 (26.2%)	16 (26.7%)
Unknown	0 (0%)	1 (4.3%)	7 (11.5%)	7 (11.7%)
**Histologic grade**				
I/II	17 (89.5%)	20 (87%)	45 (73.8%)	51 (85%)
III	2 (10.5%)	2 (8.7%)	16 (26.2%)	9 (15%)
Unknown	0 (0%)	1 (4.3%)	0 (0%)	0 (0%)
**Ki67 %**				
Median (interquartile range)	28 (25.5 – 31)	25 (23 – 33)	30 (20 – 40)	24.5 (20 – 33)
**TILs**				
> 1	5 (26.3%)	8 (34.8%)	16 (26.2%)	21 (35%)
<= 1	13 (68.4%)	15 (65.2%)	29 (47.5%)	25 (41.7%)
Unknown	1 (5.3%)	0 (0%)	16 (26.2%)	14 (23.3%)
**Intrinsic Subtype**				
Luminal A	7 (36.8%)	8 (34.8%)	42 (68.9%)	41 (68.3%)
Luminal B	12 (63.2%)	15 (65.2%)	15 (24.6%)	17 (28.3%)
Non-luminal	0 (0%)	0 (0%)	4 (6.6%)	2 (3.3%)
**RBsig expression**				
High	14 (73.7%)	17 (73.9%)	22 (36.1%)	28 (46.7%)
Low	5 (26.3%)	6 (26.1%)	39 (63.9%)	32 (53.3%)
**PIK3CA mutations**				
No	12 (63.2%)	13 (56.5%)	42 (68.9%)	36 (60%)
Yes	7 (36.8%)	10 (43.5%)	19 (31.1%)	24 (40%)
**CDH1 mutations**				
No	18 (94.7%)	20 (87%)	49 (80.3%)	47 (78.3%)
Yes	1 (5.3%)	3 (13%)	12 (19.7%)	13 (21.7%)
**APOBEC Enriched**				
No	15 (78.9%)	18 (78.3%)	40 (65.6%)	41 (68.3%)
Yes	4 (21.1%)	5 (21.7%)	17 (27.9%)	13 (21.7%)
Unknown	0 (0%)	0 (0%)	4 (6.6%)	6 (10%)
**TMB**				
< 1 mut/Mb	17 (89.5%)	18 (78.3%)	43 (70.5%)	34 (56.7%)
>= 1 mut/Mb	2 (10.5%)	5 (21.7%)	15 (24.6%)	20 (33.3%)
Unknown	0 (0%)	0 (0%)	3 (4.9%)	6 (10%)
**Integrative Cluster**				
IntClusts 3, 4, 7, 8 (low-risk)	11 (57.9%)	15 (65.2%)	40 (65.6%)	43 (71.7%)
IntClusts 1, 2, 6, 9 (high-risk)	8 (42.1%)	6 (26.1%)	18 (29.5%)	14 (23.3%)
IntClusts 5, 10 (non-luminal)	0 (0%)	2 (8.7%)	3 (4.9%)	3 (5%)

Section/topic	No	CONSORT 2025 checklist item description	Reported on page no.
**Title and abstract**			
Title and structured abstract	1a	Identification as a randomised trial	1
	1b	Structured summary of the trial design, methods, results, and conclusions	4
**Open science**			
Trial registration	2	Name of trial registry, identifying number (with URL) and date of registration	18
Protocol and statistical analysis plan	3	Where the trial protocol and statistical analysis plan can be accessed	18
Data sharing	4	Where and how the individual de-identified participant data (including data dictionary), statistical code and any other materials can be accessed	N/A
Funding and conflicts of interest	5a	Sources of funding and other support (eg, supply of drugs), and role of funders in the design, conduct, analysis and reporting of the trial	40
	5b	Financial and other conflicts of interest of the manuscript authors	38-39
**Introduction**			
Background and rationale	6	Scientific background and rationale	5
Objectives	7	Specific objectives related to benefits and harms	6
**Methods**			
Patient and public involvement	8	Details of patient or public involvement in the design, conduct and reporting of the trial	N/A
Trial design	9	Description of trial design including type of trial (eg, parallel group, crossover), allocation ratio, and framework (eg, superiority, equivalence, non-inferiority, exploratory)	18
Changes to trial protocol	10	Important changes to the trial after it commenced including any outcomes or analyses that were not prespecified, with reason	N/A
Trial setting	11	Settings (eg, community, hospital) and locations (eg, countries, sites) where the trial was conducted	18
Eligibility criteria	12a	Eligibility criteria for participants	18
	12b	If applicable, eligibility criteria for sites and for individuals delivering the interventions (eg, surgeons, physiotherapists)	N/A
Intervention and comparator	13	Intervention and comparator with sufficient details to allow replication. If relevant, where additional materials describing the intervention and comparator (eg, intervention manual) can be accessed	19
Outcomes	14	Prespecified primary and secondary outcomes, including the specific measurement variable (eg, systolic blood pressure), analysis metric (eg, change from baseline, final value, time to event), method of aggregation (eg, median, proportion), and time point for each outcome	20-21
Harms	15	How harms were defined and assessed (eg, systematically, non-systematically)	N/A
Sample size	16a	How sample size was determined, including all assumptions supporting the sample size calculation	34
	16b	Explanation of any interim analyses and stopping guidelines	N/A
Randomisation:			
Sequence generation	17a	Who generated the random allocation sequence and the method used	19
	17b	Type of randomisation and details of any restriction (eg, stratification, blocking and block size)	19

			Reported on page no.
Allocation concealment mechanism	18	Mechanism used to implement the random allocation sequence (eg, central computer/telephone; sequentially numbered, opaque, sealed containers), describing any steps to conceal the sequence until interventions were assigned	19
Implementation	19	Whether the personnel who enrolled and those who assigned participants to the interventions had access to the random allocation sequence	8-9
Blinding	20a	Who was blinded after assignment to interventions (eg, participants, care providers, outcome assessors, data analysts)	8-9
	20b	If blinded, how blinding was achieved and description of the similarity of interventions	N/A
Statistical methods	21a	Statistical methods used to compare groups for primary and secondary outcomes, including harms	11-12
	21b	Definition of who is included in each analysis (eg, all randomised participants), and in which group	13
	21c	How missing data were handled in the analysis	N/A
	21d	Methods for any additional analyses (eg, subgroup and sensitivity analyses), distinguishing prespecified from post hoc	22-35
**Results**			
Participant flow, including flow diagram	22a	For each group, the numbers of participants who were randomly assigned, received intended intervention, and were analysed for the primary outcome	7
	22b	For each group, losses and exclusions after randomisation, together with reasons	7
Recruitment	23a	Dates defining the periods of recruitment and follow-up for outcomes of benefits and harms	7
	23b	If relevant, why the trial ended or was stopped	N/A
Intervention and comparator delivery	24a	Intervention and comparator as they were actually administered (eg, where appropriate, who delivered the intervention/comparator, how participants adhered, whether they were delivered as intended (fidelity))	7, 12-13
	24b	Concomitant care received during the trial for each group	N/A
Baseline data	25	A table showing baseline demographic and clinical characteristics for each group	Table 1
Numbers analysed, outcomes and estimation	26	For each primary and secondary outcome, by group:<br>● the number of participants included in the analysis<br>● the number of participants with available data at the outcome time point<br>● result for each group, and the estimated effect size and its precision (such as 95% confidence interval)<br>● for binary outcomes, presentation of both absolute and relative effect size	7-13
Harms	27	All harms or unintended events in each group	12-13, Sup Table 2
Ancillary analyses	28	Any other analyses performed, including subgroup and sensitivity analyses, distinguishing pre-specified from post hoc	7-13
**Discussion**			
Interpretation	29	Interpretation consistent with results, balancing benefits and harms, and considering other relevant evidence	14-17
Limitations	30	Trial limitations, addressing sources of potential bias, imprecision, generalisability, and, if relevant, multiplicity of analyses	16

Citation: Hopewell S, Chan AW, Collins GS, Hróbjartsson A, Moher D, Schulz KF, et al. CONSORT 2025 Statement: updated guideline for reporting randomised trials. BMJ. 2025; 388:e081123. https://dx.doi.org/10.1136/bmj-2024-081123
© 2025 Hopewell et al. This is an Open Access article distributed under the terms of the Creative Commons Attribution License (https://creativecommons.org/licenses/by/4.0/), which permits unrestricted use, distribution, and reproduction in any medium, provided the original work is properly cited

PREDIX Luminal B - Neoadjuvant response-guided treatment of estrogen receptor positive tumors with high proliferation or slow proliferation with metastatic nodes

Part of a platform of translational phase II trials based on molecular subtypes

LumB

Protocol version 7.0   21 July 2025

EudraCT number: 2014-000810-72

Sponsor: Thomas Hatschek
Dept. of Oncology
Karolinska University Hospital,
Stockholm, Sweden

Co-ordinating investigator:	Thomas Hatschek
Study director:	Jonas Bergh
	Karolinska University Hospital and Karolinska Institutet

____________________ ______________________________________

Date signed Co-ordinating investigator

____________________ ______________________________________

Date signed Principal investigator

SYNOPSIS

Title: PREDIX Luminal B - Neoadjuvant response-guided treatment of estrogen receptor positive tumors with high proliferation or slow proliferation with metastatic nodes– part of a platform of translational randomized phase II trials based on molecular subtypes.

Design: Phase II randomized trial evaluating response-guided treatment in luminal B breast cancer without lymph node metastases or luminal A breast cancer with lymph node metastases and/or in women 35 years of age or younger.

Rationale: Adjuvant polychemotherapy has improved disease-free and overall survival after breast cancer surgery (EBCTCG, 2012). Randomized trials have shown that preoperative chemotherapy increased the proportion of cases available for breast-conserving surgery, but studies comparing postoperative and preoperative chemotherapy have so far not demonstrated any significant difference in disease-free and overall survival between these two options. However, in the subgroup of patients with tumors responding with a pathologic complete response (pCR) to preoperative treatment, survival was prolonged compared with women with less responsive tumors. Based on this knowledge, a second generation of trials investigated the efficacy of different drug combinations or treatment strategies with the intention to improve tumor response and outcome. An unplanned subgroup analysis on one of these trials, GeparTrio, revealed that neoadjuvant therapy is a useful tool to decide upon change of therapy due to lack of response, or extended treatment in case of response. It was also found that the impact of pathologic response for the outcome differs between the subgroups defined by genomic patterns (von Minckwitz et al. 2013). While pCR appeared to be a surrogate predictor of favorable outcome in luminal B, HER2 hormone receptor negative and triple-negative tumors, switch of therapy in cases of no response after two cycles of chemotherapy seemed to be the best concept for luminal A and HER2/luminal B tumors.

Similar to adjuvant chemotherapy, hormonal treatment with tamoxifen or an aromatase inhibitor has, in adjuvant trials including thousands of patients, shown a significant reduction of disease-free and overall survival among patients with hormone receptor positive breast cancer, approximately 70-80 % of all patients (EBCTCG 2011).

In contrast to adjuvant therapy, preoperative treatment allows for individualized *in vivo* assessment of sensitivity to treatment, information which is unavailable in the postoperative setting. The basis of the PREDIX platform is to offer patients with primary breast cancer medical treatment in relation to the molecular subtypes

1. Luminal A;
2. Luminal $A_{Npalp/\leq 40\ years}$/Luminal B;
3. HER2 amplified;
4. Triple-negative.

The present protocol covers a more aggressive group of hormone receptor-positive tumors, where the majority is classified as luminal B, characterized by high ER and low PR expression. Slowly proliferating hormone receptor positive tumors have different biological properties compared with those with high mitotic count. Data

from clinical trials addressing preoperative endocrine treatment of these tumors is limited (Eiermann et al. 2001; Smith et al. 2005), possibly due to the low frequency of objective response reported from these trials and chemotherapy is at present the treatment of choice, even in slowly proliferating tumors.

Palbociclib is a selective inhibitor of the cyclin-dependent kinases CDK4 and 6. CDK4 activity is restricted to the G1-S phase and controlled by Cyclin D1, CDK6 has impact on the G1 phase progression and G1/S transition, controlled by Cyclin D2 (Rocca et al 2014). Palbociclib inhibits the CDK-regulated Rb phosphorylation at Ser^{780}, thereby preventing thymidine incorporation into the DNA. The inhibition has been reported as restricted to Rb positive cells (Fry et al 2004). In tamoxifen-resistant MCF7 cell lines, palbociclib was found to enhance sensitivity for tamoxifen (Finn et al 2009).

Clinical results for palbociclib as neoadjuvant treatment are not yet published. The design of a recently launched randomized phase II trial, NEOPAL, comparing neoadjuvant chemotherapy with letrozole + palbociclib has been presented (Cottu PH et al, SABCS 2014 Abstract OT3-2-06). However, data from an early neoadjuvant trial investigating the potential of palbociclib added to an aromatase inhibitor to achieve cell cycle arrest (Complete Cell Cycle Arrest, CCCA, as primary endpoint) succeded in demonstrating cell cycle control in the majority of patients, although reversible within few weeks after termination of the treatment (Ma C et al 2017).

In a phase II randomized trial including postmenopausal patients with advanced HER2 negative hormone receptor positive breast cancer, letrozole in combination with 125 mg palbociclib was compared with letrozole and placebo. A final update (PALOMA-1) has been published recently (Finn RS et al 2015). The trial revealed a highly significant prolongation of the median PFS from 10.2 to 20.2 months in favor of the combined treatment (HR 0.49, 95 % CI 0.32-0.75, p=0.0004). Median overall survival (OS) was prolonged from 33.3 to 37.5 months in the combination arm, but the study was not powered to show a significant difference (HR 0.81, p=0.21).

A phase III comparison of palbociclib + fulvestrant with fulvestrant + placebo (PALOMA-3) in patients who had progressive disease on previous endocrine treatment showed a median progression-free survival of 9.5 months in the palbociclib/fulvestrant arm, compared with 4.6 months in the fulvestrant/placebo arm (Cristofanilli M et al 2016).

Reported side effects due to palbociclib have been moderate in the PALOMA-1 trial: grade 3/4 neutropenia occurred in 48/6 %, leukopenia in 19/0 % of patients, anemia in 5/1 %. Non-hematological toxicity was rare, with diarrhea as the most commonly reported grade 3 adverse event, 4 %. Similar observations had been previously observed in a phase I trial (Flaherty et al 2012).

Management procedures during the neoadjuvant treatment period:

Before randomization, assignment for the PREDIX LumB trial must be confirmed by immunohistochemistry including estimation of ER, PR and standardized proliferation marker.

In patients without palpable axillary lymph node metastases, sentinel node biopsy (SNB) is recommended standard procedure, but must not be performed closer than 7 days prior to start of therapy.

Patients are randomized to *either* weekly treatment with paclitaxel (arm A) *or* endocrine treatment in combination with palbociclib (arm B) for 12 weeks. Choice of endocrine treatment is for pre- and perimenopausal women and all men tamoxifen 20 mg daily, alternatively for women in this age cohort, a LHRH analogue in combination with an aromatase inhibitor, for all postmenopausal women treatment with an aromatase inhibitor. The aromatase inhibitors to be used according to local practice are anastrozole 1 mg daily, exemestane 25 mg daily, or letrozole 2.5 mg daily. Pre- or perimenopausal women and all men are treated with tamoxifen , alternatively with an LHRH analogue in combination with an aromatase inhibitor (only women). Postmenopausal women receive an aromatase inhibitor.

After 12 weeks, patients without signs of disease progression (PD) are switched to *either* endocrine treatment in combination with palbociclib (arm A) *or* weekly treatment with paclitaxel (arm B) for 12 weeks.

Before start, after 6, 12, 18 and 24 weeks of treatment, radiological assessments of tumor size are performed using mammography and ultrasound alt. MRI breast; PET-CT, confined to the breast and regional lymph nodes, before start, after 12 and 24 weeks, blood tests before start, after 12, 18 and 24 weeks. Physical examinations are performed before start and then four-weekly after 4, 8, 12, 16, 20 and 24 weeks of treatment.

In case of disease progression during ongoing study treatment, individualized management in the patient's best interest must be considered, in which case surgery is the primary option.

General aims of the study:

1. Evaluate the impact of the allocated treatment on objective response and long-term outcome.
2. Identify tumor characteristics and treatment-related changes of tumor biology predictive of *long-term prognosis.*

Objectives: ***Primary endpoint:***

Absolute difference in clinical and radiological objective response after completion of the first 12-week period of primary medical treatment.

Secondary endpoints:

Pathological objective response to primary medical treatment

Sequencing of chemotherapy *versus* endocrine treatment plus palbociclib

Morphological, functional and biological characteristics of tumors exposed to cytotoxic and targeted treatment of early breast cancer

Disease-free, breast cancer-specific and overall survival

Event-free survival (EFS), invasive disease-free survival (IDFS), distant disease-free survival (DDFS), breast cancer-specific survival (BCSS) and overall survival (OS)

Safety

Quality of life

Frequency of breast-conserving surgery.

Subjects:

1. Written informed consent
2. Patients with breast cancer confirmed by histology
3. Tumor and blood samples available. *Luminal type* confirmed by immunohistochemistry with ER ≥10 %, *not* HER2 3+/amplified
4. Age 35 years or older. Elderly patients in condition adequate for planned therapy
5. Primary breast cancer >20mm in diameter *and/or* verified regional lymph node metastases
6. Adequate bone marrow, renal, hepatic and cardiac functions and absence of other uncontrolled medical or psychiatric disorders
7. ECOG performance status 0-1
8. Presence of at most 2 morphologically characterized well-defined distant metastases accessible for stereotactic radiotherapy, provided that this treatment is available.

Treatment of hormone receptor positive breast cancer:

Patients are randomized to *either* weekly treatment with paclitaxel *or* endocrine therapy in combination with the cdk 4/6 inhibitor palbociclib:

Dose regimen weeks 1 to 12 of treatment:

Arm A:

Weekly paclitaxel on days 1, 8, 15, 22 etc., starting dose 80 mg/m^2. Doses can thereafter be individually adjusted in the range between 70 and 90 mg/m^2 in relation to side effects. In case of no change or decrease of the tumor after six weeks, this treatment is continued for further six weeks. Upon progression (PD), treatment is prematurely switched to the 2nd sequence.

In cases with repeated severe infusion-related reactions motivating discontinuation, treatment with paclitaxel can be replaced by nab-paclitaxel (Abraxane®), 125 mg/m^2 for the remaining treatment period, see **4.3 g**.

Arm B:

Patients are treated with tamoxifen (pre- or perimenopausal women, all men), alternatively for women in this age cohort, a LHRH analogue in combination with an aromatase inhibitor, or an aromatase inhibitor (postmenopausal patients) in combination with the cdk 4/6 inhibitor palbociclib 125 mg orally days 1-21, followed by a 7-days rest period. In cases with uncertain menopausal status (previous hysterectomy and equivocal gonadotropins), postmenopause age limit is defined as 55 years or older. Dose reduction of palbociclib to 100 mg (step -1) or 75 mg (step -2) in relation to side effects. Upon progression (PD), treatment is prematurely switched to the 2nd sequence.

After the initial 12-week period, treatment is switched to the opposite regimen.

Dose regimen weeks 13 to 24 of treatment:

Arm A:

Treatment is switched to tamoxifen 20 mg daily (pre- or perimenopausal patients, men) or an aromatase inhibitor, either anastrozole 1 mg daily, exemestane 25 mg daily, or letrozole 2.5 mg daily (postmenopausal patients) in combination with the cdk 4/6 inhibitor palbociclib 125 mg orally days 1-21, followed by a 7-days rest period. In cases with uncertain menopausal status (previous hysterectomy and equivocal gonadotropins), postmenopause age limit is defined as 55 years or older. Dose reduction of palbociclib to 100 mg (step -1) or 75 mg (step -2) in relation to side effects. The treatment is continued for totally 12 weeks, provided that re-evaluation during treatment does not indicate progression. Upon progression (PD), individualized management, preferentially surgery, is the primary option.

In cases with repeated severe infusion-related reactions motivating discontinuation, treatment with paclitaxel can be replaced by nab-paclitaxel (Abraxane®), 125 mg/m^2 for the remaining treatment period, see **4.3 g**.

Arm B:

Treatment is switched to weekly paclitaxel on days 1, 8, 15, 22 etc., starting dose 80 mg/m^2. Doses can thereafter be individually adjusted in the range between 70 and 90 mg/m^2 in relation to side effects. The treatment is continued for totally 12 weeks, provided that re-evaluation after six weeks (18 weeks after start) does not indicate progression. Upon progression (PD), individualized management, preferentially surgery, is the primary option.

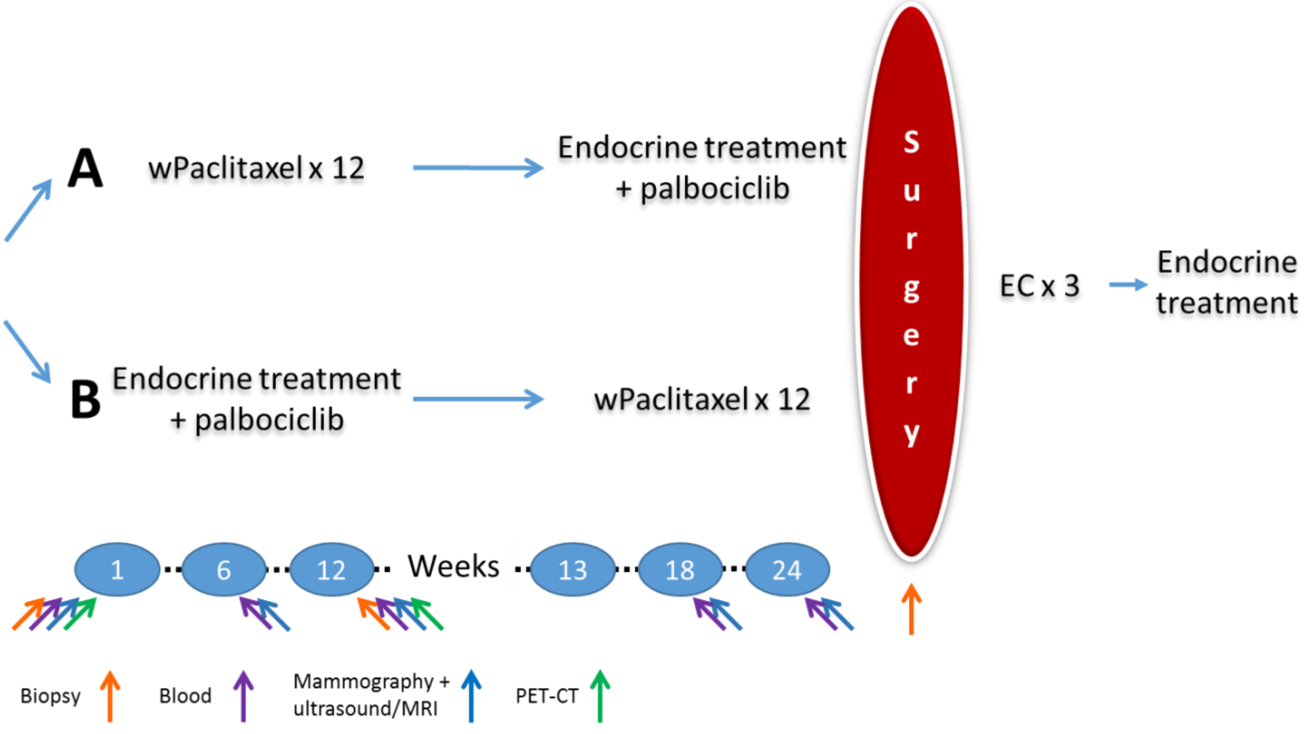

Postoperatively, all patients are offered chemotherapy with a combination containing anthracycline, $E_{75\text{-}100}C_{\geq 500}$ x 3 every three weeks. All patients are recommended continuous adjuvant endocrine treatment for at least five years.

Schedule of treatment evaluations:

Patients will undergo the following examinations before, during and after treatment:

Within 6 weeks before start:
Bilateral mammography, ultrasound or MRI breast

Diagnostic biopsies
SNB at latest 1 week before start

Within 2 weeks before start:
Physical examination
PET-CT (if available)
Core biopsies
Blood samples/tests (study and see ch. 4.6)
HRQoL

Randomisation

Weeks 1, 2, 3:
Blood tests WBC, ANC

Week 4:
Physical examination
Blood tests (WBC, ANC, see 4.7)

Week 6:
Bilateral mammography, ultrasound or MRI breast
Blood samples (study)
HRQoL

Week 8:
Physical examination
Blood tests (WBC, ANC, see 4.7)

Week 12:
Physical examination
Bilateral mammography, ultrasound or MRI breast
PET-CT (if available)
Core biopsies
Blood samples/tests (study and see ch. 4.7)
HRQoL

Switch of treatment

Weeks 13, 14, 15:
Blood tests WBC, ANC

Week 16:
Physical examination
Blood tests (WBC, ANC, see 4.7)

Week 18:
Bilateral mammography, ultrasound or MRI breast
Blood samples
HRQoL

Week 20:
Physical examination
Blood tests (WBC, ANC, see 4.7)

Week 24:
Physical examination
Bilateral mammography, ultrasound or MRI breast
Blood samples/tests (study and see ch. 4.7)
HRQoL

Treatment termination

3-4 weeks after termination:
Breast/axillary surgery
Biopsies from the surgical specimen

End-of Treatment visit 3 months after surgery:
Physical examination
HRQoL

Annual follow-up visits:
Physical examination
Bilateral mammography, ultrasound or MRI breast
Blood samples (study)
HRQoL

At recurrence:
Radiological staging
FNAs from recurrent sites
Blood samples (study)

Evaluation tools:

Mammography, ultrasound alt. MRI breast, PET-CT, confined to the breast and regional lymph nodes. Annual mammograms in connection with follow-up visits.

Ultrasound-guided tumor biopsies.

Blood samples (plasma and serum) during treatment and in connection with annual follow-up visits.

At recurrence:

Aspirates from sites of local recurrence or distant metastases or tumor in the contralateral breast if accessible for fine-needle aspiration (FNA) and preserved in RNA*later*. Blood samples (plasma and serum).

Safety evaluation:

Safety will be assessed by use of clinical Adverse Events and Common Toxicity Criteria (CTC) laboratory and non-laboratory toxicities. NCI Common Terminology Criteria for Adverse Events v4.0 (CTCAE) will be used.

At the time of enrolment, a baseline status is documented to note the occurrence and nature of each patient's medical conditions.

During treatment, CTC toxicity rating will be performed after each cycle for any adverse event that has been experienced during the previous cycle. Adverse event reporting is documented in the clinical Case Report Form (CRF). AE/SAE reporting covers the period from 1st dose of palbociclib until 28 calender days after last administration until resolution.

Special attention is given to the following adverse reactions:

1. Elevation of liver transaminases
2. Infusion-related and hypersensitivity reactions
3. Hematologic reactions.

Serious Adverse Event (SAE):

1. Death
2. Hospitalization initiated or prolonged due to an adverse event, except for prophylactic hospitalization to prevent repetition of a previous toxic reaction
3. A life threatening experience
4. Severe or permanent disability
5. Congenital anomaly, birth defect, neonatal death
6. Any other event considered to be an important medical event.

An SAE must be reported by the study personnel immediately upon awareness if the SAE is fatal or life threatening regardless of the extent of available information, within 24 h of first awareness if the SAE is not fatal or life threatening. The report is sent to the CTO, KPE, Radiumhemmet, fax +46 (0)8-306989. Preplanned hospitalizations are not regarded as an SAE.

Safety tools: Physical examination

Performance status

Blood counts, biochemistry

Reporting side effects after every treatment cycle and during follow-up annually for five years.

Surgery, postoperative treatment, follow-up:

Surgery, breast-conserving or mastectomy w/o reconstruction is performed approximately 2 weeks after termination of the medical treatment.

In case of absence of lymph node metastases confirmed through SNB before start of treatment, further axillary surgery in connection with breast surgery can be omitted. Micro metastases should be followed by repeated SNB, macro metastases by axillary clearance.

Radiotherapy of the breast after partial mastectomy, of the thoracic wall after mastectomy in case of large or multifocal tumors or due to narrow margins to underlying tissue. Regional radiotherapy is recommended in all cases with metastatic nodes found after pretreatment SNB or axillary node dissection. Follow-up reported yearly for ten years.

Adjuvant anthracycline-based chemotherapy after surgery and before radiotherapy for all patients.

Adjuvant endocrine treatment for at least five years is recommended for all patients with hormone receptor positive tumors.

Sample size, statistics:

The PREDIX LumB trial is based on current knowledge of molecular characteristics of breast cancer and designed primarily to investigate if response to endocrine treatment can be improved by inhibition of cdk 4 and 6 in comparison with standard chemotherapy, and, secondary, evaluate the impact of sequencing of the treatment options, find predictors of event-free, disease-free and overall survival, and observe toxicity, quality of life and changes in the surgical management in the two treatment groups (secondary endpoints).

The sample size is based on an explorative design: the primary objective is to compare clinical/radiological objective response *after completion of the first 12-week period* of neoadjuvant treatment.

Assuming an objective (partial or complete) response after 12 courses of weekly paclitaxel (standard treatment arm A) in approximately 30 % of cases, an absolute difference in response by 20 % between treatment alternatives A and B is considered clinically relevant. With alpha 0.10 (two-sided test) and power 0.80, 166 patients, 83 per treatment arm, are required. Differences in response rate will be tested using Fisher's exact test. The goal is to randomize 180 patients in the trial.

For patients with progressive disease (increase of tumor size by more than 20 %) at any time, individualized management, preferentially surgery, is the primary option.

Due to the explorative design, results from this trial need to be confirmed by phase III trials with more stringent statistical requirements.

Timelines: Sign-off date of the study protocol: November 2014

First patient in: February 2016

Last patient in: December 2021

Final clinical study report: December 2026

References:

Early Breast Cancer Trialists' Collaborative Group (EBCTCG). Relevance of breast cancer hormone receptors and other factors to the efficacy of adjuvant tamoxifen: patient-level meta-analysis of randomised trials. Lancet 2011;378:771–84

Early Breast Cancer Trialists' Collaborative Group (EBCTCG). Comparisons between different polychemotherapy regimens for early breast cancer: meta-analyses of long-term outcome among 100 000 women in 123 randomised trials. Lancet 2012;379(9814):432-44

Eiermann W, Paepke S, Appfelstaedt J, et al. Preoperative treatment of postmenopausal breast cancer patients with letrozole: A randomized double-blind multicenter study. Ann Oncol 2001;12(11):1527-32

Finn RS, Crown JP, Boer K, et al. Results of a randomized phase 2 study of PD 0332991, a cyclin-dependent kinase (CDK) 4/6 inhibitor, in combination with letrozole vs letrozole alone for first-line treatment of ER+/HER2-advanced breast cancer (BC). Ann Oncol 2012;23, Suppl. 2

Finn RS, Crown JP, Lang I, et al. The cyclin-dependent kinase 4/6 inhibitor palbociclib in combination with letrozole versus letrozole alone as fi rst-line treatment of oestrogen receptor-positive, HER2-negative, advanced breast cancer (PALOMA-1/TRIO-18): a randomised phase 2 study. Lancet Oncol 2015; 16: 25–35

Ma CX, Gao F, Luo J, et al. NeoPalAna: Neoadjuvant palbociclib, a cyclin-dependent kinase 4/6 inhibitor, and anastrozole for clinical stage 2 or 3 estrogen receptor positive breast cancer. Clin Cancer Res. 2017 Mar 7. doi: 10.1158/1078-0432.CCR-16-3206. [Epub ahead of print]

Cristofanilli M, Turner NC, Bondarenko I, et al. Fulvestrant plus palbociclib versus fulvestrant plus placebo for treatment of hormone-receptor-positive, HER2-negative metastatic breast cancer that progressed on previous endocrine therapy (PALOMA-3): final analysis of the multicentre, double-blind, phase 3 randomised controlled trial. Lancet Oncol. 2016 Apr;17(4):425-39

Flaherty KT, Lorusso PM, Demichele A, et al. Phase I, dose-escalation trial of the oral cyclin-dependent kinase 4/6 inhibitor PD 0332991, administered using a 21-day schedule in patients with advanced cancer. Clin Cancer Res 2012;18(2):568-76

Fry DW, Harvey PJ, Keller PR, et al. Specific inhibition of cyclin-dependent kinase 4/6 by PD 0332991 and associated antitumor activity in human tumor xenografts. Mol Cancer Ther, 2004; 3(11), 1427-1438

Rocca A, Farolfi A, Bravaccini S, et al. Palbociclib (PD 0332991): targeting the cell cycle machinery in breast cancer. Expert Opin Pharmacother 2014;15(3):407-420

Smith IE, Dowsett M, Ebbs SR, et al. Neoadjuvant treatment of postmenopausal breast cancer with anastrozole, tamoxifen, or both in combination: the Immediate Preoperative Anastrozole, Tamoxifen, or Combined with Tamoxifen (IMPACT) multicenter double-blind randomized trial. J Clin Oncol. 2005;23(22):5108-16

von Minckwitz G, Blohmer JU, Costa SD. Response-guided neoadjuvant chemotherapy for breast cancer. J Clin Oncol 2013;31(29):3623-30.

	Before treatment		Neoadjuvant therapy										Surgery	Adjuvant CT	Follow-up			
			Weeks 1-3	Week 4	Week 6	Week 8	Week 12	Weeks 13-15	Week 16	Week 18	Week 20	Week 24						
	<6 wks	<2 wks	Hema-tology	Evalu-ation	Evalu-ation	Evalu-ation	Evalu-ation	Hematology	Evalu-ation	Evalu-ation	Evalu-ation	Evalu-ation			EoT[2]	Years 1-5	Years 6-10	Recur-rence
Informed Consent	●																	
Physical exam		●		●		●	●		●		●	●			●	●		●
Mammography + u-sound/MRI	●				●		●			●		●				●		
Diagnostic pathology	●												●					
Core biopsies (5, 14G needle)		●					●						●					
Fine-needle aspirate (FNA)																		●
Blood tests (see 4.6 and 4.7)*		●	●[1]	●		●	●	●[1]	●		●	●			●			
Blood samples (study)		●			●		●			●		●			●	●		●
PET-CT breast		●					●											
HRQoL		●			●		●			●		●			●	●		
SNB/axillary exploration	●												●					
Reporting recurrence/death																●	●	●
Treatment			A: Weekly paclitaxel vs. B: Endocrine treatment + palbociclib					A: Endocrine treatment + palbociclib vs. B: Weekly paclitaxel						EC	Adjuvant therapy			

[1]: weekly blood tests (WBC, ANC) during the first four weeks with palbociclib, then 4-weekly; [2]: EoT End of Treatment

TABLE OF CONTENTS

SYNOPSIS 3
1 INTRODUCTION 18
1.1 General 18
1.2 Hormone receptor positive breast cancer 22
2 MANAGEMENT, AIMS AND OBJECTIVES 24
2.1 Management procedures 24
2.2 Aims 24
2.3 Objectives 24
2.3.1 Primary endpoint 24
2.3.2 Secondary endpoints 24
3 PATIENT SELECTION CRITERIA 25
3.1 Inclusion criteria 25
3.2 Exclusion criteria 25
4 STUDY TREATMENT 25
4.1 Investigational drug 25
4.2 Comparative drugs 26
4.2.1 Paclitaxel 26
4.3 Treatment schedule and doses 26
4.4 Premedication 28
4.5 Precautions 28
4.6 Laboratory values before start of treatment 29
4.7 Laboratory tests during treatment 29
4.8 Toxicity of palbociclib 29
4.9 Drug interactions of palbociclib 30
4.10 Dose adjustments of palbociclib 30
5 TREATMENT EVALUATION SCHEDULES 32
5.1 Response evaluation 32
5.1.1 Before start of treatment 32
5.1.2 During treatment 32
5.1.3 After treatment and during follow-up 33
5.2 Safety evaluation 33
5.2.1 Adverse event reporting 33

5.3 Treatment after completion of preoperative therapy 35
5.4 End-of-treatment/Follow-up 35
6 RESPONSE MEASURES 36
6.1 Measurable disease by radiology 36
6.2 Clinical examination and criteria for measurement of clinical response 36
6.3 Pathologic evaluation of tumor response 37
6.4 References 38
7 RADIOLOGICAL EVALUATION 39
7.1 Mammography and conventional ultrasound 39
7.1.1 Background 39
7.1.2 Methods 39
7.2 Positron Emission Tomography (PET-CT) 40
7.2.1 Background 40
7.2.2 Methods 41
7.3 References 41
8 MAMMOGRAPHIC DENSITY 42
8.1 Background 42
8.2 Methods 42
8.3 References 43
9 QUALITY OF LIFE ASSESSMENT 43
9.1 Background 43
9.2 Methods 44
´9.3 References 45
10 NEXT-GENERATION SEQUENCING 46
10.1 Background 46
10.2 Methods 47
10.3 References 47
11 PHARMACOGENETICS 48
11.1 Background 48
11.2 Methods 49
11.3 References 49
12 INTRA-TUMOR HETEROGENEITY BY SINGLE-CELL SEQUENCING 50
12.1 Background 50
12.2 Methods 50
12.3 References 51

13 SMALL RNAs IN TUMOR AND PLASMA/SERUM 52
13.1 Background 52
13.2 Methods..52
13.3 References 53
14 TUMOR AND PLASMA PROTEOMICS 53
14.1 Background 53
14.2 Methods..54
14.3 References 54
15 STEM CELL ANALYSES 55
15.1 Background 55
15.2 Methods..55
15.3 References 55
16 CHARACTERIZATION OF TUMOR STROMA 55
16.1 Background 55
16.2 Methods..56
16.3 References 56
17 AMOTL2 EXPRESSION IN EARLY BREAST CANCER 57
17.1 Background 57
17.2 Methods..57
17.3 References 58
18 FNA-based profiling of mRNA and proteins..58
18.1 Background 58
18.2 Methods..58
18.3 References 59
19 IMMUNOLOGICAL PROFILING 59
19.1 Background 59
19.2 Methods..60
19.3 References 60
20 PROFILING OF RECURRENCE/NEW PRIMARY DURING FOLLOW-UP ...61
20.1 Background 61
20.2 Methods..61
20.3 References 62
21 STUDY ADMINISTRATION..63
21.1 General rules 63
21.2 Screening and enrolment..63

21.3 Patient information and Informed Consent 63
21.4 Registration 63
21.5 Data management and study database 63
21.6 Source data 65
21.7 Quality control and assurance 65
21.8 Biobanking 65
21.9 Saving of medical records 65
22 ETHICS 66
23 STATISTICS 66
24 PUBLICATION POLICY 67
25 HANDLING OF TISSUE AND BLOOD SAMPLES 69
25.1 Tissue samples 69
25.2 Blood samples 70
25.3 Schedule for biological samples 72
26 SUMMARY OF TRANSLATIONAL STUDIES 73
27 Appendix: Study specific SAE form 74

1 INTRODUCTION

1.1 General

1.1.1 Breast carcinoma

Breast cancer is the most common tumor in women, below one per cent in males. In the Western world, one of ten women will contract the disease. In Sweden, approximately 8400 women are diagnosed with breast cancer every year with an increase by 1-2 %.

Surgery has for a long period of time been the primary treatment with the intention to cure. Postoperative radiotherapy reduces the risk of local recurrence, but has also a significantly favorable impact on survival.

The impact of adjuvant systemic treatment on prognosis has been investigated by numerous trials and follow-up within the frame of quinquennial meta-analyses performed by the EBCTCG have proved consistent gains in recurrence-free and overall survival up to 20 years after treatment (Early Breast Cancer Trialists' Collaborative Group). These results are the basis for worldwide used standard-of-therapy guidelines.

The present classification of breast cancer is based on descriptive morphology with ductal cancer being the most frequent, 70-80 %, followed by lobular cancer in 15-20 %. Other subtypes are diagnosed in less than 5 % of cases.

1.1.2 Neoadjuvant treatment of breast cancer – clinical data

Neoadjuvant treatment is in the majority of cases delivered as cytotoxic therapy, in HER2 positive tumors together with trastuzumab alone, or in combination with pertuzumab (Neosphere) or lapatinib (NeoALLTO). Endocrine treatment is rather an exception, used in elderly patients with locally advanced breast tumors. The most common indication for primary medical treatment outside of clinical trials is reduction of tumor size to improve the chance of radical and, if possible, breast-conserving surgery. A current trend is to use the response of therapy on the primary tumor and regional nodes as a surrogate measure for efficacy also on micrometastases in the general host environment. The use of systemic treatment is motivated since the diagnosis of breast cancer involves a potential risk of systemic disease and should be treated like that. Metastatic disease is the most unfavorable factor in terms of survival.

Several large randomized trials have evaluated the effect of preoperative cytotoxic treatment in comparison to the same treatment given adjuvant after surgery. None of these trials has yet shown an improvement in outcome with respect to survival. However, there is evidence that pathologic complete response (pCR) is a significant indicator for prolonged survival.

In operable breast cancer, randomized studies have shown that neoadjuvant chemotherapy enhances the breast conservation rate (Fisher et al 1998, Mauriac et al 1991, Powles et al 1995, Scholl et al 1994) without compromising survival (Fisher et al 1998, Mauriac et al 1991, Mauriac et al 1999, Scholl et al 1994, Semiglazov et al 1994). The addition of preoperative docetaxel following treatment with AC x 4 increased the clinical response rate from 86 to 91 percent and the pCR rate from 14 to 26 percent compared with AC alone (Bear et al 2006).

A Cochrane Review compiled results from a total of 14 randomized trials including 5500 women (Mieog JSD et al 2007). No differences in disease-free or overall survival were found in a comparison of pre- *versus* postoperative chemotherapy. The chance to perform breast conserving surgery was significantly improved (HR 0.82, 95% CI 0.76-0.89), but there was also a non-

significant increase of loco-regional recurrence (HR 1.12, 95 % CI 0.92-1.37) for patients treated with preoperative chemotherapy, which was not found as long as surgery was part of the primary treatment also in patients with complete tumor response. In the subgroup of patients who experienced pathologic complete response (pCR), survival was significantly improved compared to patients with residual disease (HR 0.48; 95 % CI 0.33-.069).

The most powerful cytotoxic drugs evaluated in primary systemic treatment of early breast cancer are the anthracyclines doxorubicin and epirubicin and the taxanes docetaxel and paclitaxel. Experiences are good from adjuvant therapy, but limited from preoperative treatment with drugs. In the NSABP B-27 trial, a sequential design was applied, where anthracycline, in combination with cyclophosphamide, was followed by docetaxel (Bear et al 2006). Although there was no significant difference in terms of DFS and OS, the frequency of pCR was almost doubled to 19 percent by preoperative addition of docetaxel. In the GeparTrio trial (von Minckwitz et al 2013), the combination TAC was evaluated after two cycles. Responders were randomized to either further four *or* six cycles with the same regimen, non-responders to either four cycles of TAC *or* switch to four cycles of capecitabine and vinorelbin. Response, estimated by ultrasound after the first two cycles, was found in 69.1 % *versus* 30.9 % lack of response. Extended treatment (responders) or switch of therapy (non-responders), defined as *response-guided treatment* led to a significant improvement of disease-free survival (HR 0.71, 95 % CI 0.60-0.85, $p<0.001$) and overall survival (HR 0.79, 95 % CI, 0.63-0.99, $p=0.048$). Subgroup analyses revealed that triple negative (ER-, PR-, HER2-negative) and grade III tumors gained most from the treatment.

Similar to adjuvant chemotherapy, hormonal treatment with tamoxifen or an aromatase inhibitor has, in adjuvant trials including thousands of patients, shown a significant reduction of disease-free and overall survival among patients with hormone receptor positive breast cancer, approximately 70-80 % of all patients (EBCTCG 2011). Five years of treatment are standard, during recent years published data advocate prolonged treatment (Higgins MJ et al. 2013). However, half of the patient population receiving five years of endocrine treatment will experience relapse during or after the adjuvant treatment. Who of these patients were non-responsive to the five years or more hormonal treatment, and who benefitted from this treatment is yet unknown. The response to this therapy is slow which requires prolonged treatment, and complete response is rare. Therefore, primary endocrine treatment is rarely used, mostly in elderly patients, or those with poor general condition. The present protocol covers hormone receptor positive tumors. Slowly proliferating hormone receptor positive tumors have different biological properties compared with those with high mitotic count. Data from clinical trials addressing preoperative endocrine treatment of these tumors is limited (Eiermann et al. 2001; Smith et al. 2005), possibly due to the low frequency of objective response reported from these trials. There is urgent need to increase knowledge about endocrine responsiveness in early endocrine treatment of breast cancer.

Palbociclib is a selective inhibitor of the cyclin-dependent kinases CDK4 and 6. CDK4 activity is restricted to the G1-S phase and controlled by Cyclin D1, CDK6 has impact on the G1 phase progression and G1/S transition, controlled by Cyclin D2. Palbociclib inhibits the CDK-regulated Rb phosphorylation at Ser^{780}, thereby preventing thymidine incorporation into the DNA. The inhibition has been reported as restricted to Rb positive cells (Fry et al 2004). In tamoxifen-resistant MCF7 cell lines, palbociclib was found to enhance sensitivity for tamoxifen (Finn et al 2009).

Clinical results for palbociclib as neoadjuvant treatment are not yet published. The design of a recently launched randomized phase II trial, NEOPAL, comparing neoadjuvant chemotherapy with letrozole + palbociclib has been presented (Cottu PH et al, SABCS 2014 Abstract OT3-2-06).). However, data from an early neoadjuvant trial investigating the potential of palbociclib added to an aromatase inhibitor to achieve cell cycle arrest (Complete Cell Cycle Arrest, CCCA, as primary

endpoint) succeded in demonstrating cell cycle control in the majority of patients, although reversible within few weeks after termination of the treatment (Ma C et al 2017).

Lessons learned from neoadjuvant trials:

The sequence of medical treatment and surgery does not affect the outcome, if the medical treatment is given according to a fixed schedule, even though the subgroup of cases with complete pathologic tumor response have a more favorable outcome.

The outcome of response-guided alternatives is superior to standard therapy both in terms of disease-free and overall survival.

Subgroup analyses using gene expression pattern-based subtypes luminal A, luminal B, HER2+/luminal B, HER2+/hormone receptor negative and triple negative breast cancer (TNBC) showed that pCR was a highly significant predictor of outcome in HER2+/hormone receptor negative and TNBC, more limited in tumors classified as luminal B with or without HER2+, and of no use in luminal A tumors.

1.1.3 Risk assessment – from staging to molecular "intrinsic" subgroups

The decision on *if* patients should be recommended adjuvant chemotherapy is mainly based on current prognostic factors, predominantly the stage of the disease at diagnosis, supported by the grade of malignancy and markers of proliferation determined by immunohistochemistry (IHC) on tumor tissue.

The decision on *what type* of adjuvant treatment is most appropriate is based on IHC-based predictive factors including estrogen (ER) and progesterone receptor (PR), markers of proliferation and HER2 by IHC or *in situ* hybridisation (ISH). While IHC leaves the assessment to the observers' experience, ISH has been shown to be a more reliable method in terms of reproducibility. Results from the Swedish Quality Assurance (SweQA) group have shown that interobserver reproducibility was high for HER2 ISH, well acceptable for HER2 IHC (Rydén et al. 2009), ER and PR (Cohen et al. 2012). Most divergent results have been obtained for proliferation markers, mainly Ki67 (Vörös et al. 2012).

As a consequence of the development of gene-expression assays, alternative risk assessments, Oncotype DX™ and Mammaprint™, have been developed and are in clinical use or are prospectively tested as prognostic factors (Dobbe et al. 2008). These assays are expected to support clinical decision-making to select patients who should or should not be offered adjuvant therapy.

In contrast to the established histological classification of tumors (ductal, lobular etc.), the development of gene expression techniques enables a new classification based on gene patterns with the ability to characterize tumors due to function rather than anatomy (Parker et al. 2008; Prat et al. 2012). This classification splits breast cancer into different subgroups based on the known predictive factors ER, PR and proliferation (Luminal A and B), HER2 status and a group of yet heterogeneous triple-negative breast cancer, TNBC with unknown predictive factors, non-ER non-PR non-HER2.

1.1.4 Adjuvant *versus* primary systemic treatment (PST)

Despite convincing results from all categories of adjuvant treatment the fact remains that several thousands of randomized patients are necessary to prove minor differences in outcome, and it remains even after completion and 5-10 years follow-up unknown, *who* of the treated patients benefitted from the treatment. Adjuvant therapy is "blinded" and makes eventual benefits or failure of a treatment unpredictable.

In contrast, neoadjuvant, or primary systemic therapy (PST), allows for an assessment of tumor response *in vivo*. This strategy is useful for the early detection of lack of response and opens for the possibility to switch to alternative treatment. It also offers the opportunity to study eventual gains from treatment intensification or addition of drugs during ongoing therapy.

The use of preoperative treatment in locally advanced and inflammatory breast cancer is standard of practice today. And although none of the randomized studies on operable breast cancer has shown survival benefit, the concept of preoperative treatment is of great interest for further research with regard to the detection and evaluation of new predictive factors and treatment concepts, including dose administration and development of new promising regimens (Mano et al 2004). The principle of *in vivo* testing of treatment at an early stage of the disease allows for estimation of response and, in case of no response, change of the therapeutic strategies. As a consequence, primary treatment could be a useful tool for personalized treatment. This, however, needs to be proven in prospective trials.

1.1.5 The search for new biological markers of response

The rapid development of techniques to map the human genome, the most recent advancement from microarray platforms to Next Generation Sequencing, together with publication of gene sequences online and access to large databases like the National Center for Biotechnology Informations' (NCBI) Human Genome Resources offers the chance to a rapprochement between laboratory and clinical sciences. Primary systemic treatment is an ultimate tool to identify biological characteristics in previously untreated tumors. Current problems in the treatment of breast cancer may be manageable, if biological material from both tumor and blood are repeatedly collected before, during and after treatment:

1. Tumor heterogeneity is the major challenge during treatment of breast cancer and may explain acquired resistance to treatment. Repeated mapping of differences within the same tumor during treatment using modern technology may enable the detection of biological markers allowing for the composition of drugs on an individual level.
2. Resistance to therapy. Data from neoadjuvant trials of chemotherapy have shown that a vast majority of tumors respond to chemotherapy, particularly in the HER2-positive subgroup in combination with monoclonal antibodies. However, tumors in pathologic complete response (pCR) are in the minority. Analyses of residual tumor tissue are an object of major interest for research on markers of response.

1.1.6 Study concept of the PREDIX platform

With the aim to improve treatment on an individual level, the PREDIX platform is designed as a compilation of randomized phase II studies based on functional "intrinsic subgroups" with the intention to direct treatment in relation to response:

1. Luminal A (ER+ and/or PR+ without node metastases);
2. Luminal $A_{\text{Npalp} \leq 40 \text{ years}}$ (ER+ and/or PR+ with node metastases)/Luminal B (ER+ and high proliferation);
3. HER2 amplified (includes both HR-positive (luminal B) and HR-negative tumors);
4. Triple-negative (none of the characteristics above).

The clinical management is accompanied by repeated tumor biopsies and blood samples. Besides mammography and ultrasound, functional imaging with PET-CT, confined to the breast and regional lymph nodes, is applied when available.

The goal is to cover the spectrum of different types of breast cancer, defined by functional biological characteristics rather than classical prognostic features, and treat the patients based on current knowledge of the biological properties.

Separate subprotocols are designed with the intention to explore the efficacy of compounds potentially related to molecular “intrinsic” subtypes (Parker, et al.). Depending on the research issue, the studies will be performed as randomized phase II trials. More specific features of each of the substudies are described separately.

The PREDIX LumB protocol describes a neoadjuvant randomized phase II trial comparing endocrine treatment combined with the cdk4/6 inhibitor palbociclib with chemotherapy using single drug paclitaxel.

1.2 Hormone receptor positive breast cancer

The present protocol covers slowly proliferating hormone receptor positive tumors which have different biological properties compared with those with high mitotic count. Data from clinical trials addressing preoperative endocrine treatment of these tumors is limited (Eiermann et al 2001; Smith et al 2005), possibly due to the low frequency of objective response reported from these trials and chemotherapy is at present the treatment of choice, even in slowly proliferating tumors.

Palbociclib is a selective inhibitor of the cyclin-dependent kinases CDK4 and 6. CDK4 activity is restricted to the G1-S phase and controlled by Cyclin D1, CDK6 has impact on the G1 phase progression and G1/S transition, controlled by Cyclin D2 (Rocca et al 2014). Palbociclib inhibits the CDK-regulated Rb phosphorylation at Ser^{780}, thereby preventing thymidine incorporation into the DNA. The inhibition has been reported as restricted to Rb positive cells (Fry et al 2004). In tamoxifen-resistant MCF7 cell lines, palbociclib was found to enhance sensitivity for tamoxifen (Finn et al 2009).

In a phase II randomized trial including postmenopausal patients with advanced HER2 negative hormone receptor positive breast cancer, letrozole in combination with 125 mg palbociclib was compared with letrozole and placebo. A final update (PALOMA-1) has been published recently (Finn RS et al 2015). The trial revealed a highly significant prolongation of the median PFS from 10.2 to 20.2 months in favor of the combined treatment (HR 0.49, 95 % CI 0.32-0.75, p=0.0004). Median overall survival (OS) was prolonged from 33.3 to 37.5 months in the combination arm, but the study was not powered to show a significant difference (HR 0.81, p=0.21).

A phase III comparison of palbociclib + fulvestrant with fulvestrant + placebo (PALOMA-3) in patients who had progressive disease on previous endocrine treatment showed a median progression-free survival of 9.5 months in the palbociclib/fulvestrant arm, compared with 4.6 months in the fulvestrant/placebo arm (Cristofanilli M et al 2016). Based on the results from these trials, palbociclib (IBRANCE®), in combination with letrozole, has been approved by the FDA and EMA for first-line treatment of metastatic breast cancer in postmenopausal women and, based on data from PALOMA-3, as second-line treatment in combination with fulvestrant after progression on previous endocrine therapy. Reported side effects due to palbociclib have been moderate: grade 3 neutropenia occurred in 12 % of patients, anemia in 7 %. Non-hematological toxicity included fatigue, nausea and diarrhea as the most commonly reported adverse events (Flaherty et al 2012). For more details, see ch. 4.8.

References

Cristofanilli M, Turner NC, Bondarenko I, et al. Fulvestrant plus palbociclib versus fulvestrant plus placebo for treatment of hormone-receptor-positive, HER2-negative metastatic breast cancer that progressed on previous endocrine therapy (PALOMA-3): final analysis of the multicentre, double-blind, phase 3 randomised controlled trial. Lancet Oncol. 2016 Apr;17(4):425-39

Early Breast Cancer Trialists' Collaborative Group (EBCTCG). Relevance of breast cancer hormone receptors and other factors to the effi cacy of adjuvant tamoxifen: patient-level meta-analysis of randomised trials. Lancet 2011;378:771–84

Early Breast Cancer Trialists' Collaborative Group (EBCTCG). Comparisons between different polychemotherapy regimens for early breast cancer: meta-analyses of long-term outcome among 100 000 women in 123 randomised trials. Lancet 2012;379:432-44

Eiermann W, Paepke S, Appfelstaedt J, et al. Preoperative treatment of postmenopausal breast cancer patients with letrozole: A randomized double-blind multicenter study. Ann Oncol 2001;12(11):1527-32

Finn RS, Crown JP, Boer K, et al. Results of a randomized phase 2 study of PD 0332991, a cyclindependent kinase (CDK) 4/6 inhibitor, in combination with letrozole vs letrozole alone for first-line treatment of ER+/HER2-advanced breast cancer (BC). Annals of Oncology 2012;23, Suppl. 2

Finn RS, Crown JP, Lang I, et al. The cyclin-dependent kinase 4/6 inhibitor palbociclib in combination with letrozole versus letrozole alone as fi rst-line treatment of oestrogen receptor-positive, HER2-negative, advanced breast cancer (PALOMA-1/TRIO-18): a randomised phase 2 study. Lancet Oncol 2015; 16: 25–35

Flaherty KT, Lorusso PM, Demichele A, et al. Phase I, dose-escalation trial of the oral cyclin-dependent kinase 4/6 inhibitor PD 0332991, administered using a 21-day schedule in patients with advanced cancer. Clin Cancer Res 2012;18(2):568-76

Fry DW, Harvey PJ, Keller PR, et al. Specific inhibition of cyclin-dependent kinase 4/6 by PD 0332991 and associated antitumor activity in human tumor xenografts. Mol Cancer Ther 2004;3(11), 1427-1438

Higgins MJ, Liedke PER, Goss PE. Extended adjuvant endocrine therapy in hormone dependent breast cancer: The paradigm of the NCIC-CTG MA.17/BIG 1–97 trial. Crit Rev Onc Hem 2013;86:23–32

Ma CX, Gao F, Luo J, et al. NeoPalAna: Neoadjuvant palbociclib, a cyclin-dependent kinase 4/6 inhibitor, and anastrozole for clinical stage 2 or 3 estrogen receptor positive breast cancer. Clin Cancer Res. 2017 Mar 7. doi: 10.1158/1078-0432.CCR-16-3206. [Epub ahead of print]

Rocca A, Farolfi A, Bravaccini S, et al. Palbociclib (PD 0332991): targeting the cell cycle machinery in breast cancer. Expert Opin Pharmacother 2014;15(3):407-420

Smith IE, Dowsett M, Ebbs SR, et al. Neoadjuvant treatment of postmenopausal breast cancer with anastrozole, tamoxifen, or both in combination: the Immediate Preoperative Anastrozole, Tamoxifen, or Combined with Tamoxifen (IMPACT) multicenter double-blind randomized trial. J Clin Oncol. 2005;23(22):5108-16

von Minckwitz G, Blohmer JU, Costa SD. Response-guided neoadjuvant chemotherapy for breast cancer. J Clin Oncol 2013;31(29):3623-30

2 MANAGEMENT, AIMS AND OBJECTIVES

2.1 Management procedures

Before randomization, assignment for the PREDIX LumB trial is defined by immunohistochemistry presenting with expression of ER, PR and Ki67 as defined in ch. 3.1 p. 3.

All patients with unknown lymph node status are offered sentinel-node biopsy before start of therapy. This procedure must be performed at latest one week before start of treatment.

Before start, after 6, 12, 18 and 24 weeks of treatment, radiological assessments of tumor size are performed using mammography and ultrasound, or alternatively MRI breast. PET-CT, confined to the breast and regional lymph nodes, is performed before start and after 12 weeks, blood samples before start, after 6, 12, 18 and 24 weeks.

Patients are randomized to receive 12 weeks of *either* weekly treatment with paclitaxel *or* endocrine therapy together with palbociclib and are thereafter switched to the opposite treatment for further 12 weeks.

Physical examinations are performed before start and then four-weekly after weeks 4, 8, 12 (switch), 16, 20 and 24 weeks of treatment. If any evaluation reveals disease progression, individualized management in the patient's best interest must be considered, in which case surgery is the primary option.

2.2 Aims

1. Evaluation of the impact of response-guided targeted treatment on objective response.
2. Identificatication of tumor characteristics and treatment-related changes of tumor biology predictive of *long-term prognosis.*

2.3 Objectives

2.3.1 Primary endpoint

Absolute difference in clinical and radiological objective response after completion of the first 12-week period of primary medical treatment

2.3.2 Secondary endpoints

Pathological objective response to primary medical treatment.

Sequencing of chemotherapy *versus* endocrine treatment plus palbociclib

Morphological, functional and biological characteristics of tumors exposed to cytotoxic and targeted treatment of early breast cancer.

Event-free survival (EFS), invasive disease-free survival (IDFS), distant disease-free survival (DDFS), breast cancer-specific survival (BCSS) and overall survival (OS)

Safety

Quality of life

Frequency of breast-conserving surgery.

3 PATIENT SELECTION CRITERIA

3.1 Inclusion criteria

1. Written informed consent
2. Female or male patients with breast cancer confirmed by histology
3. Tumor and blood samples available. *Luminal type* confirmed by immunohistochemistry with ER ≥10 %, *not* HER2 3+/amplified
4. Age 35 years or older. Elderly patients in condition adequate for planned therapy
5. Primary breast cancer >20 mm in diameter *and/or* verified regional lymph node metastases
6. Adequate bone marrow, renal, hepatic and cardiac functions and no other uncontrolled medical or psychiatric disorders
7. ECOG performance status 0-1
8. Primary breast cancer as defined in p. 5 plus at most 2 morphologically characterized well-defined distant metastases accessible for stereotactic radiotherapy, provided that this treatment is available.

3.2 Exclusion criteria

1. Distant metastases, including node metastases in the contralateral thoracic region or in the mediastinum
2. Other malignancy diagnosed within the last five years, except for radically treated basal or squamous cell carcinoma of the skin or CIS of the cervix
3. Patients in child-bearing age without adequate contraception
4. Pregnancy or lactation
5. Uncontrolled hypertension, heart, liver, kidney related or other medical or psychiatric disorders.

4 STUDY TREATMENT

Patients are randomized to *either* weekly paclitaxel *or* endocrine treatment in combination with palbociclib. The drugs used for endocrine treatment in the present trial, tamoxifen, anastrozole, letrozol, exemestane and LHRH analogues are commercially available and delivered by the local pharmacy. Palbociclib 125 mg, 100 mg and 75 mg tablets are packed and provided by Pfizer Sweden AB and labelled by Kliniska Prövningar, Oriola, Sweden. Each bottle contains 21 tablets palbociclib. The drug will be supplied either in HDPE bottles or Aclar/foil blisters. The content of the labelling is in accordance with requirements set by the regulatory authorities.

4.1 Investigational drug

Substance (INN): Palbociclib
INN number: 9802

Pharmaceutical form: Tablets
Source: Pfizer Inc.
Unit strength: 125 mg standard dose, 100 mg, 75 mg
Posology: Once daily for 21 days, followed by 7 days rest
Route of administration: Oral

4.2 Comparative drugs

1.1.1 Endocrine drugs

Tamoxifen 20 mg, *or*
Anastrozole 1 mg, *or*
Exemestane 25 mg, *or*
Letrozole 2.5 mg
LHRH analogues, not specified
Source: Not specified
Posology: Once daily continuously
Route of administration: Oral

4.2.1 Paclitaxel

Pharmaceutical form: Concentrate for solution 6 mg/ml
Unit strength: 80 mg/m^2 BSA standard dose. 70-90 mg/m^2
Posology: One dose per 7 days, weekly administration
Route of administration: Intravenous

4.3 Treatment schedule and doses

Patients are randomized to *either* weekly treatment with paclitaxel *or* endocrine therapy in combination with the cdk 4/6 inhibitor palbociclib:

Dose regimen weeks 1 to 12 of treatment:

a. Arm A:

Weekly paclitaxel on days 1, 8, 15, 22 etc., starting dose 80 mg/m^2. Doses can thereafter be individually adjusted in the range between 70 and 90 mg/m^2 in relation to side effects. In case of no change or decrease of the tumor after six weeks, this treatment is continued for further six weeks. Upon progression (PD), treatment is prematurely switched to the 2nd sequence

or

b. Arm B:

Pre- or perimenopausal women and all men are treated with tamoxifen 20 mg, alternatively with an LHRH analogue in combination with an aromatase inhibitor (only women), postmenopausal women receive an aromatase inhibitor, either anastrozole 1 mg, exemestane 25 mg, or letrozole 2.5 mg (postmenopausal patients) in combination with the cdk 4/6 inhibitor palbociclib 125 mg orally days 1-21, followed by a 7-days rest period. Patients are instructed to take palbociclib in connection with a meal. No specific medication to prevent side effects is recommended. For drug interactions of palbociclib, see 4.9.

In cases with uncertain menopausal status (previous hysterectomy and equivocal gonadotropins), postmenopause age limit is defined as 55 years or older. Dose reduction of

palbociclib to 100 mg (step -1) or 75 mg (step -2) in relation to side effects. Upon progression (PD), treatment is prematurely switched to the 2nd sequence.

After the initial 12-week period, treatment is switched to the opposite regimen.

Dose regimen weeks 13 to 24 of treatment:

c. Arm A:

Treatment is switched to tamoxifen (pre- or perimenopausal patients, men), alternatively to an LHRH analogue in combination with an aromatase inhibitor (only women), or an aromatase inhibitor in combination with the cdk 4/6 inhibitor palbociclib 125 mg orally days 1-21, followed by a 7-days rest period. In cases with uncertain menopausal status (previous hysterectomy and equivocal gonadotropins), postmenopause age limit is defined as 55 years or older. Dose reduction of palbociclib to 100 mg (step -1) or 75 mg (step -2) in relation to side effects. The treatment is continued for totally 12 weeks, provided that re-evaluations during treatment do not indicate progression. A delay of the therapy for medical reasons causing prolongation of the treatment period is exception and must be motivated;

or

d. Arm B:

Treatment is switched to weekly paclitaxel on days 1, 8, 15, 22 etc., starting dose 80 mg/m^2. Doses can thereafter be individually adjusted in the range between 70 and 90 mg/m^2 in relation to side effects. In total, 12 treatments are given, provided that re-evaluations during treatment do not indicate progression. In case of delay of a treatment for medical reasons, the treatment period extends 12 weeks.

e. Radiological evaluations including mammography, ultrasound alt. MRI breast are performed before start and then after 6, 12, 18 and 24 weeks of preoperative treatment, PET-CT confined to the breast and regional lymph nodes are performed before start and after 12 weeks. Physical examinations are performed before start and then four-weekly after weeks 4, 8, 12, 16, 20 and 24 weeks of treatment. Note that postponement of the treatment also causes postponement of evaluations. Termination of the preoperative treatment is followed by surgery.

f. Switch between the different endocrine treatment alternatives is allowed in case of intolerable side effects or lack of response, but should be otherwise omitted.

g. Paclitaxel: In cases with repeated severe infusion-related reactions motivating discontinuation, treatment with paclitaxel can be replaced by nab-paclitaxel (Abraxane®), 125 mg/m^2 for the remaining treatment period. This change of drug should be used only as an exceptional measure and be reported as adverse event;

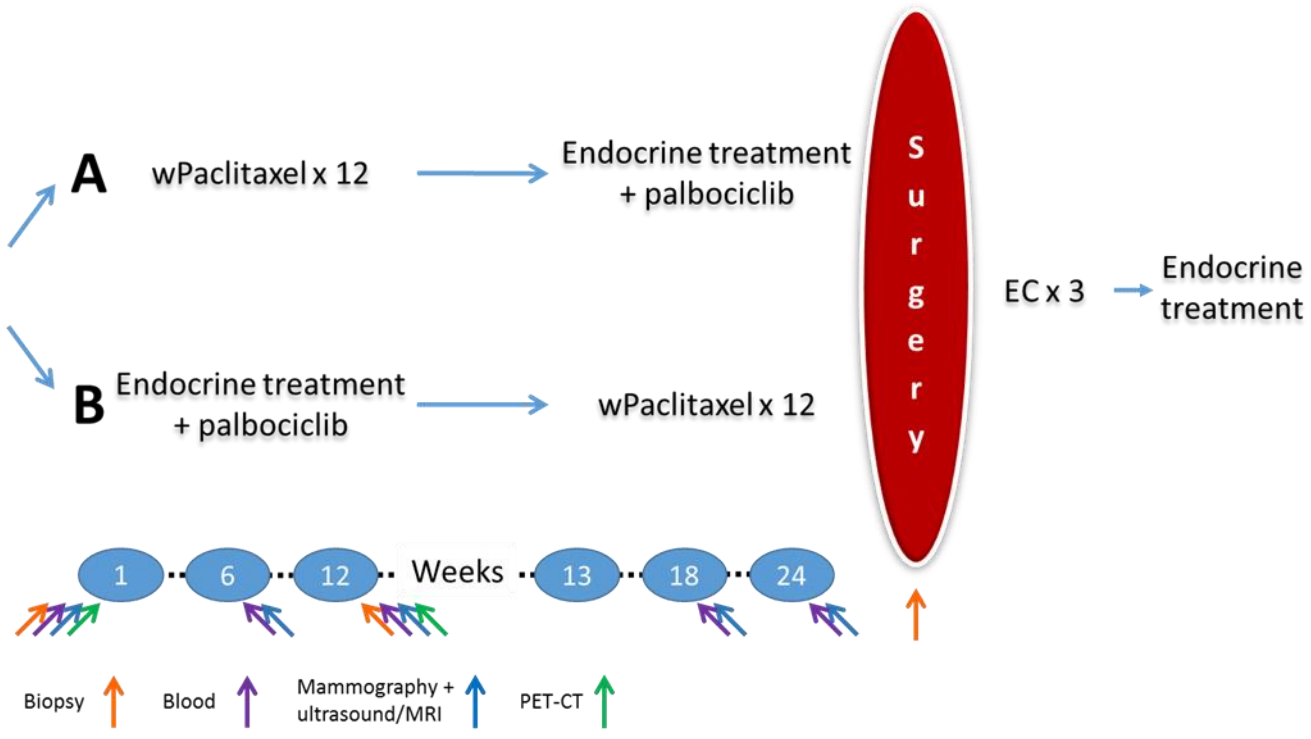

h. Upon progression (PD) during treatment, individualized management, preferentially surgery, is the primary option.

i. Core biopsies, blood samples and functional imaging procedures with PET-CT (when available, confined to the breast and regional lymph nodes) are performed as scheduled in the figure above.

j. After surgery, all patients are offered three courses of chemotherapy with a combination containing anthracycline. Standard treatment is the combination EC, with epirubicin 75-100 mg/m^2 and cyclophosphamide $\geq$500 mg/m^2.

k. All patients are recommended continuous endocrine therapy for at least 5 years.

4.4 Premedication

No specific premedication is required.

4.5 Precautions

Women of childbearing potential must have a negative pregnancy test prior to treatment with palbociclib. Female patients must be surgically sterile or postmenopausal, or they must agree to use effective contraceptive during the on-treatment period of the study and for at least 90 days after completion of treatment. Male patients must be surgically sterile or must agree to use effective contraception during the on-treatment period of the study and for at least 90 days after completion of treatment. The decision of effective contraception will be based on the judgment of the principal investigator or a designated associate.

In men: Testicular degeneration has been observed in animal studies, in certain species irreversible. The degeneration produced by palbociclib is consistent with CDK inhibition and alterations in cell cycle kinetics. Male patients should consider sperm preservation prior to beginning therapy with palbociclib.

4.6 Laboratory values before start of treatment

Laboratory values before start of treatment:

White blood count (WBC)
Neutrophil count (ANC)
Platelet count
Total bilirubin
S-ASAT
S-ALAT
S-ALP
Serum creatinine

4.7 Laboratory tests during treatment

Laboratory tests weekly for the *1st four weeks* of treatment with palbociclib:

White blood count (WBC) and
Neutrophil count (ANC)

Laboratory tests *after 4, 8, 12, 16, 20 and 24 weeks* of treatment:

White blood count (WBC)
Neutrophil count (ANC)
Platelet count
Total bilirubin
S-ASAT
S-ALAT
S-ALP
Serum creatinine

4.8 Toxicity of palbociclib

The following side effects have been reported from clinical trials with palbociclib when given alone:

- 30 % or more: decrease in neutrophils, fatigue;
- 10 to <30 %: decrease in hemoglobin, diarrhea, nausea, decrease in platelets, decrease in white blood cells, decreased appetite, constipation;
- 5 to <10 %: rash, vomiting, flatulence, hair loss, swelling of hands and feet, headache, nosebleed, muscle spasm, mucositis, joint pain, fever, dry mouth;
- side effects that occurred less frequently include: abdominal swelling, abdominal discomfort, abdominal pain, indigestion, pain, chills, shortness of breath, cough, runny nose, mouth/throat pain, back pain, flank (side) pain, muscle weakness, muscle pain, pain in hands and feet, itching, night sweats, dizziness, impaired sense of taste, decreased sense of touch or sensation, decreased weight, insomnia, hypotension, palpitations, blurred vision.

The following side effects have been reported in clinical trials with palbociclib when given together with hormonal therapy, letrozole, in patients with breast cancer:

- 30 % or more: decrease in neutrophils, decrease in white blood cells, fatigue, decrease in hemoglobin, nausea;

- 10 to <30 %: joint pain, diarrhea, hot flashes, back pain, hair loss, decreased appetite, decreases in platelets, vomiting, shortness of breath, constipation, headache, upper respiratory tract infection, cough, inflammation of the mouth, dizziness, weakness, common cold, pain in hands and feet, numbness and tingling in hands and feet, bone pain, musculoskeletal pain, influenza, rash, insomnia;
- 5 to <10 %: fever, edema of hands and feet, indigestion, nosebleed, mouth/throat pain, urinary tract infection, dry skin, mucositis, abdominal pain, toothache, impaired sense of taste, increases in blood liver markers such as alkaline phosphatase, alanine aminotransferase, and aspartate aminotransferase, depression, pain during urination, pain, blood clot in your lung, nail disorders, increase of creatinine, high blood pressure, conjunctivitis and increase in tear production.

4.9 Drug interactions of palbociclib

Since palbociclib is metabolized by CYP3A4, the following compounds should be avoided due to potential risks to modify the metabolism of palbociclib:

Inhibitors of CYP3A: ketoconazole, miconazole, itraconazole, posaconazole, clarithromycin, erythromycin, tilithromycin, nefazodone, diltiazem, verapamil, indinavir, saquinavir, ritonavir, nelfinavir, lopinavir, atazanavir, amprenavir, fosamprenavir, and grapefruit juice;

Inducers of CYP3A: phenobarbital, rifampin, phenytoin, carbamazepine, rifabutin, rifapentin, clevidipine, St. John's Wort ("Johannisört").

Interactions: Pharmacokinetic data from a study on healthy volunteers have shown palbociclib-midazolam drug-drug interaction. Midazolam ("Dormicum"), a benzodiazepine derivative for IV use, is a sensitive CYP3A4/5 probe substrate. When midazolam was co-administered with palbociclib, geometric mean Cmax and AUCinf values increased 1.37-and 1.61-fold. Midazolam and other compounds for which CYP3A-mediated metabolism constitutes the primary mechanism of clearance should therefore not be administered during daily administration of palbociclib.

4.10 Dose adjustments of palbociclib

Standard dose of palbociclib is 125 mg once daily for 3 weeks, followed by 1 week off treatment. Dose adjustments are performed in relation to hematological side effects, primarily decrease of ANC and/or platelets, or non-hematological events, particularly impairment of the liver function. Dose reduction of palbociclib by one or two dose levels is permitted depending on treatment related toxicity:

Standard dose:	125 mg
Step -1:	100 mg
Step -2:	75 mg

Dose adjustments are made according to the following guidelines:

Toxicity	Dose adjustments of palbociclib
**Uncomplicated Grade 3 neutropenia** *(ANC ≥0.5 - <1.0 x 10^9/L)*	• 1st occurrence: Hold drug. If ANC recovers (ANC ≥1.0 x 10^9/L) within 2 weeks, resume at same dose.<br>• If ANC takes longer than 2 weeks to recover (ANC ≥1.0 x 10^9/L), but within 3 weeks, then resume drug and decrease drug by 1 dose level.<br>• Recurrent uncomplicated Grade 3: Hold drug. If ANC recovers (ANC ≥1.0 x 10^9/L) within 2 weeks, resume drug and decrease drug by 1 dose level.
**Grade 3 neutropenia** *(ANC<1.0 x 10^9/L)* associated with a documented infection or fever ≥38.5°	• Hold drug. If ANC recovers (ANC ≥1.0 x 10^9/L) within 2 weeks, resume drug and decrease drug by 1 dose level.<br>• If ANC takes longer than 2 weeks to recover (ANC ≥1.0 x 10^9/L), but within 3 weeks, then resume drug and decrease drug by 2 dose levels.<br>• If these parameters have not been met after 3 weeks of dose interruption (including the scheduled 1 week off treatment), the patient should permanently discontinue palbociclib.
**Grade 4 neutropenia** (*ANC <0.5 x 10^9/L)*	• First occurrence: Hold drug. Resume once ANC ≥1.0 x 10^9/L and decrease dose by 1 dose level.<br>• Recurrent Grade 4 neutropenia: Hold drug. Resume once ANC ≥1.0 x 10^9/L and decrease dose by an additional dose level.
**Grade 3 or 4 thrombocytopenia** (*platelet count <50 x 10^9/L*)	• 1st occurrence: Hold drug until plt ≥100 x 10^9/L, then resume drug and decrease by 1 dose level.<br>• Recurrent Grade 3 thrombocytopenia: hold drug until plt ≥100 x 10^9/L, then decrease drug by an additional dose level.
**Grade ≥3 non-hematologic toxicity** *(including, nausea, vomiting, diarrhea, and hypertension only if persisting despite optimal medical treatment)*	• 1st occurrence: Hold drug until toxicity decreases to ≤ Grade 1 or to baseline, then resume drug and decrease by 1dose level.<br>• If toxicity takes longer than 2 weeks to recover to ≤ Grade 1, but within 3 weeks, then resume drug and decrease drug by 2 dose levels.<br>• Recurrent toxicity: Hold drug until toxicity decreases to ≤ Grade 1 or to baseline, then decrease drug by an additional dose level.
**Grade 2 non-hematologic toxicity** persisting despite optimal medical treatment, deemed unacceptable in the investigator's judgment, and lasting at least 2 weeks	• 1st occurrence: Hold drug until toxicity decreases to ≤ Grade 1 or to baseline, then resume drug at same dose level.<br>• Recurrent toxicity: Hold drug until toxicity decreases to ≤ Grade 1 or to baseline, then resume drug and decrease by 1 dose level.
**Liver function tests** Concurrent >3 x ULN ALT and 2 x ULN total bilirubin (Hy's Law)	Discontinue palbociclib permanently.

5 TREATMENT EVALUATION SCHEDULES

5.1 Response evaluation

Response evaluations include imaging with mammography, ultrasound, eventually MRI, and PET-CT of the breast and regional lymph nodes, where available. Clinical measurements with calliper, in non-measurable tumors clinical signs of treatment response.

In patients without palpable axillary lymph node metastases, sentinel node biopsy (SNB) is recommended standard procedure, but must not be performed closer than 7 days prior to start of therapy.

5.1.1 Before start of treatment

The following procedures are performed prior to start of therapy:

Imaging procedures including mammography, ultrasound, alternatively MRI breast, and PET-CT (breast and regional lymph nodes).

In connection with mammography before start of the treatment, the tumor borders must be marked, either with carbon suspension or similar alternatives.

Biopsies are performed using 14 Gauge-needle, for details see the ”Manual for handling of biological material in the PREDIX platform”, hereinafter referred to as “Manual”:

1. Two core biopsies are fixed in formalin for between 24 and 48 hours and then embedded in paraffin according to standard procedures.
2. Three to four, if possible more, core biopsies are immediately snap frozen, except for one of the biopsies prepared for proteomics which is pretreated with protease inhibitor. The samples must be handled with sterile gloves to avoid contamination with RNAse.

   For details, see the ”Manual”.

Blood samples include whole blood, EDTA plasma and serum collected prior to start of therapy. For details see the ”Manual”.

5.1.2 During treatment

Imaging procedures mammography, ultrasound, if necessary MRI breast are repeated after 6, 12, 18 and 24 weeks of treatment, PET-CT of the breast and regional lymph nodes after 12 weeks. Imaging must be performed *before* the biopsy after week 12.

Biopsies with 14 G-needle are repeated after 12 weeks of treatment according to the same arrangement as described above.

Blood samples (study) including whole blood, EDTA plasma and serum are collected after 6, 12, 18 and 24 weeks (± 2 days) of treatment.

Routine blood tests (WBC, ANC) weekly x 4 after start of palbociclib and then, together with other blood tests as described in ch. 4.7 every four weeks of treatment.

Physical examinations take place every 4 weeks: 4, 8, 12, 16, 20 and 24.

Patients who due to disease progression terminate study treatment prematurely are reported similar to those who complete the treatment as scheduled.

5.1.3 After treatment and during follow-up

Tumor tissue from the *surgical specimen* is obtained by core biopsies with a 14 G-needle according to the same arrangement as described above. If the post-treatment evaluation showed no palpable lump and radiological signs of complete response, the biopsies are taken from the area within the boundaries marked with carbon suspension or other alternatives before start of the treatment.

Blood samples are taken in connection with the End-of-Treatment visit three months after surgery and at annual follow-up visits for the first five years. Plasma samples are stored in separate amounts as described in the ”Manual”.

Annual mammography controls.

In the case of recurrence, see chapter 19.

5.2 Safety evaluation

Safety will be assessed by use of clinical Adverse Events and Common Toxicity Criteria (CTC) laboratory and non-laboratory toxicities. NCI Common Terminology Criteria for Adverse Events version 4.0 (CTCAE) will be used. This version is available at http://ctep.cancer.gov/reporting/ctc.html.

At the time of enrolment, a baseline status is documented to note the occurrence and nature of each patient’s medical conditions.

5.2.1 Adverse event reporting

During treatment, CTC toxicity rating will be performed in connection with physical examination visits for any adverse event that has been experienced during the previous cycle. Adverse event reporting is documented in the clinical Case Report Form (CRF). AE/SAE reporting covers the period between start of the first cycle until 30 days after termination of study treatment and until resolution.

Patients who due to severe side effects terminate study treatment prematurely are followed according to the same schedule as patients who completed the treatment.

Special attention is paid to the following adverse reactions:

1. *Elevation of liver transaminases.* To avoid drug-induced liver injury (DILI), serum levels of the transaminases ALP, ASAT and ALAT must not exceed >3 x ULN, and serum bilirubin >2 x ULN. In case of elevation of either one or both transaminases in combination with elevation of bilirubin, this must be reported as SAE (Hy’s Law case).
2. *Infusion-related and hypersensitivity reactions.* All reactions, including transient flushing or rash, or drug fever have to be reported.
3. *Hematologic reactions.* Delayed recovery of ANC. Reporting as SAE limited to grade 3/4 toxicity.

AE/SAE reporting from 1st dose of palbociclib until 28 calendar days after last administration and until resolution.

5.2.1.1 Serious Adverse Event (SAE):

Study site personnel must report any event during the study that results in the following outcomes:

1. Death

2. Hospitalization initiated or prolonged due to an adverse event, except for prophylactic hospitalization to prevent repetition of a previous toxic reaction
3. A life threatening experience
4. Severe or permanent disability
5. Congenital anomaly, birth defect, neonatal death
6. Any other event considered to be an important medical event.

Suspected Unexpected Serious Adverse Reaction (SUSAR):

Per definition an adverse reaction that is both unexpected (not consistent with the applicable product information) and also meets the definition of a Serious Adverse Event must be reported likewise.

Not to be reported as SAE:

Progression or relapse of breast cancer or hospitalisation due to disease progression.
Hospitalization for elective surgery or other preplanned treatment.

An SAE must be reported by the study personnel immediately upon awareness if the SAE is fatal or life threatening regardless of the extent of available information, within 24 h of first awareness if the SAE is not fatal or life threatening. The report is via fax sent to the CTO, KPE, Radiumhemmet, fax +46-(0)8 30 69 89 using the study specific SAE form (see Appendix 1).

Toxicity which possibly may be related to the study treatment will be followed and reported during the entire follow-up period until resolution.

5.2.1.2 *Suspected Unexpected Serious Adverse Reaction (SUSAR):*

Suspected unexpected serious adverse reaction (SUSAR) is any serious event that is suspected to be connected to the study treatment and not described in the Summary of Product Characteristics (SPC).

The sponsor will decide if the Serious Adverse Drug Reaction (SADR) is a SUSAR or not.

After the sponsor's decision, the investigator must report the SUSAR to the National Agencies (EU/EES) in every country. If a SUSAR is lethal or life threatening, this must be reported within 7 days and any relevant complementary information within another 8 days. All other SUSARs shall be reported within 15 days. Suspect Adverse Reaction Report Form (CIOMS Form I) will be used for submitting reports to the National Agencies for registration in EudraVigilance. The sponsor will report to the Swedish Medical Product Agency and inform all investigators of SUSARs as they occur. A copy of all SUSARs must instantly be sent to Pfizer Sweden AB by the sponsor.

A patient will be withdrawn immediately if:

1 she or he withdraws her/his informed consent at her/his own request

2 unacceptable toxicity occurs, not manageable by symptomatic therapy, dose delay or dose modification

3 she or he does not comply with the instructions given by the study personnel

4 tumor progression is stated

5 pregnancy occurs

6 the medication has to be discontinued for medical reasons, including severe side effects, other disease, need of other cancer treatment.

Patients who have been withdrawn will continue to be tracked and assessed (unless the patient explicitly declines).

5.2.1.3 Safety reporting of exposure to palbociclib in relation to Pfizer

The following events during ongoing or recently terminated treatment with palbociclib have to be reported irrespective of an SAE:

1. Occupational exposure. Any possible occupational contact with the drug may primarily include nurses and pharmacy staff who come in direct contact with the drug
2. Exposure during breastfeeding. A child being exposed to palbociclib through breast milk
3. Exposure during pregnancy. A fetus exposed to palbociclib through the mother who is on treatment with the rug; a male patient exposed to the drug during the time of conception.

All cases of exposure have to be reported to the CTO with a description of the event, time and duration of exposure. The event will be reported to Pfizer according to the manual

Investigator-Initiated Research (IIR) Studies with Pfizer Products. Safety Reporting Reference Manual (For Interventional Clinical Studies in all Therapeutic Areas Including Oncology), version 5.2: November 2013.

5.3 Treatment after completion of preoperative therapy

Surgery:

Surgery, breast-conserving or mastectomy w/o reconstruction is performed approximately 2 weeks after termination of the medical treatment. This applies also to patients who terminated study treatment and received alternative treatment due to toxicity.

In case of absence of lymph node metastases confirmed through SNB before start of treatment, further axillary surgery in connection with breast surgery can be omitted. Micro metastases should be followed by repeated SNB, macro metastases by axillary clearance.

Adjuvant medical treatment:

Postoperative adjuvant treatment includes endocrine treatment for at least five years for all patients with hormone receptor positive tumors.

Radiotherapy:

Postoperative radiotherapy should be initiated within six weeks after surgery.

Recommendations:

<table>
<tr><th rowspan="2"></th><th colspan="2">Node negative</th><th>Node positive</th></tr>
<tr><th>T1-2</th><th>T3-4</th><th>All T</th></tr>
<tr><td>Breast-conserving surgery</td><td>Breast only</td><td rowspan="2">Breast + regional nodes</td><td>Breast + regional nodes</td></tr>
<tr><td>Modified radical mastectomy</td><td>No therapy</td><td>Thoracic wall + regional nodes</td></tr>
</table>

5.4 End-of-treatment/Follow-up

Clinical follow-up visits including physical examination three months after surgery, then annually for five years after surgery. Thereafter follow-up according to local routine with annual reports regarding ongoing adjuvant endocrine treatment, date and site(s) of recurrence, and date and cause of death up to ten years after surgery.

Imaging procedures including bilateral mammography and, at the radiologists discretion, ultrasound alt. MRI breast in connection with annual follow-up visits. CT scan and bone scan when motivated due to signs and symptoms.

**Assessment**	**3 months after surgery**	**Years 1–5 after surgery**	**Years 6–10 after surgery**
Physical examination	●	●	
Hemoglobin, RBC, WBC, Neutrophils, Platelets, serum chemistry	●	●	
Bilat. mammography, ultrasound/MRI		●	
Blood samples incl. plasma and serum		●	
Reporting ongoing adjuvant treatment	●	●	●
Local relapse, distant recurrence, death since previous visit, **see chapter 19**	●	●	●
HRQoL	●	●	
Toxicity	●	●	

6 RESPONSE MEASURES

6.1 Measurable disease by radiology

Response criteria according to RECIST (Eisenhauer et al 2009):

Complete response (CR): Disappearance of all clinical evidence of tumor (target and non-target lesions), determined by two observations not less than four weeks apart.

Partial response (PR): A ≥30 % decrease of the largest diameter determined by two observations not less than four weeks apart. No unequivocal increase in the size of non-target lesions or the appearance of new lesions may occur.

Stable disease (SD): Steady state of response (i.e. not PR and not PD) of at least two consecutive observations There must be no appearance of new lesions for this category.

Progressive disease (PD): A ≥20 % increase of the largest diameter or unequivocal progression of non-target lesions. Appearance of new lesions will also constitute progressive disease.

Besides changes of tumor size, indicators of shape and structure of the tumor, surrounding tissue and axillary lymph nodes will be followed during treatment, see APPENDIX

6.2 Clinical examination and criteria for measurement of clinical response

The tumor is described by

1. Localization in the breast in lying position, by quadrant(s), clockwise, distance from mamilla.
2. One-dimensional size by largest diameter.
3. Lymph node metastases in the axilla or the infra- or supraclavicular fossa by largest diameter.

Clinical assessment of the size of palpable tumor(s) is performed before start and then weeks 4, 8, 12, 16, 20 and 24. Measurable lesions in the breast and/or regional lymph node sites will be recorded.

Complete response (CR): Disappearance of all clinical evidence of tumor. Note that clinical complete response must be confirmed by mammography and ultrasound before therapy decisions are made.

Partial response (PR): ≥30 % decrease of the largest diameter of the tumor lesion.

Stable disease (SD): Changes of tumor size within the range of less than 30 % decrease and less than 20 % increase of tumor size between two observations.

Progressive disease (PD): ≥20 % increase of the largest diameter or unequivocal progression of non-measurable lesions. Appearance of new lesions will always constitute progressive disease, if verified by morphology and radiology.

In inflammatory breast cancer (breast enlargement, erythema over at least one-third of the breast, oedema and warmth of the skin) the clinical status at start and after cycles 2, 4 and 6 is documented by photography in presence of a measuring tape.

6.3 Pathologic evaluation of tumor response

Johan Hartman

Yet, there is no standard method to evaluate tumor response histologically. Several evaluation systems have been published (Chevallier et al 1993; Sataloff et al 1995). The most recent grading system has been proposed by Miller and Payne, in which cellularity and dispersion of the tumor cells after chemotherapy are estimated semi quantitatively (Ogston et al 2003). Since this grading has been prospectively studied in a consecutive series of patients it will be used in the trial.

In a trial comparing histological changes due to endocrine *versus* chemotherapy, the authors described changes in cellularity and appearance of "scattered scarring" as sign of treatment response (Thomas et al 2007). The architecture of scarring after chemotherapy was different compared with that due to treatment with an aromatase inhibitor.

The following tumor response grading system will be used to describe changes related to the treatment in comparison with pre-treatment histology (Miller and Payne grading system):

Grade 1: No change or some alteration to individual malignant cells but no reduction in overall cellularity.

Grade 2: A minor loss of tumor cells but overall cellularity still high; up to 30 % loss.

Grade 3: Between an estimated 30 % and 90 % reduction in tumor cells.

Grade 4: A marked disappearance of tumor cells such that only small clusters or widely dispersed individual cells remain; more than 90 % loss of tumor cells.

Grade 5: No malignant cells identifiable in sections from the site of the tumor; only vascular fibroelastotic stroma remains often containing macrophages. However, *ductal carcinoma in situ* (DCIS) may be present.

In addition, the following features which are frequently observed after chemotherapy are described (Rosen 1997):

* Fibrosis
* Stromal oedema
* Vascularity
* Lymphocyte infiltration

These features will be classified according to a semiquantitative grading scale: 1: none or low, 2: moderate, 3: high presence.

Definition of pathologic complete response (pCR) after neoadjuvant therapy in the PREDIX platform:

Absence of invasive carcinoma in the breast and axillary lymph nodes (ypT0/is ypN0). Remaining DCIS is accepted.

6.4 References

Chevallier B, Roche H, Olivier JP, et al. Inflammatory breast cancer: Pilot study of intensive induction chemotherapy (FEC-HD) results in a high histological response rate. Am J Clin Oncol 2993;16:223-228

Eisenhauer EA, Therasse P, Bogaerts J, et al. New response evaluation criteria in solid tumours: Revised RECIST guideline (version 1.1). Eur J Cancer 2009;45:228–47

Elston CW, Ellis IO. Pathologic prognostic factors in breast cancer. I. The value of histological grade in breast cancer: experience from a large study with long-term follow-up. Histopathology. 1991;19(5):403-410

Ogston KN, Miller ID, Payne S, et al. A new histological grading system to assess response of breast cancers to primary chemotherapy: prognostic significance and survival. Breast 2003;12(5):320–327

Rosen's Breast Pathology. 1st ed. Philadelphia, Pa: Lippincott Williams & Wilkins;1997:782-785

Sataloff DM, Mason BA, Prestipino AJ, et al. Pathologic response to induction chemotherapy in locally advanced carcinoma of the breast: A determinant of outcome. J Am Coll Surg 1995;180:297-306

Thomas JSJ, Julian HS, Green RV, et al. Histopathology of breast carcinoma following neoadjuvant systemic therapy: a common association between letrozole therapy and central scarring. Histopathology 2007;51(2):219–226

7 RADIOLOGICAL EVALUATION

7.1 Mammography and conventional ultrasound

Edward Azavedo

7.1.1 Background

Mammography can be used to evaluate the efficiency of preoperative hormonal and/or chemotherapy treatment of breast cancer patients. Decrease in tumor size and changes in tumor density are the two parameters mostly used. However, changes in distortion within the tumor and surrounding tissue and increase or decrease in the number of micro calcifications are also important parameters. In some cases, treatment causes decrease in density and distortion also in the tissue surrounding the tumor.

Lymph nodes in the axilla are more difficult to evaluate. The number of lymph nodes examined can differ from one examination to another just because of small changes in positioning of the breast during mammography. Anyhow, size and shape of examined lymph nodes, general density and if the hilus of the lymph node is radiolucent or dense can be used to estimate if metastases are present or not. Irregular borders of the node, particularly with spiculations, correspond to periglandular growth of tumor cells.

Ultrasound has proven to be the first best adjunct method to supplement mammography. This technique has a higher sensitivity than mammography in dense breasts where masses could be masked by the overall parenchymal density. Besides the possibility of using the method to demonstrate the presence of a tumor, this technology also gives information about tumor size, infiltration in the surrounding tissue, presence of multifocality that may not be appreciated by mammography due to the masking effect in dense breasts, vascular texture etc. Changes in the echo pattern can demonstrate changes in a tumor mass corresponding to therapy response.

7.1.2 Methods

Mammography. Each breast will be examined according to the three standard views, medio-lateral-oblique (MLO), cranio-caudal (CC) and latero-medial (LM). Special views like coned-down and magnifications views are sometimes helpful. Follow-ups during treatment will always include all three standard views of the ipsilateral breast despite short interval between examinations. In most cases, special views are not necessary. Mammographies include the contralateral breast for studies of breast density, see ch. 8.

Diagnostic ultrasound. Conventional ultrasound evaluation using a high frequency probe (>10 MHz) depicting tumor size and its infiltration in the surround tissue will be used. Besides this other parameters that modern technology shows will be used. These will be vascular texture with Colour Doppler Elastography, 3-D volume measurement of a tumor that will be especially useful to prove any change in the tumor volume, and the presence or disappearance of multifocality.

Response evaluation:

Tumor

* tumor size in two dimensions
* tumor density (1-3)
* homogenous or irregular formation of density
* growth pattern
* distortion
* calcifications

* multifocality
* vascularity

Surrounding tissue

* density (1-3)
* distortion
* calcifications

Lymph nodes in the axilla

* size
* density in the periphery (1-3)
* density in the hilus (1-3)
* regular or irregular borders

7.2 Positron Emission Tomography (PET-CT)

Per Grybäck

7.2.1 Background

Positron Emission Tomography (PET) has rapidly emerged as a powerful tool for tumor detection and evaluation. It reflects the *relative* metabolism of the injected tracer in tumor *versus* normal tissue at the moment of registration in the PET camera. Compared to ('conventional') single photon imaging, PET gives a higher spatial resolution and allows accurate quantification of radioactivity ('uptake'). PET also utilizes the 'bioisotopes', carbon-11 [^{11}C], oxygen-15 [^{15}O] and nitrogen-13 [^{13}N], which all have favourable biochemical properties. Since a few years, combined cameras for simultaneous high quality PET- and CT-examinations are available. These have been shown to add significant synergistic diagnostic information. The intensity of metabolism is localized anatomically - hence the term 'anatometabolic imaging' for combined PET-CT scanning. The choice of tracer depends on the target. The tracer is labelled with a positron emitting radionucleid, which, after colliding with electrons at the site of uptake, decays with an energy of 510 KeV.

For tumor imaging, the by far most frequently used radiopharmaceutical is deoxyglucose, labelled with ^{18}Fluor, [^{18}F]-fluorodexyglucose (FDG), which has a half-life of 110 minutes. Mechanisms for cellular deoxyglucose uptake are similar to those for glucose uptake. After phosphorylation, the tracer is trapped in the cell, and uptake and metabolism can be visualised. Enhanced energy metabolism is a common feature in malignant tumors, and, although not a specific marker for malignant tissue, [^{18}F]-fluorodexyglucose (FDG) has been shown as a reliable tracer of malignant tissue.

Generally, PET is superior in detecting disease spread than conventional methods are, but is hampered by resolution around 6-10 mm, varying due to dependence on the relative metabolism in the area studied. For routine staging, no evidence is at hand that PET is superior, except for locally advanced tumors where distant metastases are revealed to a higher extent than with conventional methods. In cases of suspected relapse, PET is clearly superior to conventional imaging in distinguishing post-therapeutic changes (after surgery and/or radiotherapy) from those caused by tumor relapse.

Tracers for the visualisation of aminoacid uptake, protein synthesis (labelled methionine and tyrosine), and proliferation, (labelled thymidine), among others, are at hand, but infrequently available at institutions with PET facilities. [^{18}F]-fluoro-L-thymidine (FLT) is a tracer that recently also has been used for tumor imaging. FLT is a thymidine molecule tagged with ^{18}F. Thymidine labelled by tritium ([^{3}H]-thymidine) has been used since many years for *in vitro* assessment of DNA-

synthesis, and FLT is regarded as a surrogate marker of DNA-synthesis and, hence, of cell division (proliferation).

[^{18}F]-fluorodexyglucose (FDG): PET with FDG constitutes the absolute majority of PET studies worldwide, as it is relatively easy to produce to a reasonable cost. Due to the half-life of 110 minutes of ^{18}F it can be transported from source (cyclotron unit) to user (PET center).

The tumor metabolism can be evaluated either by inspection (visual evaluation) or semi-quantitatively (Standardized Uptake Value - SUV) or quantitatively (Metabolic Rate - MR). Early metabolic response, i.e. a decrease of hypermetabolism preceding the tumor shrinkage one to two weeks after start of chemotherapy has in several clinical studies been shown as a predictor of remission and survival. The most convincing results have been published for Hodgkins lymphoma (Hutchings et al 2006). In breast cancer, some evidence in terms of prediction of response to therapy by sequential PET has been observed after one or two cycles of chemotherapy (Dose Schwarz et al 2005, Rousseau et al 2006).

Since FDG is a commonly used PET tracer, the evaluation of its potential in treatment prediction is of interest.

7.2.2 Methods

[^{18}F]-fluorodexyglucose (FDG): After injection of FDG, with the patient in a semi-fasted condition, i.e. no glucose containing liquids or food for 4 - 6 hours before injection, rest is required for about 60 minutes. The slope of FDG uptake rises faster in neoplastic tissue and continues for a couple of hours whereas a steady state in the normal tissue is attained after 40 minutes. This relative difference is enough for visualising the enhanced metabolism in tumors. Rest is required to avoid muscular FDG uptake, blurring the interpretation of the scan.

Response evaluation: PET is combined with low dose CT as anatomical guide without need of contrast agent and will be confined to the breast and regional lymph nodes. Evaluation of the PET-examinations will be made by calculating Standardized Uptake Values (SUV's) of the tumor in the digitized images. This represents absolute activity (uptake) which is possible to achieve after calibration of the camera sensitivity and correction for attenuation and scattering of photons. It is the regularly used method for quantification of uptake at PET-examinations. SUV's are calculated as mean values (SUV_{mean}) of a large portion of the tumor as well as the maximal uptake (SUV_{max}) of the tumor. The change of SUV's between the consecutive examinations in the patient will be related to the conventional methods for evaluation of neoadjuvant chemotherapy (physical examination and mammography) and histology of the resected tumor. By this, the aim is to define a cut-off level of uptake reduction in retrospect that early predicts an adequate therapy response. Recently, criteria for the evaluation of PET have been suggested, the Positron Emission Response Criteria In Solid Tumors (PERCIST version 1.01). These criteria propose measurement of the lean body mass corrected SUV, SUL (Standardized Uptake Lesion) peak and selection of the hottest tumor focus (Wahl et al 2009).

7.3 References

Positron Emission Tomography (PET-CT):

Dose Schwarz J, Bader M, Jenicke L, et al. Early prediction of response to chemotherapy in metastatic breast cancer using sequential ^{18}F-FDG PET. J Nucl Med 2005;46:1144–1150

Hutchings M, Loft A, Hansen M, et al. FDG-PET after two cycles of chemotherapy predicts treatment failure and progression-free survival in Hodgkin lymphoma. Blood 2006;107:52-59

Rousseau C, Devillers A, Sagan C, et al. Monitoring of early response to neoadjuvant chemotherapy in stage II and III breast cancer by [^{18}F]Fluorodeoxyglucose Positron Emission Tomography. J Clin Oncol 24:5366-5372

Wahl RL, Jacene H, Kasamon Y and Lodge MA. From RECIST to PERCIST: evolving considerations for PET Response Criteria in Solid Tumors. J Nucl Med 2009;50, Suppl 1 122S-150S

8 MAMMOGRAPHIC DENSITY

Louise Eriksson

8.1 Background

Mammographic density is a well-established risk factor of breast cancer (McCormack et al 2006). It reflects the tissue composition of the breast; the epithelium and fibrous tissue are radiodense and appear white on a mammogram whereas the fatty tissue is radiolucent and appears black. Consequently, larger amounts of fibroglandular tissue will lead to higher mammographic density and vice versa.

Mammographic density is a modifiable trait and may be reduced by e.g. tamoxifen. Notably, the reduction in density associated with tamoxifen has been shown to decrease breast cancer risk (Cuzick et al 2011). Two studies have investigated whether density may have a similar predictive value of tamoxifen treatment in the adjuvant setting (Kim et al 2012, Li et al 2013), one of them also including aromatase inhibitors (Kim et al 2012). Kim et al found that recurrences were fewer in women whose density decreased during treatment with tamoxifen or aromatase inhibitors. Li et al reported that women, whose density decreased as a result of adjuvant tamoxifen treatment, had an improved long-term survival. Thus, mammographic density could possibly be used as an indicator of early response to adjuvant hormonal treatment. However, the results above need verification. Furthermore, whether mammographic density could be used as an indicator of early response to other forms of adjuvant/neoadjuvant treatment is unknown.

8.2 Methods

Mammographic density is highly correlated between the two breasts and also between different views (Byng et al 1996). In the present trial, density of the contralateral breast will be followed to avoid any influence of the tumor on the measurements. Hence, patients with bilateral breast cancer will be excluded for this part of the study. Although both the cranio-caudal (CC) and mediolateral oblique (MLO) views can be used in density assessment, we will primarily use the MLO view. All density measurements will be carried out using an automated thresholding method previously described (Li et al 2012). This method automatically gives estimates on the absolute dense area, the absolute non-dense area, and percent mammographic density (the absolute dense area divided by the total breast area).

Response evaluation: To measure change in mammographic density, we will compare mammographic density at baseline, i.e. the mammography carried out before start of neoadjuvant treatment, and during the treatment according to the schedule and in connection with yearly follow-up controls after termination of the primary treatment. It is not known within which time frame any possible effects of treatment on mammographic density will be seen. However, increases in density caused by hormone-replacement therapy may already disappear within three weeks after cessation

(Colacurci et al 2001). Hence we believe that the length of the follow-up period will be sufficient in capturing changes in density.

Statistical methods: Kaplan-Meier curves and the Cox proportional hazards model will be used to study whether change in mammographic density is associated with survival, stratified by the different treatment arms.

8.3 References

Byng JW, Boyd NF, Little L, et al. Symmetry of projection in the quantitative analysis of mammographic images. European journal of cancer prevention: the official journal of the European Cancer Prevention Organisation 1996, 5(5):319-327

Colacurci N, Fornaro F, De Franciscis P, et al. Effects of different types of hormone replacement therapy on mammographic density. Maturitas 2001, 40(2):159-164

Cuzick J, Warwick J, Pinney E, et al. Tamoxifen-induced reduction in mammographic density and breast cancer risk reduction: a nested case-control study. J Natl Cancer Inst 2011, 103(9):744-752

Kim J, Han W, Moon HG, et al. Breast density change as a predictive surrogate for response to adjuvant endocrine therapy in hormone receptor positive breast cancer. Breast Cancer Res 2012, 14(4):R102

Li J, Szekely L, Eriksson L, et al. High-throughput mammographic density measurement: a tool for risk prediction of breast cancer. Breast Cancer Res 2012, 14(4):R114

Li J, Humphreys K, Eriksson L, et al. Mammographic density reduction is a prognostic marker of response to adjuvant tamoxifen therapy in postmenopausal patients with breast cancer. J Clin Oncol 2013, 31(18):2249-2256

McCormack VA, dos Santos Silva I. Breast density and parenchymal patterns as markers of breast cancer risk: a meta-analysis. Cancer Epidemiol Biomarkers Prev 2006, 15(6):1159-1169

9 QUALITY OF LIFE ASSESSMENT

Yvonne Brandberg

9.1 Background

One of the secondary aims of this study is to compare patients' health-related quality of life (HRQoL) between the treatment arms during the course of treatment and at long-term follow-up. To our knowledge, HRQoL data specific for the neoadjuvant setting in breast cancer have not been published. In the neoadjuvant situation, patients will be faced with the efficacy of the treatment on the tumor. This information might affect HRQoL favorable for those who respond and negatively for those who do not. In addition, differences in HRQoL between the treatment regimens will be evaluated on both short- and long-terms. The information gained can be used to improve patient information in a situation when the patient must take a treatment decision. Assessment during follow-up is also important in order to evaluate the long-term effects of the various treatments.

9.2 Methods

9.2.1 Patients

All patients included in the PREDIX LumB trial will be asked to participate in the HRQoL study.

9.2.2 Points of assessment

HRQoL will be measured at ten points up to five years after study entry:

The first point of assessment is after informed consent has been obtained, but before randomization. Information to the patient about to which treatment arm she has been randomized might have an impact on her HRQoL. Thus, it is very important that the patient is unaware of the result of randomization when responding to the QoL-questionnaires. This point of assessment provides HRQoL estimates unaffected by side effects of treatment. It should be kept in mind, however, that the included patients have recently being informed about their breast cancer diagnosis. Thus, their HRQoL cannot be regarded as a true "baseline" assessment. This point of assessment will give a measure of HRQoL that allows for investigation of differences in HRQoL between the treatment arms at inclusion. If there are differences in HRQoL at the first point of assessment, these can be adjusted for when analyzing the variations in HRQoL during the course of treatment. In addition, biased results due to selective attrition during the study period may also be analyzed by comparing those who participate with those who are lost at subsequent points of assessment.

The following ten points of assessment are: after 6 and 12 weeks before evaluation of the endocrine treatment and randomization, and after 18 and 24 weeks before surgery, three months after surgery (to compare the various treatments), one year after surgery and thereafter annually up to five years (to evaluate the treatments at points defined for clinical follow-up).

9.2.3 Instruments

European Organization for Research and Treatment of Cancer Quality of Life Questionnaire-C30, version 3.0 (EORTC QLQ-C30) is a HRQOL instrument developed to be multidimensional in structure and self-administrative to be used in cancer clinical trials (Aaronson et al 1993). It includes nine multi-item scales and six single item variables. The five functional scales consist of physical- (PF), role- (RF), emotional- (EF), social- (SE), and cognitive functioning (CF). Fatigue (FA), nausea/vomiting (NV) and pain (PA) comprise the three multi-item symptom scales. Additional symptoms are assessed by single items: dyspnoea (DY), sleep disturbances (SL), appetite loss (AP), constipation (CO), and diahorrea (DI). One single item scale concern financial problems related to disease and treatment. Most items are responded to on a four-point scale ranging from 1 (not at all) to 4 (very much). The two items assessing global health and overall quality of life are responded to in seven categories ranging from 1 (very poor) to 7 (excellent).

The EORTC QLQ Breast Cancer Module (QLQBR-23) is a breast cancer specific questionnaire developed for use among patients varying in disease stage and treatment modality (Sprangers et al 1996). It comprises 23 items divided into four functioning scales: body image (BRBI), sexual functioning (BRSEF), sexual satisfaction/enjoyment (BROSE), and future perspective (BRFU); and four symptom scales: systemic therapy side effects (BRST), breast symptoms (BRBS), arm symptoms (BRAS), and being upset by hair loss (BRHL). The questionnaire has been validated in aninternational study (Sprangers et al 1996). Completion of the questionnaire takes about 10–20 min. The items are responded to in the same four categories as most items in the EORTC QLQ-C30.

9.2.4 Procedure

The physician including the patient in the study introduces the HRQOL-evaluation and the patient receives the baseline questionnaire together with an information sheet and an envelope. After completion, the questionnaire in the envelope is collected by the physician who hands it over to the research nurse. The reason for using an envelope at this assessment point is to prevent that the treating physician has access to the questionnaire. This enables the patient to respond more freely, minimizing bias due to social desirability.

At subsequent HRQOL assessments, the patient is given the questionnaire by the research nurse. The questionnaire should be completed before any examination or treatment in order to avoid the responses to be influenced by medical information given. The research nurse collects the questionnaire. No staff responsible for the treatment of the patient will have access to the questionnaires and it is important that the patient is informed about this procedure. The data in the questionnaires are entered in the clinical database by the research nurse. Analysis of questionnaire data will be performed according to the recommendations by the original authors. Differences between the randomisation arms and over time will be evaluated.

9.2.5 Data analysis

In this study HRQoL is a secondary endpoint. Data for the EORTC QLQ-C30/BR23 will be scored according to the algorithm described in the EORTC QLQ-C30 scoring manual (Fayers et al 2001). All scales and single items are scored on categorical scales and linearly transformed to 0-100 scales where a high score for

1. a symptom scale or item represents a high level of symptoms or problems
2. a functional scale represents a high or healthy level of functioning
3. the global health status/QoL represents high QoL.

Compliance with completing QoL questionnaires will be investigated at each time point to evaluate the procedure for data collection and the feasibility of the questionnaires.

The effect of treatment on each of the scale scores at the different time points will be evaluated using linear regression models including both treatment and baseline values for the studied scales. Considering multiple testing, the results from the regression analysis will be presented as mean differences together with 99 % confidence intervals. Linear mixed-models will be used to study treatment, time, and the treatment-time interaction using all available longitudinal data.

In the interpretation of the QLQ-C30 and BR-23 scores, a difference of C5 points on the 0–100 scales will be considered clinically important. Differences of 5–9 points are considered small, those of 10–20 as moderate, and those ≥20 as large (Osoba et al 1998).

Due to multiple testing, the level of statistical significance will be set to 0.01 to avoid type I errors. All analyses will be performed according to the ‘‘intention-to-treat’’ principle.

9.3 References

Aaronson NK, Ahmezai S, Bergman B, et al. The European Organization for Research and Treatment of Cancer QLQ-C30: A quality of life instrument for use in international clinical trials in oncology. J Natl Cancer Inst 1993;85:365-376

Fayers PM, Aaronson NK, Bjordal K, Groenvold M, Curran D, Bottomley A on behalf of the EORTC Quality of Life Group. The EORTC QLQ-C30 Scoring Manual (3rd edititon). European Organization for Research and Treatment of Cancer. Brussels 2001

Osoba D, Rodrigues G, Myles J et al. Interpreting the significance of changes in health-related

quality-of-life scores. J Clin Oncol 1998;16:139–144

Sprangers MAG, Groenvold M, Arraras JI, et al. The European Organization for Research and Treatment of Cancer breast cancer-specific quality-of-life questionnaire module: first results from a three-country field study. J Clin Oncol 1996;14:2756-2768

10 NEXT-GENERATION SEQUENCING

Henrik Grönberg, Daniel Klevebring

With the advances in next-generation sequencing, much effort has been focused on translational oncology. In ClinSeq, we have established a pipeline for genomic profiling of cancer using fresh-frozen tissue (2x2x2 mm or more) enabling subclassification using both intrinsic subtypes (Sørlie et al 2003) and standard markers (ER, PR, HER2, Ki-67). Our analysis is based on RNAseq, low-pass whole genome sequencing and deep exome sequencing of 550 selected genes.

10.1 Background

There are several established molecular biomarkers and molecular subtypes that are relevant for patients diagnosed with breast cancer. These include both prognostic markers and those predictive of potential response to early breast cancer treatment.

Protein biomarkers: In current standard pathology, immunohistochemistry (IHC) is used to determine expression of four protein markers. Hormone receptors, i.e. estrogen receptor (ER) and progesterone receptor (PR) provide prognostic information (Colzani et al 2011). Expression of ER is also a well-established predictor of response to tamoxifen (EBCTCG 2011). Further, expression levels of Ki67, associated with cell proliferation, have demonstrated prognostic value in meta-analysis (de Azambuja et al 2007).

Transcriptomic biomarker panels: The so-called intrinsic molecular subtypes of breast cancer (Basal-like, Luminal A, Luminal B, HER2 and Normal-like) are based on genome-wide gene expression profiling (Sørlie et al 2003). These subtypes have demonstrated prognostic value, and the expression of these gene clusters are directly related to therapeutic targets (eg. ER and HER2) and predictive of response to treatment. There are multiple proposed biomarker panels based subsets of gene expression, including PAM50 (Parker et al 2009), the Amsterdam 70-gene panel (Mammaprint; van't Veer et al 2002) and Oncotype DX (Paik et al 2004). The biomarker panel with the strongest evidence of clinical utility is the PAM50 panel, which predicts samples allocated to the intrinsic sub-types. The PAM50 panel is also utilized to calculate the Risk of Recurrence (ROR) Score, which has been demonstrated to be prognostic and predictive for node negative breast cancer (Parker et al 2009). PAM50 and the ROR score have demonstrated prognostic performance beyond conventional clinical factors (Dowsett et al 2013), suggesting added value of molecular profiling. Gene expression based subtyping of BC tumors, particularly using sequencing, is an active area of research and development, with the potential to further improve clinical utility. The ClinSeq project include both whole transcriptome profiling through RNA sequencing, relevant for subtyping of breast tumors, and panel sequencing and profiling of copy number variation.

10.2 Methods

Tissue processing: We have developed a protocol enabling the harvesting of fresh tissue in the conventional clinical setting. Fresh-frozen tissue is embedded in OCT after which 200µm is sectioned, with adjacent 5µm H&E sections for histopathological review of tumor content.

Pre-processing for genomic profiling: The pre-processing is optimized

1. to allow for minute amounts of DNA/RNA
2. to facilitate the use of formalin-fixed paraffin embedded material when the tissue collection procedure fails and
3. to be cost-effective.

For each patient, three libraries are constructed for sequencing, which enables us to interrogate copy-number perturbations, point mutations in a panel of 550 genes and information in the transcriptome. For the RNA library, sequencing is carried out generated through on average 30 million reads per sample to be used for subtyping as well as identification of the expression of ESR1, PGR and MKI67 (ER, PR and Ki-67, respectively). The panel is sequenced to an average coverage of 200x to gain high power to detect somatic mutations as low frequencies, for example activating mutations in ERBB2 (HER2). For copy number analysis, low-pass whole genome sequencing is carried out to approximately 0.5 - 1x coverage. This enables the detection of somatic amplification of ERBB2 (HER2) as well as other somatic copy number alterations, for example amplification of the MYC oncogene or deletions of the TP53 tumor suppressor.

Automated analysis pipeline: In order to process the generated data, we have developed an automated analysis pipeline based on state-of-the-art algorithms for each data type. During the development of the pipeline, a multitude of algorithms were evaluated based on performance and required computational resources. For each patient, a genomic profile focused on the genomic alterations in the patient's tumor is generated. Importantly, as the profiles are intended for clinical implementation, the RNAseq data is used to validate the presence of point mutations and structural perturbations (gene-fusions and copy number events) through outlier analysis.

10.3 References

Colzani E, Liljegren A, Johansson AL, et al. Prognosis of patients with breast cancer: causes of death and effects of time since diagnosis, age, and tumor characteristics. J Clin Oncol 2011;29(30):4014-21

de Azambuja E, Cardoso F, de Castro G Jr, et al. Ki-67 as prognostic marker in early breast cancer: a meta-analysis of published studies involving 12,155 patients. Br J Cancer 2007;96(10):1504-13

Dowsett M, Sestak I, Lopez-Knowles E, et al. Comparison of PAM50 risk of recurrence score with oncotype DX and IHC4 for predicting risk of distant recurrence after endocrine therapy. J Clin Oncol 2013;31(22):2783-90

Early Breast Cancer Trialists' Collaborative Group (EBCTCG), Relevance of breast cancer hormone receptors and other factors to the efficacy of adjuvant tamoxifen: patient-level meta-analysis of randomised trials. Lancet 2011;378(9793):771-84

Paik S, Shak S, Tang G, et al. A multigene assay to predict recurrence of tamoxifen-treated, node-negative breast cancer. N Engl J Med 2004;351(27):2817-26

Parker JS, Mullins M, Cheang MC, et al. Supervised risk predictor of breast cancer based on intrinsic subtypes. J Clin Oncol 2009;27(8):1160-7

Sørlie T, Tibshirani R, Parker J, et al. Repeated observation of breast tumor subtypes in independent gene expression data sets. Proc Natl Acad Sci U S A 2003;100(14):8418-23

van 't Veer LJ, Dai H, van de Vijver MJ, et al. Gene expression profiling predicts clinical outcome of breast cancer. Nature 2002;415(6871):530-6

11 PHARMACOGENETICS

Hanjing Xie

11.1 Background

Pharmacogenetics may have a major impact on clinical response. Dosing of drugs related to body weight or body surface area can cause significant differences in terms of efficacy and undesired side effects between patients. The intrinsic inter-individual variations are caused by inheritance of genetic variations encoding drug metabolizing enzymes or drug transporters. The impact of pharmacogenetics on the effects of anticancer drugs may have impact on the relapse rate, and, as a consequence, the overall survival.

The emergence of pharmacogenetics has thrown light on the issue of optimizing antitumoral therapy aiming at individually dosed treatment with an acceptable balance between side effects and efficacy.

In the present trial, we intend to study the pharmacogenetic prerequisites for the involved cytotoxic drugs:

Tamoxifen and CYP2D6/CYP2C19: Tamoxifen is a “prodrug” and it requires metabolic activation to elicit the desired pharmacological activity. Cytochrome P450 2D6 (CYP2D6) plays an important role in the catalyzation of tamoxifen to the effective metabolite endoxifen. Several studies have shown the relationship between the CYP2D6 phenotyping and the different clinical outcomes of tamoxifen (Goetz MP et al 2005; Schroth W et al 2009). Patients, who are poor or intermediate metabolizers of tamoxifen, correlate with significantly shorter time to recurrence and worse RFS, compared to those who are extensive or ultrarapid metabolizers. The role of CYP2D6 as a genetic predictor for the treatment response of tamoxifen has been discussed.We showed in our previously published study (Margolin S et al 2013) that in patients who were treated with tamoxifen for at least one year there was an association between reduced CYP2D6 activity (≤50 % of normal) and recurrence ($p = 0.025$) and breast cancer-specific mortality ($p = 0.034$). In a multivariable analysis, CYP2D6 remained an independent predictor of outcome. In a subgroup analysis, the effect of CYP2D6 seemed to derive mainly from premenopausal patients, which represents a new finding that needs validation in a larger study sample. Recently, we performed a further study to investigate the effect of different CYP2C19 genotype categories corresponding to different metabolic phenotypes on breast cancer recurrence and mortality in the same study material. A combined genotype analysis was envisioned using both CYP2C19 and CYP2D6. Our data showed for the first time in the literature that *CYP2C19*17* together with expected *CYP2D6* activity can clearly serve as predictive biomarkers for both breast cancer recurrence and breast cancer specific survival in premenopausal patients ($P=0.008$, data not published). In this study, we aim to investigate the effect of CYP2D6 as well as CYP2C19 genotype on tamoxifen treatment.

Letrozole and CYP2A6: Large inter-patient variability has been found in letrozole pharmacokinetics. Plasma letrozole concentrations vary more than 10-fold among breast cancer patients with quantifiable levels of letrozole (Desta Z et al 2011; Hiromi T et al 2011; Murai K et al 2009)). Letrozole is mainly metabolized in the liver by the CYP 2A6 enzyme (Jeong S et al 2009; Goh BC et al 2002), the gene of which is highly polymorphic. Up to date almost 40 allelic variants have been identified and several alleles lead to decreased or abolished enzyme activity (see

www.cypalleles.ki.se). Furthermore, *CYP2A6* gene deletion and duplication events occur. Recently, it was shown that genetic polymorphism of *CYP2A6* influences the plasma concentration of letrozole (4). In this study, we aim to investigate the effect of *CYP2A6* genotype on letrozole treatment.

Palbociclib: Pharmacogenetic effects of the target enzymes that metabolize palbociclib and may predict toxicity or response to treatment have so far not been studied. Genes relevant for both the pharmacodynamics and the pharmacokinetics of palbociclib will be analyzed.

11.2 Methods

5 ml whole blood will be collected from the patients before treatment. DNA will be prepared from the blood samples. Various PCR methods will be used. The applied PCR analyses include restriction fragment length polymorphism (RFLP), sequence analysis and TaqMan PCR. The genetic variations will be recorded in a database and the genotype will be correlated to the phenotypes. Genotype frequencies in the population will be recorded.

11.3 References

Desta Z, Kreutz Y, Nguyen AT, et al. Plasma letrozole concentrations in postmenopausal women with breast cancer are associated with CYP2A6 genetic variants, body mass index, and age.Clin Pharmacol Ther 2011;90(5):693-700

Goetz MP, Rae JM, Suman VJ, et al. Pharmacogenetics of tamoxifen biotransformation is associated with clinical outcomes of efficacy and hot flashes. J Clin Oncol 2005;23(36):9312-8

Goh BC, Lee SC, Wang LZ, et al. Explaining interindividual variability of docetaxel pharmacokinetics and pharmacodynamics in Asians through phenotyping and genotyping strategies. J Clin Oncol 2002;20(17):3683-90

Tanii H, Shitara Y, Horie T. Population pharmacokinetic analysis of letrozole in Japanese postmenopausal women. Eur J Clin Pharmacol 2011;67(10):1017-25

Jeong S, Woo MM, Flockhart DA, Desta Z. Inhibition of drug metabolizing cytochrome P450s by the aromatase inhibitor drug letrozole and its major oxidative metabolite 4,4'-methanol-bisbenzonitrile in vitro. Cancer Chemother Pharmacol 2009;64(5):867-75

Margolin S, Lindh JD, Thorén L. CYP2D6 and adjuvant tamoxifen: possible differences of outcome in pre- and post-menopausal patients. Pharmacogenomics 2013;14(6):613-22

Murai K, Yamazaki H, Nakagawa K, et al. Deactivation of anti-cancer drug letrozole to a carbinol metabolite by polymorphic cytochrome P450 2A6 in human liver microsomes. Xenobiotica. 2009;39(11):795-802

Schroth W, Goetz MP, Hamann U, et al. Association between CYP2D6 polymorphisms and outcomes among women with early stage breast cancer treated with tamoxifen. JAMA. 2009;302(13):1429-36

12 INTRA-TUMOR HETEROGENEITY BY SINGLE-CELL SEQUENCING

Linda Lindström

12.1 Background

Breast cancer tumors are among the most heterogeneous human cancers. Gene expression profiling by microarray has provided insight into the inter-tumor heterogeneity among breast cancer tumors and have shown that breast cancer tumors may be subdivided into different subtypes from gene expression profiling patterns that closely relate to the clinically used breast cancer markers each with different prognostic characteristics serving for therapeutic decisions in the adjuvant setting (Parker et al 2009; Sørlie et al 2001, van't Veer et al 2002, Weigelt et al 2012).

We and others have recently shown that the clinically used breast cancer markers alter their expression throughout tumor progression (Amir et al 2012, Lindström et al 2012, Niikura et al 2012). Interestingly, this intra-patient heterogeneity between primary tumor and metastasis influences patient survival (Lindström et al 2012, Thompson et al 2010). What are the likely explanations to our findings? It has been suggested that primary breast tumors may possess *intra-tumor heterogeneity* with varying metastatic capacity and response to therapy (Fidler & Kripke 1977, Poste & Fidler 1980).

In 1976, a landmark paper was published introducing the evolutionary biology concept of 'survival of the fittest' to the field of tumor progression (Nowell 1976). It was proposed that tumors with greater intra-tumor heterogeneity adapt faster compared to homogeneous tumors, because of the fact that the beneficial characteristics are more likely to already be present in the tumor, rather than needing to occur *de novo*. This may be essential in defining primary tumor aggressiveness and metastatic capacity since tumors with greater intra-tumor heterogeneity have the potential to shed tumor cells to the circulatory systems with a spectra of different characteristics of which a portion may potentially already accommodate beneficial characteristics to enable survival and later invasion at a distant site.

Today, next-generation sequencing approaches have improved in precision and throughput, but also decreased greatly in cost, which has enabled the sequencing of entire patient genomes. Typically these techniques are based on bulk analyses, which means that they estimate the mean sequence based on signals from millions of cells. However, one new technique called *single-cell next-generation sequencing* enables the sequencing of one cell thus giving information of the DNA or RNA sequence for each cell separately.

12.2 Methods

After enzymatic disaggregation, 96 representative tumor cells (two 48-well plates) will be selected from each patient biopsy (by manual picking under the microscope or by Fluorescence Assisted Cell Sorting [FACS]) and the selected single cells will be placed into individual reaction chambers for lysis and further processing. Following the selection of the single tumor cells from the patient biopsies, we will proceed and sequence each tumor cell by transcriptomic whole-genome new generation sequencing. The single-cell sequencing molecular analysis protocol as proposed by Linnarsson et al is currently running in the Kere Laboratory and will be utilized (Islam et al 2011).

In brief, the single cells will be individually reverse-transcribed with poly(T) primers and 5' individually tagged RNA primers to allow for strand-switch extension of the cDNA. The single cells will then be pooled for PCR amplification and further processing to make 5' expression tag libraries, which will be sequenced on an Illumina 2500 instrument to a depth of approximately 10 million tags

per sample.

Intra-tumor heterogeneity will be determined by contrasting genes expressed in the 96 single cells from each biopsy in relation to tumor aggressiveness and response to therapy using statistical methods as needed for single-cell transcriptomic statistical analyses such as SAMstrt (Katayama et al 2013). In addition, intra-tumor heterogeneity before, during and after neoadjuvant therapy will be evaluated.

12.3 References

Amir E, Miller N, Geddie W. Prospective study evaluating the impact of tissue confirmation of metastatic disease in patients with breast cancer. J Clin Oncol 2012;30(6):587–92

Fidler I, Kripke M. Metastasis results from preexisting variant cells within a malignant tumor. Science (80-) 1977;197(4306):893–5

Islam S, Kja U, Moliner A, et al. Characterization of the single-cell transcriptional landscape by highly multiplex RNA-seq. Genome Res 2011;21:1160–7

Katayama S, Töhönen V, Linnarsson S, Kere J. SAMstrt: statistical test for differential expression in single-cell transcriptome with spike-in normalization. Bioinformatics 2013;29(22):2943–5

Lindström LS, Karlsson E, Wilking UM, et al. Clinically Used Breast Cancer Markers Such as Estrogen Receptor, Progesterone Receptor, and Human Epidermal Growth Factor Receptor 2 Are Unstable Throughout Tumor Progression. J Clin Oncol 2012;30(21):2601–8

Niikura N, Liu J, Hayashi N, et al. Loss of Human Epidermal Growth Factor Receptor 2 (HER2) Expression in Metastatic Sites of HER2-Overexpressing Primary Breast Tumors. J Clin Oncol 2012;30(6):1–8

Nowell PC. The clonal evolution of tumor cell populations. Science (80-) 1976;194(4260):23–8

Parker JS, Mullins M, Cheang MCU, et al. Supervised risk predictor of breast cancer based on intrinsic subtypes. J Clin Oncol 2009;27(8):1160–7

Poste G, Fidler I. The pathogenesis of cancer metastasis. Nature 1980;283:139–46

Sørlie T, Perou CM, Tibshirani R, et al. Gene expression patterns of breast carcinomas distinguish tumor subclasses with clinical implications. Proc Natl Acad Sci U S A 2001;98(19):10869–74

Thompson AM, Jordan LB, Quinlan P, et al. Prospective comparison of switches in biomarker status between primary and recurrent breast cancer: the Breast Recurrence In Tissues Study (BRITS). Breast Cancer Res 2010;12(6):R92

van't Veer LJ, Dai H, van de Vijver MJ, et al. Gene expression profiling predicts clinical outcome of breast cancer. Nature 2002;415(345):530–6

Weigelt B, Pusztai L, Ashworth A, Reis-Filho JS. Challenges translating breast cancer gene signatures into the clinic. Nat Rev Clin Oncol 2012;9(1):58–64

13 SMALL RNAS IN TUMOR AND PLASMA/SERUM

Barbro K Linderholm

13.1 Background

MicroRNAs (miRNAs) are endogenous small noncoding RNAs (18-21 nucleotides long) that negatively regulate the gene expression at the post-transcriptional level inducing mRNA degradation and/or translational repression by imperfect pairing with 3' untranslated region (UTR) sequences of their target mRNAs. They are involved in the regulation of many biological processes such as proliferation, differentiation, apoptosis, stress response and tumorigenesis (Caldas and Brenton 2005; Calin and Croce 2006; Krek et al 2005). In addition, a number of recent studies reported that miRNA aberrant expression is implicated in anticancer drug resistance in breast cancer (Tavazoie et al 2008; Foekens et al 2008) and other tumor types.

miRNAs have been considered to have potential to become useful cancer biomarkers for several reasons: miRNAs are deregulated in cancer, their expression is tissue specific and they are relatively stable in paraffin-embedded and frozen tissues. A number of recent studies have shown the presence and stability of miRNAs in the blood circulation which enables use of circulating miRNAs as minimally invasive biomarkers (Mitchell et al 2008).

Vault RNAs (vRNAs) represent another, less known, class of small noncoding RNAs which are part of the vault ribonucleoprotein (RNP) complex (Stadler et al 2009). Several studies have suggested an implication of vault RNP in drug resistance of cancer (Hussain et al 2013; Gopinath et al 2010). Moreover, recent findings have shown that vRNAs can be processed by DICER in small vault RNAs (svRNAs) and can act in a miRNA-like manner. Interestingly, one of these svRNAs downregulates the expression of CYP3A4.

The *overall aim* is to develop predictive tests based on profiling of small RNAs.

13.2 Methods

miRNAs and vRNAs will be isolated from fresh tumor and serum at base-line, 12, 18 (serum only) and 24 weeks of treatment. Total RNA will be isolated using a similar approach previously described by Mitchell PS and collaborators (Mitchell et al 2008) with some modifications.

Taqman Array Human MicroRNA Cards (Applied Biosystem), containing 754 unique assays specific to known mature human miRNAs, will be adopted to analyze the global miRNA expression profiles of 80 selected patients at initial diagnosis (*discovery cohort*). Forty nanograms of RNA for each sample are used as input: the RNA is reverse-transcribed using Megaplex RT primer-pool and the generated cDNA is pre-amplified using Megaplex PreAmp primers. Validation of selected miRNAs will be performed by quantitative Real Time-PCR (RT-qPCR) in the remaining cohort of included patients (*validation cohort*).

Evaluation of the expression levels of svRNAa and svRNAb will be performed using TaqMan RT-qPCR assays. A fix amount (1microliter, diluted 1:3) of RNA from each sample is used for generation of cDNA using specific primers for svRNAa, svRNAb, and the three *C. Elegans* miRNAs *cel-miR-39*, *cel-miR-54* and *cel-miR-238* which were spiked-in during RNA isolation as previously described. Subsequently, the expression levels of the two svRNAs and the three *C,Elegans* miRNAs were measured using TaqMan RT-qPCR assays.

Given the early stage of serum miRNA research, there are so far no established endogenous small RNA controls for normalization purposes. Thus, for data normalization, we will use an approach

based on spiking in the three synthetic *C. Elegans* miRNAs which are chosen due to the absence of homologous sequences in human genome.

The goal is to develop a robust, cost-efficient predictive test for studied drugs. A secondary aim is to compare potential predictive profiles from tumor and circulation.

13.3 References

Caldas C, Brenton JD. Sizing up miRNAs as cancer genes. Nat Med 2005;11(7):712-4

Calin GA, Croce CM. MicroRNAs and chromosomal abnormalities in cancer cells. Oncogene. 2006;25(46):6202-10

Krek A, Grün D, Poy MN et al. Combinatorial microRNA target predictions. Nat Genet 2005;37(5):495-500

Tavazoie SF, Alarcón C, Oskarsson T et al. Endogenous human microRNAs that suppress breast cancer metastasis. Nature 2008;451(7175):147-52

Foekens JA, Sieuwerts AM, Smid M et al. Four miRNAs associated with aggressiveness of lymph node-negative, estrogen receptor-positive human breast cancer. Proc Natl Acad Sci U S A 2008;105(35):13021-6

Mitchell PS, Parkin RK, Kroh EM, et al. Circulating microRNAs as stable blood-based markers for cancer detection. Proc Natl Acad Sci U S A 2008;105(30):10513-8

Stadler PF, Chen JJ, Hackermüller J, et al. Evolution of vault RNAs. Mol Biol Evol 2009;26(9):1975-91

Hussain S, Sajini AA, Blanco S, et al. NSun2-mediated cytosine-5 methylation of vault noncoding RNA determines its processing into regulatory small RNAs. Cell Rep 2013;4(2):255-61

Gopinath SC, Wadhwa R, Kumar PK. Expression of noncoding vault RNA in human malignant cells and its *importance* in *mitoxantrone* resistance. Mol Cancer Res 2010;8(11):1536-46

14 TUMOR AND PLASMA PROTEOMICS

Janne Lehtiö

14.1 Background

Changes in the proteome level provide readouts from a combined picture of genotype and environmental effects. A direct measurement of single target gene/protein as a clinical therapy predictor has shown to be difficult due to the complex regulatory networks, tumor microenvironment and genomic heterogeneity (Chong and Janne 2013). Further, the large number of mutations and genomic aberrations makes it difficult to differentiate between driver and passenger mutations. Our hypotheses is that the effects of the myriad of genomic aberrations in patient tumors canalize into effects on a limited number of signaling pathways, which are clearly manifest at the functional proteomics level. Proteomics level measurements have other benefits, namely both plasma based protein markers and immunohistological markers are widely used in clinics today, hence the path from discovery to clinical utility can be shortened.

Proteomics is a collective term for techniques used for analysis of a proteome. In recent years, the development of analytical platforms and methods in MS based proteomics has resulted in tremendously increased proteome coverage and improved quantification in each experiment. We have recently published a novel in-depth proteomics method based on two step fractionation of the sample (HiRIEF LC-MS/MS), which improves the sensitivity of quantitative proteomics a lot (Branca et al 2014). This method allows comprehensive quantitative proteomics analysis on clinical samples reaching the comparable level of analytical depth as transcriptomics by deep sequencing (Johansson et al 2013; Branca et al 2014). These advances in discovery proteomics have opened a new window to profile the molecular phenotype of tumors and couple this information to genotype and clinical phenotype.

In a recent study, we applied this in-depth proteomics method to study endocrine resistance in breast cancer (Johansson et al 2013). We show that the nuclear receptor retinoic acid receptor alpha (RARA) was upregulated in endocrine resistant cell lines and breast tumor samples. Following bioinformatic analysis of proteomics data, the concluding hypothesis was that the ER and RARA protein interaction environment dictate the endocrine response in sub-cohort of patients. Further, we have developed novel methods for clinical material analysis and applied these in several tumor types. These studies demonstrate the feasibility of proteomics for cancer biomarker discovery.

We have studied proteomics in EFGR and RET tyrosine kinase driven tumors both on cell line and tumor lysate levels. Our preliminary findings indicate that early adaptation leading to resistance can be detected by monitoring protein level changes and phospho-proteomic patterns. In the EGFR study we can detect protein changes related to tyrosine kinase switch, anti-apoptotic signaling and EMT switch.

In summary, tumor proteomics can be used to understand molecular response to treatment and the detected protein level and post-translational modification changes can be converted to biomarker candidates. This analysis can provide also systems biological information to combine with mutation analysis and transcriptomics.

14.2 Methods

Core biopsy for proteomics analysis in each indicated instance and small fresh frozen tissue with operation (adjacent normal tissue and tumor when possible). Plasma samples, 200µl (0.2 ml), in each indicated plasma collection instance.

14.3 References

Branca RM, Orre LM, Johansson HJ, et al. HiRIEF LC-MS enables deep proteome coverage and unbiased proteogenomics. Nat Methods 2014;11:59-62

Chien AJ, Moasser MM. Cellular mechanisms of resistance to anthracyclines and taxanes in cancer: intrinsic and acquired. Semin Oncol 2008;35:S1-S14, quiz S39.

Chong CR, Janne PA. The quest to overcome resistance to EGFR-targeted therapies in cancer. Nat Med 2013;19:1389-1400

Johansson HJ, Sanchez BC, Mundt F, et al. Retinoic acid receptor alpha is associated with tamoxifen resistance in breast cancer. Nat Commun 2013;4:2175

15 STEM CELL ANALYSES

Johan Hartman

15.1 Background

Breast cancer stem cells are associated with tumor initiation and metastasis (Ginestier et al 2007; Pece et al 2010). Our research shows that these cells are not a genetically defined subpopulation but should be considered a phenotypic state (Klevebring et al, manuscr. 2014). Breast cancer is a complex disease of substantial intratumoral heterogeneity on the genetic level. However, breast cancer stem cells confer another level of phenotypic complexity onto the genetic complexity, probably as a consequence of epigenetic regulation. Breast cancer stem cells can be isolated by a combination of certain well-defined markers; CD44+/CD24-, ALDH1^{High} as well as the embryonic stem cell genes OCT3, NANOG, and SOX2.

15.2 Methods

The cancer stem cell content in preoperative breast cancer biopsies and in surgically resected breast cancer specimens are analyzed by immunohistochemistry. CD44, CD24, ALDH1, OCT3, NANOG and SOX2 will be analyzed. Additionally, a collaborative project to identify novel stem cell markers will be performed with the intention to evaluate the predictive role of potential stem cell markers and the impact of treatment on cancer stem cell suppression.

15.3 References

Ginestier C, Hur MH, Charafe-Jauffret E, et al. ALDH1 is a marker of normal and malignant human mammary stem cells and a predictor of poor clinical outcome. Cell Stem Cell. 2007;1:555–67

Klevebring D, Rosin G, Ma R, et al. Sequencing of tumorigenic cancer cell populations indicates a dynamic conversion between differentiation states in breast cancer in vivo (submitted manuscript)

Pece S, Tosoni D, Confalonieri S, et al. Biological and molecular heterogeneity of breast cancers correlates with their cancer stem cell content. Cell. 2010;140:62–73

16 CHARACTERIZATION OF TUMOR STROMA

Arne Östman

16.1 Background

Properties of the tumor stroma affect the growth and response characteristics of solid tumors (Pietras and Östman 2010; Östman 2012; Östman 2014). Furthermore, stromal responses, such as changes in the tumor vasculature, might also act as early indicators of response (Östman and Heldin 2007). In the present study, immunohistochemistry (IHC)-based characterization of tumor stroma will be performed both on tissue collected from the pretreatment biopsy and biopsies taken during and after termination of the study treatment.

16.2 Methods

Primarily, the analyses will use procedures for stroma characterization previously published by the Östman group (Paulsson et al 2009; Hägglöf et al 2010; Frings et al 2013).

The second set of analyses will exploit a novel approach for multi-parametric stroma characterization (Mezheyeuski, ASCO 2014) which uses 4-6 IHC double-stainings with endothelial cell markers (CD31 or CD34) together with a panel of fibroblast and pericyte markers (PDGFbR, PDGFaR, alpha-smooth-muscle-actin and desmin). Subsequent staining sections are subjected to a set of house-developed image analysis algorithms which will yield information about properties comprising vessel density, average vessel size, pericyte coverage of marker-defined subsets and characterization of fibroblast stroma abundance and marker status.

The results from the analyses will then be correlated to clinical endpoints, with special reference to the prediction of response to treatment. Comparison of the results from the two biopsies taken before and after two cycles of treatment, respectively, will be used in order to detect whether changes in the tumor stroma can be used as early markers of clinical response.

IHC will be performed on standard PFA fixed and paraffin-embedded tissue sections derived from pretreatment biopsies and from the biopsies taken during treatment. It is estimated that 10-15 sections from each biopsy will suffice for these analyses.

Analyses and evaluation regarding the predictive value will be performed based on tissue microarrays on samples from the pretreatment biopsies as well as from tissue obtained during and after termination of the treatment.

16.3 References

Frings O, Augsten M, Tobin NP, et al. Prognostic significance in breast cancer of a gene signature capturing stromal PDGF signaling. Am J Pathol 2013;182(6):2037-47

Hägglöf C, Hammarsten P, Josefsson A, et al. Stromal PDGFRbeta expression in prostate tumors and non-malignant prostate tissue predicts prostate cancer survival. PLoS One 2010;5(5):e10747

Paulsson J, Sjöblom T, Micke P, et al. Prognostic significance of stromal platelet-derived growth factor beta-receptor expression in human breast cancer. Am J Pathol 2009;175(1):334-41

Östman A, Heldin CH. PDGF receptors as targets in tumor treatment. Adv Cancer Res 2007;97:247-74

Östman A. The tumor microenvironment controls drug sensitivity. Nat Med 2012;18(9):1332-4

Östman A. Cancer-associated fibroblasts: Recent developments and emerging challenges. Semin Cancer Biol 2014 Feb 18

Pietras K, Östman A. Hallmarks of cancer: interactions with the tumor stroma. Exp Cell Res 2010;316(8):1324-31

17 AMOTL2 EXPRESSION IN EARLY BREAST CANCER

Lars Holmgren

17.1 Background

The majority of human cancers arise in tissues of epithelial origin and account for more than 80 % of all cancer-related deaths (http://www.cancer.org/). A strong correlation between malignancy and loss of epithelial organization has been documented for most carcinomas which may be attributed to loss of polarity (Bilder 2004). The deregulation of apical-basal polarity is often depicted as a hallmark of cancer although direct evidence whether this is a cause or consequence of tumor progression is not yet clear (Lee et al 2008; Royer et al 2011). Our group has identified the Amot protein family involved in tumor angiogenesis and growth (Aase et al 2007; Ernkvist et al 2009; Yi et al 2011). One of its members, AmotL2, is involved in a novel molecular pathway that negatively controls apical-basal polarity in tumor cells (Mojallal et al, final rev. Nature Comm.). We have shown that hypoxia upregulates p60 AmotL2 which then sequesters apical polarity proteins in intracellular vacuoles. The consequence is loss of polarity which potentiates the response to invasive cues. Our studies show also that p60 AmotL2 expression correlates with loss of polarity in human breast and colorectal cancer material. The present research proposal builds on these findings in order to further investigater the prognostic, descriptive and functional relevance of this signaling pathway.

We have analyzed to date over 135 colon cancer and 120 breast cancer specimen. The results show that AmotL2 expression correlates with loss of polarity, invasion and poor survival (Mojallal, Nature Comm., in revision). In addition, we have submitted our antibodies to the human protein atlas consortium (HPA.org) which indicates a similar distribution also in other cancers such as glioblastoma, prostate and pancreas cancer. Our data further show that the expression pattern overlaps with markers for hypoxia such as carbo-anhydrase 9 and Glut1. Taken together, p60 AmotL2 expression in human cancer correlates with hypoxia, loss of polarity and invasion. We also used short hair pin RNA to target AmotL2 in breast cancer cells. With this approach, we could restore polarity resulting in loss of tumorigenicity when orthotopically transplanted in mammary fat pads in mice. *In vitro*, we have elucidated the mechanisms by which AmotL2 deregulates polarity. AmotL2 binds polarity proteins (Crb3 and Par3) and sequesters them in intracellular vacuoles, which is a novel finding. We have addressed the issue whether AmotL2 is upregulated in normal but ischemic tissues by investigating mouse hearts exposed to 30 minutes ischemia followed by 30 minutes reperfusion and patients suffering from myocardial infarction. The results show that AmotL2 is a rapidly induced, robust marker for ischemic tissue.

17.2 Methods

AmotL2 antibodies for paraffin- or cryo-sections have been generated and evaluated by Lars Holmgrens group and by protein atlas. Sections will be subjected to antigen-retrieval and stained according to standard immunohistochemical protocols. All samples will be read blindly. Staining will be arbitrarily stratified between 0-3. The highest stained area will be used for classification. AmotL2 expression will be correlated to response to therapy and to disease-free and breast cancer related survival. Statistical analysis includes Gehan-Wilcoxon univariate tests.

Antibodies have been validated on frozen and paraffin sections from 122 breast cancer patients. These data are under revision in Nature Comm.

Cancer therapy suffers from the lack of reliable and robust surrogate markers. It is feasible that treatment is partly dependent on the response of tumor cells to cellular stress signals. This makes AmotL2 an interesting candidate to evaluate.

17.3 References

Aase K, Ernkvist M, Ebarasi L, et al. Angiomotin regulates endothelial cell migration during embryonic angiogenesis. Genes Dev 2007;21:2055-2068

Bilder D. Epithelial polarity and proliferation control: links from the Drosophila neoplastic tumor suppressors. Genes Dev 2004;18:1909-1925

Ernkvist M, Luna Persson N, Audebert S, et al. The Amot/Patj/Syx signaling complex spatially controls RhoA GTPase activity in migrating endothelial cells. Blood 2009;113:244-253

Lee M and Vasioukhin V. Cell polarity and cancer--cell and tissue polarity as a non-canonical tumor suppressor. Journal Cell Sci 2008;121:1141-1150

Royer C and Lu X. Epithelial cell polarity: a major gatekeeper against cancer? Cell Death Differ 2011;18:1470-1477

Yi C, Troutman S, Fera D, et al. A tight junction-associated Merlin-angiomotin complex mediates Merlin's regulation of mitogenic signaling and tumor suppressive functions. Cancer Cell 2011;19:527-540

18 FNA-BASED PROFILING OF MRNA AND PROTEINS

Bo Franzén, Rolf Lewensohn

18.1 Background

Previous studies have proven the feasibility and biological validity of gene expression profiling of longitudinal FNA-samples during a phase III trial of metastatic breast cancer (Foukakis T et al 2015). We can now show that two recently established technology platforms (Nanostring technologies and proximity extension assay) can be applied for mRNA and protein profiling using a simplified protocol (Assarsson E et al 2014; Geiss GK et al 2008).

Our results show that it is possible to quantify, not only key markers such as ER, PR, HER2 and Ki67, but many immune related proteins in the minimal material from a single FNA sample (Franzén B et al, manuscript 2018). For example, CD8A, CD4, CD5, CD40, OX40, PDC1, PDL1, PDL2, IL6, IL8, CCL2, CCL3, CCL4, CCL20, CXCL5, CXCL9, CXCL10, CXCL11, CXCL17, CA9, FASL, GZMB, GZMA, GZMH and VEGFA, for complete list, see www.olink.com. We can confirm previously reported differences between ER negative *vs* positive BC and show in addition significant differences between BC subtypes regarding several chemokines previously never analysed in FNA samples from early BC.

Our results, complementary to the immunological profiling (section 18), pave the way for a simplified longitudinal FNA-based protein profiling of patients before, during and after treatment, providing new and possibly cost-effective tools to better understand which patients respond to neoadjuvant therapy, which patients that may be responsive to immunotherapy but also to evaluate therapy responses for patients with metastatic breast cancer.

18.2 Methods

In parallel to the planned sampling of blood and sampling by core needle biopsy (section 18.2) before, during and after chemotherapy, FNA samples will be obtained shortly *before* the core needle biopsy, see 24.3 Schedule for biological samples.

The FNA-sample needle (22 Gauge) is snap frozen in dry ice or liquid nitrogen and stored in the biobank. This needle should be obtained in parallel to a cytological sample where the quality of material and presence of tumour cells can be verified. Material from the extra needles will be extracted and analysed by proximity extension assay (PEA). The PEA assay panel "ImmunOncology" (www.olink.com) include several potential biomarkers related to this study [Goel S et al 2017]. We will also perform targeted gene expression profiling using NanoString technology on the same material. For the molecular profiling of QC approved samples, frozen needles will be thawed on ice and the material will be extracted into minimal volumes of RIPA buffer, supplemented with protease and RNAse inhibitors.

18.3 References

Assarsson E, Lundberg M, Holmquist G, et al. Homogenous 96-Plex PEA Immunoassay Exhibiting High Sensitivity, Specificity, and Excellent Scalability. PLoS One 2014;9(4):e95192.

Foukakis T, Lövrot J2, Sandqvist P, et al. Gene expression profiling of sequential metastatic biopsies for biomarker discovery in breast cancer. Mol Oncol 2015;9(7):1384-91.

Franzén B, et al. A fine needle aspiration-based protein signature discriminates benign from malignant breast lesions. 2018 (submitted, under revision).

Geiss GK, Bumgarner RE, Birditt B et al. Direct multiplexed measurement of gene expression with color-coded probe pairs. Nat Biotechnol 2008;26:317–325.

Goel S, DeCristo MJ, Watt AC et al. CDK4/6 inhibition triggers anti-tumour immunity. Nature 2017;548(7668):471-475.

19 IMMUNOLOGICAL PROFILING

Jana de Boniface, Rolf Kiessling, Andreas Lundqvist

19.1 Background

The existence of specific immune cells and antibodies directed against cancer has been described but also functional alterations favoring the disease. Tumor-induced immune dysfunction in several types of cancer is directly related to stage and prognosis, and worsens with disease progression (Matsuda et al 1995). Recently, the combination of several immunological factors ("immunological fingerprint") has been shown to be a stronger prognostic tool in colorectal cancer than the TNM classification which adjuvant treatments are based upon (Pages et al 2009 and 2010). Furthermore, tumors can induce and recruit immunosuppressive cells, such as regulatory T cells (Treg) and myeloid-derived suppressor cells (MDSC), to the tumor, the blood and to secondary lymphoid organs (Bronte and Mocellin 2009).

In fresh blood samples, we have recently shown that the expression of the zeta-chain, essential part of the T cell receptor (TCR) and activating NK receptors, is suppressed already in patients with early breast cancer. Loss of expression is paralleled with T cell dysfunction (de Boniface et al 2012). We have also shown that T cell differentiation is induced already at an early tumor stage (Poschke et al

2012): Breast cancer patients with lymph node metastases have more circulating CD8+ memory cells and fewer naive CD8+ cells than healthy controls. Up-regulation of CD69 and PD1 on T cells from cancer patients indicates previous activation, and increased expression of receptors CCR5 and CXCR3 on CD8+ T cells shows that their homing capacity is altered. Tumor progression and increased aggressiveness leads to an expansion of memory T cells over naive T cells, indicating immunologic exhaustion similar to that observed in immunosenescence. We have also previously described the down-regulation of TCR-zeta and CD28 in sentinel lymph nodes (SLN), functionally the first lymph node receiving lymphatic drainage from the breast tumor, as a signal of impaired T cell function (Schule et al 2002).

In an ongoing analysis on histological tumor and lymph nodes from the same material as above, we have observed significantly higher numbers of CD8+ and FoxP3+ cells and a higher FoxP3+/CD8+ ratio in SLNs than in primary tumor tissue. In case of lymphovascular invasion, these cell counts increase further. Compared with luminal A tumors, more aggressive tumor subtypes such as luminal B and basal-like tumors are characterized by higher numbers of tumor-infiltrating FoxP3+ cells and a higher FoxP3+/CD8+ ratio. This association is underscored by increasing numbers of both cell types and their ratio with higher histological grade.

Lymphocytic infiltration of the tumor may be a potent predictive factor of pathologic complete response in neoadjuvant chemotherapy (Denkert et al 2010; Ono et al 2012; Yamaguchi et al 2012). The combination of FoxP3- and CD8+ in the tumor and surrounding tissue after neoadjuvant chemotherapy may predict breast cancer survival more accurately than the conventional prognostic TNM classification (Ladoire et al 2011).

19.2 Methods

Fresh blood samples before and during treatment will be analyzed by multi-parameter flow cytometry regarding previously characterized subsets of T cells, especially regulatory T cells, loss of the TCR-zeta chain and markers of T cell differentiation.

Paraffin-embedded sections of core biopsies from the tumor before, during and after neoadjuvant chemotherapy will be analyzed using established single and double immunohistochemical staining with FoxP3, CD8, CD45RO and CD3-zeta, both at the infiltrative margin and central tumor locations. Paraffin-embedded sections from sentinel node biopsies before treatment will be analyzed regarding T cell parameters as above. It is estimated that 5-7 sections from each biopsy will suffice for these analyses.

The results from the analyses will then be correlated to clinical parameters, with special focus on the prediction of treatment response. Comparison of immunological profiling before and during treatment will be performed with the aim to identify immunological changes that may predict treatment response at an early stage.

Due to the time-dependent handling of fresh blood, the collection of material is limited to the Karolinska university hospital, Solna site!

19.3 References

Bronte V and Mocellin S. Suppressive influences in the immune response to cancer. J Immunother 2009;32(1):1-11

De Boniface J, Poschke I, Mao Y et al. Tumor-dependent down-regulation of the zeta-chain in T-cells is detectable in early breast cancer and correlates with immune cell function. Int J Cancer 2012;131(1):129-39

Denkert C, Loibl S, Noske A et al. Tumor-associated lymphocytes as an independent predictor of response to neoadjuvant chemotherapy in breast cancer. J Clin Oncol 2010;28(1):105-13

Matsuda M, Petersson M, Lenkei R et al. Alterations in the Signal-Transducing Molecules of T Cells and NK Cells in Colorectal Tumor-Infiltrating, Gut Mucosal and Peripheral Lymphocytes: Correlation with the Stage of the Disease. Int J Cancer 1995;61:765-772

Ono M, Tsuda H, Shimizu C et al. Tumor-infiltrating lymphocytes are correlated with response to neoadjuvant chemotherapy in triple-negative breast cancer. Breast Cancer Res Treat 2012;132(3):793-805

Pages F, Kirilovsky A, Mlecnik B et al. In situ cytotoxic and memory T cells predict outcome in patients with early-stage colorectal cancer. J Clin Oncol 2009;27(35):5944-51

Pages F, Galon J, Dieu-Nosjean MC et al. Immune infiltration in human tumors: a prognostic factor that should not be ignored. Oncogene 2010;29(8):1093-102

Poschke I, De Boniface J, Mao Y et al. Tumor-induced changes in the phenotype of blood-derived and tumor-associated T cells of early-stage breast cancer patients. Int J Cancer 2012;131(7):1611-20

Schule J, Bergkvist L, Hakansson L et al. Down-regulation of the CD3-zeta chain in sentinel node biopsies from breast cancer patients. Breast Cancer Res Treat 2002;74(1):33-40

Yamaguchi R, Tanaka M, Yano A et al. Tumor-infiltrating lymphocytes are important pathologic predictors for neoadjuvant chemotherapy in patients with breast cancer. Hum Pathol 2012;43(10):1688-94

20 PROFILING OF RECURRENCE/NEW PRIMARY DURING FOLLOW-UP

20.1 Background

Patients with large primary breast tumors with or without regional lymph node metastases have an increased risk of recurrence compared with patients with limited disease, approximately 30-50 % (Bear et al 2006, Kong et al 2011; Wu et al 2011; von Minckwitz et al 2011). Even without radiologically proven metastases, the risk of micrometastases is increased due to the advanced stage and biological properties. These patients are offered preoperative (neoadjuvant) chemotherapy with the intention to reduce the tumor burden in order to improve the chance for radical surgery and to reach disseminated tumor cells early in treatment. Neoadjuvant chemotherapy also allows for in-patient assessment of response, in contrast to postoperative adjuvant therapy.

The PREDIX LumB study combines the intention to investigate clinical response with studies on tumor biology. Tumor tissue and blood samples are collected before start, during and after termination of the neoadjuvant treatment. Patients treated within the framework of the PREDIX LumB study are followed for ten years according to the protocol.

Several trials have shown that routine predictive factors such as hormone receptors, proliferation markers and HER2 expression may differ between primary tumor and metastasis (Liedtke et al 2009, Wilking et al 2011). It is yet unclear, if the changes in tumor characteristics reflect transformation of the genome expression of the individual tumors, or indicate limited representativity of the tissue samples due to tumor heterogeneity.

20.2 Methods

In case of recurrence during follow-up, patients are asked to participate in a follow-up study, provided informed consent and access to a metastatic site/contralateral tumor by biopsy or fine-needle aspiration (FNA).

The purpose of this substudy is to investigate populations from metastatic sites which have resisted to and, thereby, survived the primary treatment. To investigate potential changes/selection of resistant cell groups, we intend to analyze samples from metastatic sites in patients previously treated within the PREDIX LumB trial, provided that the following conditions are met:

1. Local recurrence or distant metastases accessible for fine-needle aspiration (FNA). Tumor in the contralateral breast
2. Written informed consent
3. Previous consent to participate in the PREDIX LumB trial.

Tumor tissue is taken by FNA and immediately preserved in RNA*later*. In order to spare the patients an additional sampling, this procedure may be performed in connection with a diagnostic biopsy without having informed her about the study. If the patient after oral and written information disagrees, the sample will be destroyed.

The analyses include RNA microarray and DNA sequencing techniques.

Also presence of blood-borne growth factors and tumor-related DNA sequences which have been identified in connection with the primary tumor are important to detect and compare in the state of disseminated disease. Blood samples are collected and prepared for determination of circulating coagulant proteases (Tissue Factor, plasma), vascular growth factors and receptors VEGF (plasma), VEGF-R (serum) and VEGF-R1 (serum), proteomics (plasma), and circulating miRNA and DNA (plasma).

Tissue and blood samples are stored under the individual codes that are used in the PREDIX LumB-trial.

20.3 References

Bear HD, Anderson S, Smith RE, et al. Sequential preoperative or postoperative docetaxel added to preoperative doxorubicin plus cyclophosphamide for operable breast cancer: National Surgical Adjuvant Breast and Bowel Project Protocol B-27. J Clin Oncol 2006;24(13):2019-2027

Kong X, Moran MS, Zhang N, Haffty B, Yang Q. (2011) Meta-analysis confirms achieving pathological complete response after neoadjuvant chemotherapy predicts favourable prognosis for breast cancer patients. Eur J Cancer 2011, in press

Liedtke C, Broglio K, Moulder S, et al. Prognostic impact of discordance between triple-receptor measurements in primary and recurrent breast cancer. Ann Oncol 2009 20: 1953–1958

von Minckwitz G, Untch M, Nüesch E, et al. Impact of treatment characteristics on response of different breast cancer phenotypes: pooled analysis of the German neo-adjuvant chemotherapy trials. Breast Cancer Res Treat 2011 125:145–156

Wilking U, Karlsson E, Skoog L, et al. HER2 status in a population-derived breast cancer cohort: discordances during tumor progression. Breast Cancer Res Treat 2011 125:553–561

Wu, J., Li, S., Jia, W., Su, F. Response and prognosis of taxanes and anthracyclines neoadjuvant chemotherapy in patients with triple-negative breast cancer. J Cancer Res Clin Oncol. 2011 DOI 10.1007/s00432-011-1029-6

21 STUDY ADMINISTRATION

21.1 General rules

The study protocol has to be followed. Deviations from the protocol are not acceptable and have to be reported as protocol violations. Any essential modifications of the protocol are not valid before approval through the corresponding Ethics Committee and the Medical Product Agency.

21.2 Screening and enrolment

Potentially suitable patients will be screened by physicians or research nurses at the study sites in order to find eligible participants. The screening procedure aims at confirming that the inclusion and exclusion criteria are met.

21.3 Patient information and Informed Consent

Patients who fulfill inclusion and exclusion criteria must be provided with full and adequate verbal and written information about the objectives, the study outline and possible risks and benefits of participating in the study. They have the right to ask questions about the study and should be given adequate time to make the decision to participate in the study or not. The patients must be clearly informed that the data collected in the study will not identify individuals taking part in the study, following the Law in Personal Data and the EU Data Protection Directive.

The patients must be informed that participation is voluntary, and that they can decline further participation in the study at any time without stating any particular reason. The subjects should further be informed that a decision not to participate in the study or to withdraw will not be questioned or affect their future medical care or treatment at the clinic.

Written Informed Consent must be obtained from all participating subjects before enrolment in the study. The Informed Consent form should be signed at the same occasion by the investigator who gave the written and verbal information. The Informed Consent form has to be filed in the Investigator´s File and one copy has to be given to the patient. No trial-related procedures can take place before a written Informed Consent is obtained.

The subjects will consent a) to participate in the study, b) to allow regulatory authorities to gain full access to hospital records in order to control data collected in the study, c) to record, collect and process data and to store data in a database, and d) to store of tissue and blood samples in a biobank.

21.4 Registration

Patients who fulfil the criteria for inclusion are, after having given their oral and written consent, registered at the Clinical Trial Unit, KPE, Radiumhemmet, Karolinska Hospital, Stockholm, Sweden. Note: The registration form is sent to the CTO, KPE, Radiumhemmet, fax 08-306989.

An enrolment Log/Patient birth date will be filed at the center. The patient will receive the next consecutive code number in the trial and treatment arm according to randomisation.

21.5 Data management and study database

Data will be collected using an electronic system called PheedIt which is based on the SAS system provided by Stockholms läns landsting, and registered at the Clinical Trial Unit, KPE, Radiumhemmet, Karolinska Hospital, Stockholm, Sweden. A data management plan is delivered by

KPE, documenting the database and all procedures for the data management. The investigator must verify that all data entries in the CRFs are accurate and correct. If certain assessments according to the protocol are not performed for any reasons, or if certain information is not available, not applicable or unknown, this must be indicated in the CRF by the investigator. The investigator is required to sign off reported data. The study will be monitored by staff authorized by the CTO at KPE. A monitoring plan is set up to specify monitoring steps in detail.

21.6 Source data

In this study the patients' records will be regarded as source data with the exception of mammography and ultrasound and PET-CT data in which case the CRF will be regarded as source data.

21.7 Quality control and assurance

The Clinical Trial Unit (KPE), Radiumhemmet, Stockholm serves as the Clinical Trial Office (CTO) for the study. To ensure accurate, complete and reliable data according to GCP rules, the CTO is responsible for:

* Supply of instructional material to the study sites as appropriate
* Organization of start-up meetings to instruct the investigators and research nurses on the protocol, the completion of the case report forms, handling and transport of biological material and other study procedures
* Performance of periodic central and on-site monitoring visits to the study sites
* Consultation and continuous contact with the study site personnel by mail, telephone and /or fax
* Review and evaluation of case report form data by use of standard computer edits to detect errors in data collection.

The Investigator will permit study-related monitoring, audits, IEC review and regulatory inspection(s), providing direct access to source data/hospital records. The Investigator verifies that each subject has consented in writing to direct access to the original source data/hospital records by the use of written patient information and signed Informed Consent.

The data recorded in the CRFs will be controlled for consistency with the source data/hospital records during the monitoring (source data verification). Any discrepancies of data will be documented and explained in the monitoring reports.

21.8 Biobanking

Blood and tissue samples as well as unused cellular products will be stored in lockable liquid nitrogen tanks at CCK, R8:U1. This biobank will be part of the Karolinska Insitutet Biobank and will be stored in accordance with the regulations. The samples might be used for cancer and/or immunological related studies approved by the regional ethics committee. All stored samples will be coded and can only be connected to identifiable information via the password-protected coding sheet that will be in the possession of the investigator.

The subject retains the right to request that the sample material be destroyed by contacting the investigator. Following the request from the subject, the investigator is to provide the sponsor with the subject number so that any remaining blood and tumour samples and any other components from the cells can be located and destroyed.

21.9 Saving of medical records

The Investigator shall keep records of the study for 10 years to enable evaluations and inspections by regulatory authorities. This includes any original source data related to the study, the subject identification list (with subject numbers, full names and addresses) and the original signed Informed Consent, copies of all CRFs.

22 ETHICS

This study will be conducted in compliance with the protocol and in accordance with the ethical principles put forward in the Declaration of Helsinki (1) and in accordance with GCP rules (2).

The rights, safety and well-being of the trial subjects are the most important considerations and should prevail over interests of science and society.

Study personnel involved in conducting this trial will be qualified by education, training and experience to perform their respective tasks.

Tumor specimens collected for the biochemical analyses within the study will be codified. Personal codes are only known to the responsible physician. Study subjects can, at any time, withdraw their consent and the remains of the sample(s) donated will be destroyed.

1. World Medical Association Declaration of Helsinki, Ethical Principles for Medical Research involving human subjects. Available from: http://www.wma.net/e/policy/b3.htm
2. http://www.ich.org/fileadmin/Public_Web_Site/ICH_Products/Guidelines/Efficacy/E6_R1/Step4/E6_R1__Guideline.pdf

23 STATISTICS

Hemming Johansson

PREDIX is a study platform that contains four separate randomized phase II trials. The statistical design is similar for all four subgroups, but analyses will be performed separately.

The PREDIX LumB trial is based on current knowledge of molecular characteristics of breast cancer and designed primarily to investigate if response to endocrine treatment can be improved by inhibition of cdk 4 and 6 in comparison with standard chemotherapy, and, secondary, evaluate the impact of sequencing of the treatment options, find predictors of disease-free and overall survival, and observe toxicity, quality of life and changes in the surgical management in the two treatment groups.

The sample size is based on an explorative design: The primary objective is to compare clinical/radiological objective response *after completion of the first 12-week period* of neoadjuvant treatment.

Assuming an objective (partial or complete clinical/radiological) response after 12 courses of weekly paclitaxel (arm A) in approximately 30-40 % of cases, an absolute difference in response by 20 % between treatment alternatives A and B is considered clinically relevant. With alpha 0.10 (two-sided test) and power 0.80, 166 patients, 83 per treatment arm, are required. Differences in response rate will be tested using Fisher's exact test. The goal is to randomize 180 patients in the trial (Rubinstein et al 2005).

For patients with progressive disease (increase of tumor size by >20 %) at any time, individualized management, preferentially surgery, is the primary option.

Following the aims of the study, the statistical analyses will also

1. evaluate information obtained from imaging procedures, tumor biopsies and blood samples collected in connection with the treatment. The statistical methods will depend on the type of

characteristic studied and include t-tests, chi-square tests and, when applicable, non-parametric tests for significance testing.

2. relate event-free, invasive disease-free, distant disease-free, breast cancer-specific and overall survival to the biological characteristics collected during the treatment period. The statistical procedures include univariate and multivariate Cox proportional hazard models. Kaplan-Meier estimates will be used to plot survival-curves.
3. use multiple logistic regression analyses to evaluate predictive information from tissue and blood tumor markers and functional imaging procedures.

This randomized phase II trial has been set up with the aim to collect information that can be used to predict treatment response early during preoperative treatment. *Secondary objectives* are defined as follows:

Event-free survival (EFS) defined as time from date of randomisation to the first of any event including progression, loco regional or distant recurrence, contra-lateral breast cancer, other malignancy or death from any cause;

Invasive disease-free survival (IDFS) defined as time from date of surgery to the first appearance of loco-regional or distant recurrence, contra-lateral breast cancer, any cancer from other primary site or death from any cause;

Distant disease-free survival (DDFS) defined as time from date of surgery to the first appearance of incurable loco regional or distant metastases *or* death due to breast cancer without previous notification of dissemination;

(Breast cancer-specific survival defined as time from date of randomisation to death with breast cancer defined as primary cause)Overall survival (OS) defined as time from date of randomisation to death from any cause.

The PREDIX LumB-trial has an explicitly explorative design, but with the intention to motivate the induction of prospective randomized phase III trials with stringent statistical requirements using overall survival as endpoint. This will be stated clearly in the reports.

Reference

Rubinstein LV, Korn EL, Freidlin B, et al. Design issues of randomized phase II trials and a proposal for phase II screening trials. J Clin Oncol 2005;23:7199-206

24 PUBLICATION POLICY

Publication of the results will follow guidelines based on the recommendations of the Swedish Breast Cancer Group, based on the publication rules of the Scandinavian Breast Group (http://www.swebcg.se).

The Vancouver declaration (Br Med J: 296, 401-405, 1988) should be followed in all publications based on this study and the below rules are based on the Scandinavian Breast Cancer Groups (SBG) implementation of the Vancouver declaration.[1]

---

[1]

4. Major publications (i.e. overall trial results, or general articles submitted to journals that require review)
    * The study has a cooperative group defined as ”PREDIX Trialists' Group”, which includes actively participating physicians, scientists and statisticians. The study coordinators shall suggest to the Trialists' Group the formation of a Coordinating Group and a Writing Committee. It is recommended that these committees should be kept small and consist of less than 10 persons, coordinators and study statistician included. The members of the Writing Committee should fulfil the criteria for authorship as expressed in the Vancouver declaration[1]. The formation of a Writing Committee is heralded to the chairman. The above committee prepares a draft manuscript for circulation to all Trialists Group members. Participating centres and persons should preferably be mentioned in the acknowledgements or in an addendum.
    * If major problems occur in connection with the formation of the Writing Committee, these can be presented for the Coordinating Group, which then takes suitable action in order to solve the problems.
    * The authorship could be in the name of the group. If the preferred journal insists on named authors, the coordinator(s) (sometimes called "principal investigators") and the members of the Writing Committee will be these authors and the PREDIX Trialists' Group is presented in an addendum or in the acknowledgements.
5. Specialist (spin off-, satellite-) publications (i.e. specific aspects of a study protocol or its methodology, in some cases a parallel project performed by an external group)
    * The specialists will be the authors.
    * The relevant working party must be named e.g. "in collaboration with the PREDIX Trialists' Group".
    * Reference must be made to the paper(s) of the overall trial where all participants were named.
    * The appropriate writing committee must approve the final manuscript prior to submission. If conflict occurs the Coordinating Group's action may be taken.
2. Invited publications (i.e. where individual or a group of participants have been invited to submit a publication)
    * The author(s) will be the invitee plus those who assisted with the work.
    * The relevant working party must also be named, e.g. "in collaboration with the PREDIX Trialists Group".
    * Reference must be made to the paper(s) of the overall trial where all participants were named.

The selection of members of the writing committee must be such that it represents in a balanced way the participation in the study as an author. All members of the writing committee should fulfil the criteria for authorship expressed in the Vancouver declaration. The working party should for the selection process take into account loyalty and dedication to the project throughout all its phases. Patient numbers alone will not be the ultimately deciding factor for authorship. However, investigators from centres that have worked with low efficiency (<50 %) and with low patient numbers (<5 %) will only exceptionally be offered membership of the writing committee. Generally, investigators from centres with >10 % of included patients and high efficiency will be asked to participate in the writing committee if the person fulfils the Vancouver criteria. Centres with >30 % of included patients can be offered two positions in the writing committee.

* The appropriate writing committee must approve the final manuscript prior to submission. If conflict occurs the working party's action may be taken.

The submission to external meetings is encouraged. These must be circulated to the Coordinating Group for approval. If disapproval occurs the PREDIX Trialists Group's action may be taken as mentioned above. The PREDIX Trialists Group must be named e.g. "for the PREDIX Trialists Group". Study coordinators should be mentioned in the title. These rules for abstracts apply regardless of how the presentation is ultimately made and the text of this paragraph is also applicable to all the other presentations of data that has not been covered in the above text.

25 HANDLING OF TISSUE AND BLOOD SAMPLES

25.1 Tissue samples

In overview, five (at least 3) core biopsies are taken within 4 weeks before start and after 12 weeks of study treatment. Tissue is also obtained from the tumor shortly after removal by the surgeon. All core biopsies must be handled as follows:

1. Three of the core biopsies are transported on crushed ice, two of them are then frozen immediately, a third after pretreatment with protease inhibitor (see below).
2. Two of the core biopsies will be fixed in formalin and embedded in paraffin.

For priority in case of limited access to samples, see the "Manual".

For details, see below.

25.1.1 General handling of samples

General precautions:

Barrier protection should be used at all times to prevent contamination. During the entire processing of fresh tumor, the samples must not be touched without sterile gloves! There are plenty of RNAse enzymes on our fingers which may easily destroy the RNA.

For details of handling of the biopsy samples, see the **"Manual"**.

25.1.2 Biopsies for paraffin-embedded tissue (FFPE)

The formalin-fixed tissue derived from the core biopsy before start of treatment will be paraffin-embedded and used for diagnosis and IHC-characterization of prognostic and predictive factors.

Frozen tissue will be formalin-fixed 24-48 hours and paraffin-bedded thereafter. It is estimated that 10-15 sections from each biopsy will suffice for these analyses.

25.1.3 Biopsies for genomics

1. The first two of the biopsies are placed into separate sterile tubes and further handled as follows:
2. Keep on ice

3. Mark tube labelled with instructions according to the “Manual” (before start of treatment, after 12 weeks)
4. Store in -70°C.

25.1.4 Biopsy for proteomics

1. The biopsy is placed into a 15 ml Falcon tube with 5 ml PBS and protease inhibitor. Preparation: Add 1 tablet of Complete EDTA free protease inhibitor (Roche) to 10 ml of PBS
2. Keep on ice
3. Mark tube labelled with instructions according to the “Manual” (before start of treatment, after 12 weeks)
4. Prior to freezing: Discard PBS from the Falcon tube and leave the tumor tissue semi-dry in the tube
5. Store in -70°C.

The samples for proteomics should be handled with extra care:

Fast track: In proteomics we do not know which or what type of proteins will be discovered as potential biomarkers. Therefore, it is of great importance to keep all proteins intact and to handle the samples with the presumption that some proteins may be very labile. Cells and extracellular fluids like plasma and serum contain numerous proteases, i.e. enzymes that break down proteins rapidly. Samples for proteomics must be taken care of immediately in order to keep the time span from blood collection or tissue removal to freezing of the samples as short as possible.

Clean handling: The reproducibility depends on a correct handling of the samples. In proteomics, hundreds of different proteins are measured simultaneously and the results are evaluated statistically. If sample preparation introduces increased risk of experimental variation, the risk of false discoveries increases as well as the probability to miss clinically potential biomarkers. Therefore, the sample collection and handling methods must be strictly standardized during the process of sample collection for proteomic studies.

Tissue turnover results in degradation products as DNA, RNA, and proteins, or fragments of them which are dissolved in the plasma component of the blood. Possible interesting markers, like alterations in degradation products or reactive proteins occurring during disease, may be in low concentrations in blood, but undetectable with conventional methods including ELISA. In order to minimize known causes of variability, it is important to avoid differences, even subtle ones, in handling of the blood samples. There is international consensus that blood samples should be taken with EDTA as coagulation inhibitor. The protocol below represents the current state of the art of blood handling for proteomics.

25.1.5 Tissue from the surgical specimen

1. Divide the tumor into two open portions
2. Perform superficial scrapings using a scalpel as described in the “Manual”. Include the outer parts of the tumor
3. Place the material in three separate tubes for direct freezing. Note that protease inhibitor is not used for the surgical tissue sample.

25.2 Blood samples

25.2.1 Whole blood

The whole blood sample is drawn at baseline in a 4 ml EDTA tube. Samples may be stored at –70°C. They can be transported in room temperature but preferably on ice. Before storage, mark the tube carefully with patient ID, date and “SNP” or “Genomics”, respectively.

25.2.2 Plasma

General: Use EDTA as anticoagulant. **Note** that it is essential to invert the tube 10 (ten) times to distribute the anticoagulant.

1. Withdraw blood. Regarding amount of blood see the “Manual”
2. Note the time of collection
3. Place the tubes on ice
4. Register: date, patients study ID, time when sample was obtained. Note if any problems occurred in the proceeding, for example haemolysis
5. Centrifugate at 1500g/ RCF for 10 minutes to make sure plasma is free from filament and cells
6. Transfer plasma into cryotubes according to the **“Manual”**; avoid cells/precipitates in the bottom of the tube
7. Mark tubes carefully with instructions according to the **“Manual”**
8. Store at –70°C (if not available: –20°C for shorter time periods, <1 month).

25.2.3 Serum

Serum is derived from whole blood by coagulation (clotting) of blood cloth complement proteins and platelets followed by centrifugation to remove blood cells and coagulation precipitates. The coagulation of whole blood takes place spontaneously in the test tube at room temperature. Use a 5 ml SST tube (Serum Separator Tube).

1. Withdraw blood samples
2. Note the time of collection
3. Register: date, patient ID, time when sample was obtained. Note if any problems occurred in the proceeding for example haemolysis
4. Invert the tube 10 (ten) times
5. Allow the blood samples to clot for a minimum of 30 minutes and a maximum of 60 minutes at room temperature. If vacuum gel tubes are used the recommended temperature is 20-22°C as the gel viscosity changes in colder temperatures
6. Centrifuge at 1500g/RCF for 10 minutes at room temperature
7. Mark new clean tubes carefully with instructions according to the **“Manual”**
8. Transfer the upper light yellow part (serum) to the cryotubes, preferable aliquots of 0.5 ml in approximately four tubes; avoid cell/precipitates in the bottom of the tubes
9. Freeze immediately at preferably -70°C, but at least -20°C
10. Store at -70°C.

25.3 Schedule for biological samples

SCHEDULE FOR COLLECTION OF BIOLOGICAL SAMPLES			Before treat-ment	After 6 weeks	After 12 weeks	After 18 weeks	After 24 weeks	At surgery	End-of-treatment	Annual follow-up visits years 1-5	At recur-rence
**Core biopsies (14G needle)**			●		●						
**Tumor tissue (surgery)**								●			
**FNA**											●
**Blood samples**											
SNP 4 ml	WB	EDTA tube	●								
Genomics 4 ml	WB	EDTA tube	●								
Genomics 2 x 8.5 ml	P	PPT tube	●	●	●	●	●		●	●	●
Proteomics 4 ml	P	EDTA tube	●	●	●	●	●		●	●	●
miRNA/vRNA 3.5 ml	S	SST tube	●	●	●	●	●		●	●	●

WB: Whole blood FB: Fresh blood P: Plasma S: Serum; *: only at Karolinska, Solna site

26 SUMMARY OF TRANSLATIONAL STUDIES

Translational studies will be the focus of the trial as described in detail in Chapters 8-19 in the protocol. These studies will be conducted by collaborating researchers at Karolinska Institutet and will be coordinated by Associate Professor Theodoros Foukakis and his research group, the Principal Investigator Associate Professor Thomas Hatschek and the Study director Professor Jonas Bergh, all at the Department of Oncology-Pathology, Karolinska Institutet.

The predefined and approved analyses of tissue and blood samples collected in the trial as described in Chapter 25 will follow the principles described in the respective chapters of the protocol and will be modified whenever necessary to reflect the state-of-the-art methods at the time of the analysis.

Analysis of transcriptomics (Chapter 10), genomics (Chapter 10) and proteomics (Chapter 14) will use current bioinformatics methods and image-based analyses of radiology and pathology images will be assisted by artificial intelligence-based tools.

Following publication, omics and associated data will be made available in human data repositories to accelerate disease research and improve human health. Standard jurisdictional data sharing requirements will be followed, and no personal data will be shared.

Intra-tumor heterogeneity will be studied with single cell RNA sequencing methods as described in Chapter 12 and will be complemented with single cell DNA sequencing as well as *in situ* RNA sequencing of single cells in the tumors (spatial transcriptomics).

The correlative analyses with the clinical data and outcomes will be performed jointly by the study statistician at Karolinska University Hospital and the researchers at Karolinska Institutet responsible for each translational project. Only the clinical data necessary for the analysis will be shared following standard routines and Data Transfer Agreements between the study sponsor and Karolinska Institutet whenever applicable.

27 APPENDIX: STUDY SPECIFIC SAE FORM

For the Sponsor SAE Number	Date received, by	Date reported to Pfizer	For Pfizer AER Number

PREDIX LumB | **SAE page 1** | Onkologens KPE KAROLINSKA

Study ID: WI194287 **Centre** **Patient number**

INSTRUCTIONS: *Please fill in as much data as possible on both page 1 and page 2 and send this form by fax to the PREDIX Study Office, fax: +46-8-306989* ***within 24 hours after notification****. After the event has terminated or more information is available, complete the form and send a copy to PREDIX Study Office.* ***Keep the original at your site until monitoring.***

Investigator name	Country where event occured:	Type of report: Intial ☐ Follow up ☐
Treatment arm:	A ☐ B ☐	Week number of treatment: ________
Date of onset of SAE		Date of awareness by Investigator

Drug, route	Dose, units	Frequency	Start date	Stop date	Ongoing
Paklitaxel, i.v.	mg				☐
Palbociclib, p.o.	mg				☐
Tamoxifen, p.o.	mg				☐
Goserelin, s.c.	mg				☐
AI, p.o., name: ______________	mg				☐

Reason for considering the event serious:

Resulted in death ☐ Life-threatening ☐ (Prolonged) Hospitalisation ☐

Pers/Sign disability/incapacity ☐ Congenital abnormality ☐ Other event ☐ specify: ____________________

Event narrative

....

....

....

SAE term: .... CTC Grade1 ☐ 2 ☐ 3 ☐ 4 ☐ 5 ☐

Any reasonable possibility that the event is related to:

Paklitaxel: No ☐ Yes ☐ Palbociclib: No ☐ Yes ☐

Tamoxifen: No ☐ Yes ☐ Goserelin: No ☐ Yes ☐

Aromatase inhibitor: No ☐ Yes ☐

Action taken regarding Paklitaxel:	Withdrawn ☐	Reduced ☐	No change ☐	Unknown ☐
Action taken regarding palbociclib:	Withdrawn ☐	Reduced ☐	No change ☐	Unknown ☐
Action taken regarding tam/AI/goserelin:	Withdrawn ☐	Reduced ☐	No change ☐	Unknown ☐

Names and doses of drug(s) given to treat the SAE:

....

....

Other actions to treat the event: No ☐ Yes ☐

Describe: ....

Outcome: Recovered ☐ Recovered w sequelae ☐ Ongoing ☐ Unknown ☐ Death ☐

If dead: Date of death: .... Cause of death: ....

All dates as dd.mm.yyyy Page 1 PREDIX-LumB SAE-form v1, 17.2.2016

PREDIX LumB

SAE page 2

Onkologens KFE KAROLINSKA

Study ID: WI194287 **Centre** **Patient number**

Patient data Date of birth ____________

☐ Male ☐ Female Height ______ cm Weight _____ kg

Patient history *Provide relevant medical history below. Include other illnesses present at time of event, previous study emergent adverse event, and pre-existing medical conditions.*

Disease (specify)	Onset date	Stop date	Check if Ongoing	Pertinent details *(incl surgical procedures and dates)*
			☐	
			☐	
			☐	
			☐	

Concomitant drugs *List below concomitant drugs taken within 2 weeks before the event onset. Exclude all drugs only administered more than 2 weeks before the event, and any drug used to treat the event or taken after event onset.*

Drug name (trade and generic)	Reason for use	Route	Start date	Stop date	Check if Ongoing
					☐
					☐
					☐
					☐
					☐
					☐
					☐

Relevant tests *List only relevant confirmatory test results for event(s), for example, from blood tests, diagnostic imaging.*

Test	Date	Result	Units	Normal range Low	High	Comments

Signature of person completing the form	Date
Investigator's signature	Date
