# Supplementary material for: Neoadjuvant palbociclib and endocrine therapy versus chemotherapy in ER + /HER2- breast cancer: a randomized phase II trial
Source: Nat Commun. 2026 Apr 8;17:3403. doi: 10.1038/s41467-026-71452-6 (PMC13069018; doi:10.1038/s41467-026-71452-6)
Supplement: Supplementary file 2 — Reporting Summary [file 41467_2026_71452_MOESM2_ESM.pdf]

nature portfolio

Corresponding author(s): Alexios Matikas, Theodoros Foukakis

Last updated by author(s): Feb 10, 2026

Reporting Summary

Nature Portfolio wishes to improve the reproducibility of the work that we publish. This form provides structure for consistency and transparency in reporting. For further information on Nature Portfolio policies, see our Editorial Policies and the Editorial Policy Checklist.

Statistics

For all statistical analyses, confirm that the following items are present in the figure legend, table legend, main text, or Methods section.

n/a	Confirmed	
☐	☒	The exact sample size (*n*) for each experimental group/condition, given as a discrete number and unit of measurement
☐	☒	A statement on whether measurements were taken from distinct samples or whether the same sample was measured repeatedly
☐	☒	The statistical test(s) used AND whether they are one- or two-sided<br>*Only common tests should be described solely by name; describe more complex techniques in the Methods section.*
☐	☒	A description of all covariates tested
☐	☒	A description of any assumptions or corrections, such as tests of normality and adjustment for multiple comparisons
☐	☒	A full description of the statistical parameters including central tendency (e.g. means) or other basic estimates (e.g. regression coefficient) AND variation (e.g. standard deviation) or associated estimates of uncertainty (e.g. confidence intervals)
☐	☒	For null hypothesis testing, the test statistic (e.g. *F*, *t*, *r*) with confidence intervals, effect sizes, degrees of freedom and *P* value noted<br>*Give P values as exact values whenever suitable.*
☒	☐	For Bayesian analysis, information on the choice of priors and Markov chain Monte Carlo settings
☒	☐	For hierarchical and complex designs, identification of the appropriate level for tests and full reporting of outcomes
☐	☒	Estimates of effect sizes (e.g. Cohen's *d*, Pearson's *r*), indicating how they were calculated

Our web collection on statistics for biologists contains articles on many of the points above.

Software and code

Policy information about availability of computer code

Data collection	No code was used for data collection.
Data analysis	All analyses were performed using R version 4.4.3 and Stata version 17 (StataCorp, College Station, TX, USA).

For manuscripts utilizing custom algorithms or software that are central to the research but not yet described in published literature, software must be made available to editors and reviewers. We strongly encourage code deposition in a community repository (e.g. GitHub). See the Nature Portfolio guidelines for submitting code & software for further information.

Data

Policy information about availability of data

All manuscripts must include a data availability statement. This statement should provide the following information, where applicable:

- Accession codes, unique identifiers, or web links for publicly available datasets
- A description of any restrictions on data availability
- For clinical datasets or third party data, please ensure that the statement adheres to our policy

Individual-level clinical and molecular trial data cannot be made publicly available owing to restrictions imposed by the Regional Ethical Committee in Stockholm.
All group- or summary-level data underlying the figures and plots are provided in the accompanying Source Data Excel file, with relevant data organized across separate sheets.
Tumour and blood whole-exome sequencing data, as well as tumour RNA-sequencing data, generated in this study have been deposited in the Swedish National Data Service (SND) under SND ID: 2025-192 (https://researchdata.se/en/catalogue/dataset/2025-192/1) and are available under controlled access due to patient

privacy and ethical restrictions; access may be granted upon request and following approval of a data sharing agreement.

Research involving human participants, their data, or biological material

Policy information about studies with human participants or human data. See also policy information about sex, gender (identity/presentation), and sexual orientation and race, ethnicity and racism.

Reporting on sex and gender	Eligible patients were women and men over 35 years, diagnosed with ER positive (ER ≥ 10%) and HER2-negative BC, greater than 2 cm in size and/or node-positive. Finally, all recruited patients were women, either premenopausal or postmenopausal.
Reporting on race, ethnicity, or other socially relevant groupings	Collection of data regarding patients' race was not performed since we lacked ethical approval for this. However, considering the broad population coverage, it is highly likely that the study population is representative of the Swedish population. In Sweden universal health care and population screening with mammography is offered to all inhabitants regardless of citizenship, job status, socioeconomic status, insurance, race or any other factor. This ensures equal access to cancer care for everyone.
Population characteristics	Eligible patients were women and men over 35 years with Eastern Cooperative Oncology Group performance status 0 or 1, diagnosed with operable ER positive (ER ≥ 10%, in accordance with the Swedish National Care Program for breast cancer) and HER2-negative BC, greater than 2 cm in size and/or node-positive. Patients with at most two distant metastases that could be treated with curative intent could be enrolled. Adequate cardiac, renal and hepatic function, and no history of other malignancies during the past five years were required for inclusion to the study. All enrolled patients received neoadjuvant therapy followed by surgical resection.
Recruitment	PREDIX LumB is an academic, prospective, randomized, open-label, multicenter phase 2 trial which was conducted at 3 centers in Stockholm, Sweden (Karolinska University Hospital, Södersjukhuset and Capio S:t Göran Hospital). Between March 18th 2016 and July 29th 2021, 181 patients were enrolled in the trial. The intention-to-treat population comprises 179 patients.
Ethics oversight	The study was approved by the Regional Ethical Committee in Stockholm (dnr 2014/1492-31/4) and the Swedish Medical Product Agency. All patients provided written informed consent before inclusion. The study was conducted according to the Declaration of Helsinki and the principles of good clinical practice and was registered with EudraCT number 2014-000810-72.

Note that full information on the approval of the study protocol must also be provided in the manuscript.

Field-specific reporting

Please select the one below that is the best fit for your research. If you are not sure, read the appropriate sections before making your selection.

☒ Life sciences ☐ Behavioural & social sciences ☐ Ecological, evolutionary & environmental sciences

For a reference copy of the document with all sections, see nature.com/documents/nr-reporting-summary-flat.pdf

Life sciences study design

All studies must disclose on these points even when the disclosure is negative.

Sample size	The sample size was determined for an explorative comparison of objective response rates after 12 weeks of neoadjuvant treatment. Assuming a 40% ORR12 in arm A (12 courses of weekly paclitaxel), and a 20% absolute improvement in arm B, a two-sided alpha = 0.10 and 80% power required 166 patients (83 per treatment arm). The goal was thus to randomize between 180 and 200 patients in the trial. A total of 181 patients were enrolled in the trial.
Data exclusions	Two patients are excluded from all analyses, one because no protocol-mandated research core biopsy was obtained prior to treatment start and one because the results of the baseline computed tomography (CT) scans became available after treatment started and they showed disseminated disease. As such, the intention-to-treat (ITT) population comprises 179 patients.
Replication	External validation of the findings of the study was performed in the CORALLEEN, SCAN-B and I-SPY2 cohorts, as detailed in the manuscript. All attempts to replicate the CDKPredX biomarker were successful
Randomization	Patients were randomly assigned (1:1) into the two treatment groups described hereunder. Randomization was conducted at the Central Trial Office at Karolinska University Hospital by a web-based procedure (TENALEA, TransEuropean Network for Clinical Trial Services, Amsterdam, Netherlands). Random assignment was stratified by participating site. Random permuted blocks of different sizes (block size of 2 or 4) were used to allocate the patients to each treatment group.
Blinding	The study was not blinded, as it was a prospective, randomized, open-label clinical trial.

Reporting for specific materials, systems and methods

We require information from authors about some types of materials, experimental systems and methods used in many studies. Here, indicate whether each material, system or method listed is relevant to your study. If you are not sure if a list item applies to your research, read the appropriate section before selecting a response.

Materials & experimental systems

n/a	Involved in the study	
☐	☒	Antibodies
☒	☐	Eukaryotic cell lines
☒	☐	Palaeontology and archaeology
☒	☐	Animals and other organisms
☐	☒	Clinical data
☒	☐	Dual use research of concern
☒	☐	Plants

Methods

n/a	Involved in the study	
☒	☐	ChIP-seq
☒	☐	Flow cytometry
☒	☐	MRI-based neuroimaging

Antibodies

Antibodies used
For Ki67 immunohistochemistry, two antibody clones were used: SP6 (BioCare, dilution 1:100) and 30-9 (Ventana).

Validation
For SP6 clone: https://biocare.net/product/ki-67-rabbit-antibody/
For 30-9 clone: https://diagnostics.roche.com/global/en/products/lab/ki-67-30-9-confirm-rtd000792

Clinical data

Policy information about clinical studies
All manuscripts should comply with the ICMJE guidelines for publication of clinical research and a completed CONSORT checklist must be included with all submissions.

Clinical trial registration
The trial is registered at ClinicalTrials.gov (identifier NCT02603679).

Study protocol
The study protocol is provided as a supplementary file.

Data collection
PREDIX Lum B (NCT02603679) is an academic prospective randomized phase II trial conducted in three sites in Sweden. Between March 18th 2016 and July 29th 2021, 181 patients were enrolled in the trial. The intention-to-treat (ITT) population comprises 179 patients.

Outcomes
The primary endpoint of the study is the rates of locally assessed radiological response at 12 weeks (ORR12), defined as complete (complete resolution of all lesions) or partial response (reduction in size of at least 30% with no new lesions). Secondary efficacy endpoints include ORR at 24 weeks (ORR24); rates of locally assessed pCR, defined as absence of invasive carcinoma in the breast and axillary lymph nodes (ypT0/Tis, ypN0); pathologic response according to Residual Cancer Burden (RCB), event-free survival (EFS), defined as time from randomization to disease progression, disease recurrence (local, regional, or distant), contralateral BC, other malignancy, or death from any cause, whichever occurs first; relapse-free survival (RFS), defined as time from surgery to disease recurrence (local, regional, or distant), or death from any cause, whichever occurs first; and overall survival (OS), defined as time from randomization to death from any cause.

Plants

Seed stocks
Report on the source of all seed stocks or other plant material used. If applicable, state the seed stock centre and catalogue number. If plant specimens were collected from the field, describe the collection location, date and sampling procedures.

Novel plant genotypes
Describe the methods by which all novel plant genotypes were produced. This includes those generated by transgenic approaches, gene editing, chemical/radiation-based mutagenesis and hybridization. For transgenic lines, describe the transformation method, the number of independent lines analyzed and the generation upon which experiments were performed. For gene-edited lines, describe the editor used, the endogenous sequence targeted for editing, the targeting guide RNA sequence (if applicable) and how the editor was applied.

Authentication
Describe any authentication procedures for each seed stock used or novel genotype generated. Describe any experiments used to assess the effect of a mutation and, where applicable, how potential secondary effects (e.g. second site T-DNA insertions, mosiacism, off-target gene editing) were examined.
